# Benchmarking network propagation methods for disease gene identification

**DOI:** 10.1371/journal.pcbi.1007276

**Published:** 2019-09-03

**Authors:** Sergio Picart-Armada, Steven J. Barrett, David R. Willé, Alexandre Perera-Lluna, Alex Gutteridge, Benoit H. Dessailly

**Affiliations:** 1 B2SLab, Departament d’Enginyeria de Sistemes, Automàtica i Informàtica Industrial, Universitat Politècnica de Catalunya, CIBER-BBN, Barcelona, Spain; 2 Networking Biomedical Research Centre in the subject area of Bioengineering, Biomaterials and Nanomedicine (CIBER-BBN), Madrid, Spain; 3 Institut de Recerca Pediàtrica Hospital Sant Joan de Déu, Esplugues de Llobregat, Spain; 4 Research Statistics, GSK, Stevenage, United Kingdom; 5 Computational Biology and Statistics, GSK, Stevenage, United Kingdom; 6 GSK Vaccines, Rixensart, Belgium; La Jolla Institute for Allergy and Immunology, UNITED STATES

## Abstract

In-silico identification of potential target genes for disease is an essential aspect of drug target discovery. Recent studies suggest that successful targets can be found through by leveraging genetic, genomic and protein interaction information. Here, we systematically tested the ability of 12 varied algorithms, based on network propagation, to identify genes that have been targeted by any drug, on gene-disease data from 22 common non-cancerous diseases in OpenTargets. We considered two biological networks, six performance metrics and compared two types of input gene-disease association scores. The impact of the design factors in performance was quantified through additive explanatory models. Standard cross-validation led to over-optimistic performance estimates due to the presence of protein complexes. In order to obtain realistic estimates, we introduced two novel protein complex-aware cross-validation schemes. When seeding biological networks with known drug targets, machine learning and diffusion-based methods found around 2-4 true targets within the top 20 suggestions. Seeding the networks with genes associated to disease by genetics decreased performance below 1 true hit on average. The use of a larger network, although noisier, improved overall performance. We conclude that diffusion-based prioritisers and machine learning applied to diffusion-based features are suited for drug discovery in practice and improve over simpler neighbour-voting methods. We also demonstrate the large impact of choosing an adequate validation strategy and the definition of seed disease genes.

## Author summary

The use of biological network data has proven its effectiveness in many areas from computational biology. Networks consist of nodes, usually genes or proteins, and edges that connect pairs of nodes, representing information such as physical interactions, regulatory roles or co-occurrence. In order to find new candidate nodes for a given biological property, the so-called network propagation algorithms start from the set of known nodes with that property and leverage the connections from the biological network to make predictions. Here, we assess the performance of several network propagation algorithms to find sensible gene targets for 22 common non-cancerous diseases, i.e. those that have been found promising enough to start the clinical trials with any compound. We focus on obtaining performance metrics that reflect a practical scenario in drug development where only a small set of genes can be essayed. We found that the presence of protein complexes biased the performance estimates, leading to over-optimistic conclusions, and introduced two novel strategies to address it. Our results support that network propagation is still a viable approach to find drug targets, but that special care needs to be put on the validation strategy. Algorithms benefitted from the use of a larger -although noisier- network and of direct evidence data, rather than indirect genetic associations to disease.

This is a *PLOS Computational Biology* Benchmarking paper.

## Introduction

The pharmaceutical industry faces considerable challenges in the efficiency of commercial drug research and development [1] and in particular in improving its ability to identify future successful drug targets.

It has been suggested that using genetic association information is one of the best ways to identify such drug targets [2]. In recent years, a large number of highly powered GWAS studies have been published for numerous common traits (for example, [3, 4]) and have yielded many candidate genes. Further potential targets can be identified by adding contextual data to the genetic associations, such as genes involved in similar biological processes [5, 6]. Biological networks and biological pathways can be used as a source of contextual data.

Biological networks are widely used in bioinformatics and can be constructed from multiple data sources, ranging from macromolecular interaction data collected from the literature [7] to correlation of expression in transcriptomics or proteomics samples of interest [8]. A large number of interaction network resources have been made available over the years, many of which are now in the public domain, combining thousands of interactions in a single location [9, 10]. They are based on three different fundamental types of data: (1) data-driven networks such as those built by WGCNA [8] for co-expression; (2) interactions extracted from the literature using a human curation process as exemplified by IntAct [11] or BioGRID [12]; and (3) interactions extracted from the literature using text mining approaches [13].

On the other hand, a plethora of network analysis algorithms are available for extracting useful information from such large biological networks in a variety of contexts. Algorithms range in complexity from simple first-neighbour approaches, where the direct neighbours of a gene of interest are assumed to be implicated in similar processes [14], to machine learning (ML) algorithms designed to learn from the features of the network to make more useful biological predictions [15].

One broad family of network analysis algorithms are the so-called Network Propagation approaches [16], used in contexts such as protein function prediction [17], disease gene identification [16] and cancer gene mutation identification [18]. In this paper, we perform a systematic review of the usefulness of network analysis methods for the purpose of identification of disease genes. As further explained in Methods, we define our test set of disease genes as genes for which the relationship with a disease was sufficiently clear to justify the start of a drug development programme. Claims that such methods are helpful in that context have been made on numerous occasions but a comprehensive validation study is lacking. One major challenge in doing such a study is to define a list of true disease genes for this purpose.

To address this, the Open Targets collaboration between pharmaceutical companies and public institutions collects information on known drug targets to help identify new ones [19]. A dedicated internet platform provides a free-to-use accessible resource summarising known data on gene-disease relationships from a number of data sources, like known released drugs and genetic associations from GWAS [19].

The purpose of this work is to quantify the performance of network propagation methods to prioritise novel drug targets, using various networks and validation schemes, and aiming at a faithful reflection of a realistic drug development scenario. We are not predicting gene targets for specific drugs, but rather sensible genes to target for a specific disease. Data on actual compounds targeting a gene is ignored: as long as the gene has been targeted by one or more compounds reaching the clinical trials, it is considered a sensible drug target. We select a number of network propagation approaches that are representative of several classes of algorithms, and test their ability to recover known target genes for several non-cancerous diseases by cross-validation.

We benchmark multiple definitions of disease genes as input for the prioritisers, computational methods, biological networks, validation schemes and performance metrics. We account for all possible combinations of such factors and derive guidelines for future disease target identification studies. The code and data that support our conclusions can be found in https://github.com/b2slab/genedise.

## Results

### Benchmark framework

**Fig 1:**
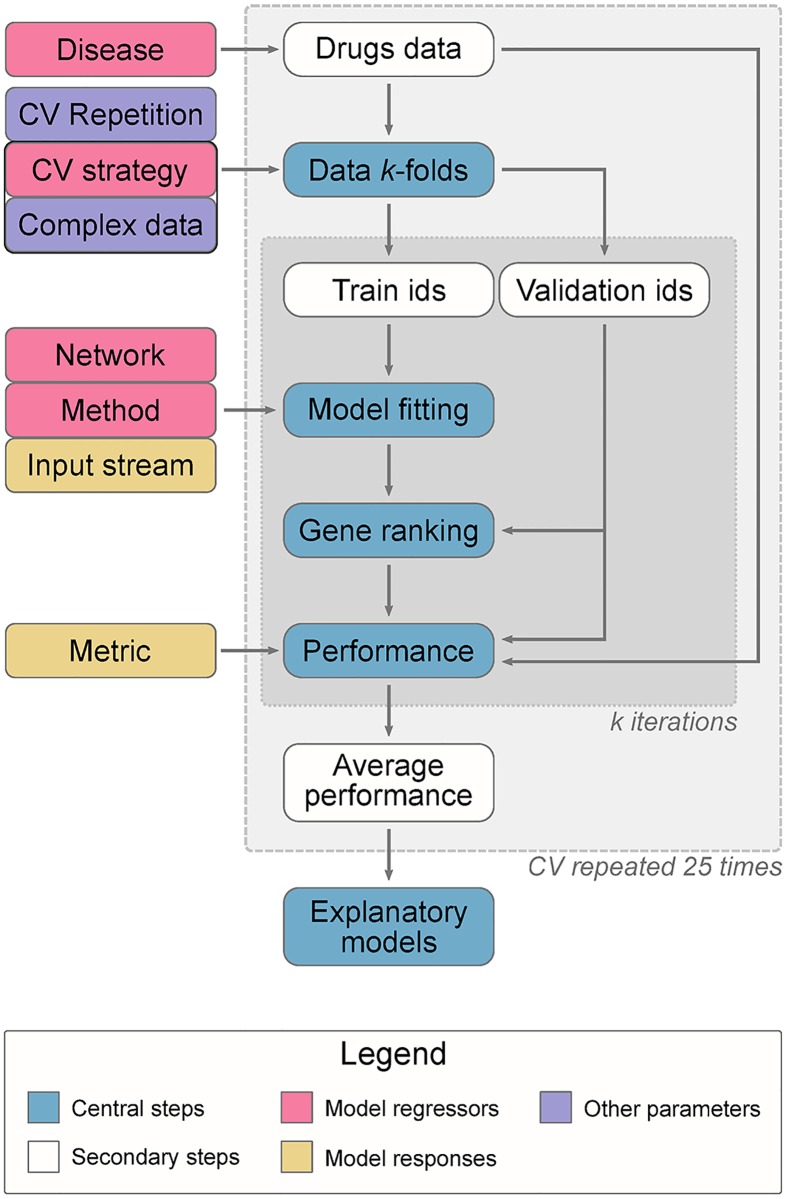
Benchmark overview. This work describes six performance metrics using two input streams (genetic association and drug-based genes) to predict drug target-based genes for 22 common diseases. 3-fold cross-validation (CV), repeated 25 times, was run under three CV strategies. The gene identifiers in each fold are determined using only the drugs data, regardless of the input. Two validation strategies are complex-aware and therefore needed this data to define the splits. 15 methods based on network propagation (including 4 baselines) were evaluated, using two networks with different properties, by modelling their performance -averaged on every CV round- with explanatory models. After obtaining the performance metrics, the explanatory models allowed hypothesis testing and a direct performance comparison between diseases, CV strategies, networks and methods, by setting them as the independent variables of the models. The latter is depicted by pink (independent variables) and yellow (dependent variable) blocks, and should not be confused with the “model fitting” block, which refers to the network propagation prioritisers.

Our general approach, summarised in Fig 1, consisted in using a biological network and a list of genes with prior disease-association scores as input to a network propagation approach. We tested some variations of classical network propagation -ppr, raw, gm, mc and z- which differ on the directedness of the propagation, the input weights and the presence of a statistical normalisation of the scores. Semi-supervised methods included, under the positive-unlabelled learning framework: knn and wsld. Both work directly on a graph kernel, closely related to network propagation. Supervised methods were also considered: COSNet, which regards the network as an artificial neural network, bagsvm, a bagging Support Vector Machine on a graph kernel, and rf and svm, which apply either Random Forest or a Support Vector Machine to network-based features that encode propagation states in a lower dimensionality. The EGAD method, based on neighbour voting, served as a baseline prioritiser. Three input-naïve baselines were included: pr and randomraw, both biased by the network topology, and random, a purely random prioritiser.

We used three cross-validation schemes -two take into account protein complexes- in which some of the prior disease-association scores are hidden. The desired output was a new ranking of genes in terms of their association scores to the disease. Such ranking was compared to the known target genes in the validation fold using several performance metrics. Given the amount of design factors and comparisons, the metrics were analysed through explanatory additive models (see Methods). Specifically, regression models explained the performance metrics (dependent variable) as a function of the prediction method, the cross-validation scheme, the network and the disease (regressors). This enabled a formal analysis of the impact of each factor on overall performance while correcting for the others. Alternatively, we provide plots on the raw metrics in S1 Appendix, stratified by method in Figures J and K or by disease in Figures L and M.

We considered 2 metrics (AUROC and top 20 hits) and 2 input types (known drug target genes and genetically associated genes), resulting in a total of 4 combinations, each described through an additive main effect model. Another 4 metrics were explored and can be found in Figure Q and Tables F and G in S1 Appendix.

Interaction terms within the explanatory models were explored, but they did not provide any added value for the extra complexity, see Figure S in S1 Appendix.

### Performance using known drug targets as input

**Fig 2:**
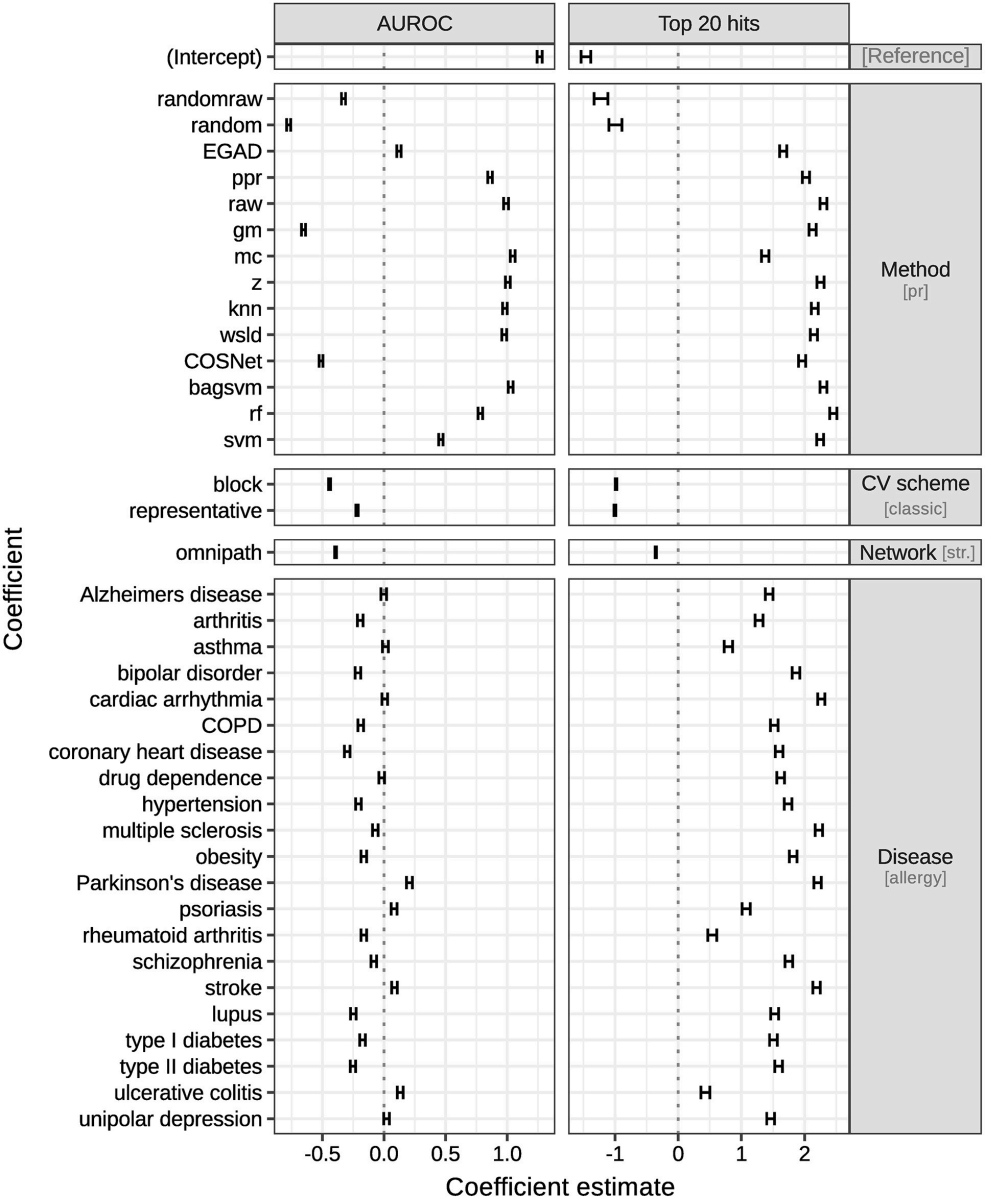
Additive explanatory models for AUROC and top 20 hits. Each column corresponds to a different model, whereas each row depicts the 95% confidence interval for each model coefficient. Rows are grouped by the categorical variable they belong to: method, cv scheme, network and disease. Each variable has a **reference level**, implicit in the intercept and specified in brackets: pr method, **classic** validation scheme, **STRING** network and **allergy**. Positive estimates improve performance over the reference levels, whereas negative ones reduce it. For example, the data suggest that method rf performs better than the baseline using both metrics, and is the preferred method using the top 20 hits. Switching from STRING to the OmniPath network, or from classic to block or representative cross-validation, has a negative effect on both performance metrics. Specific model estimates and confidence intervals can be found in Tables H and I in S1 Appendix.

Fig 2 describes the additive models for AUROC and top 20 hits, and using known drug targets as input. Note that the disease was included as a regressor in the explanatory models for further discussion. This was possible given our definition of drug targets: methods had to predict whether a gene has been targeted by any drug for a particular disease, implying that metrics were available at the disease level.

**Fig 3:**
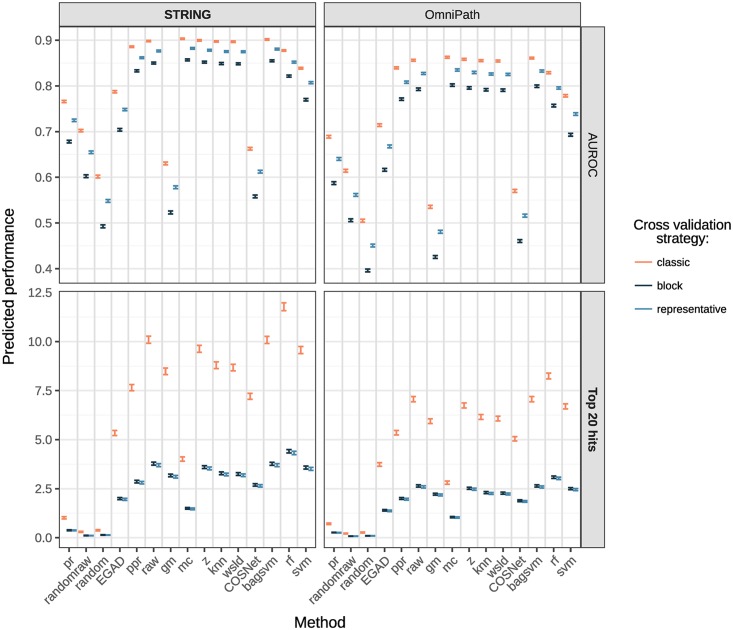
Performance predicted for AUROC and top 20 hits through the additive explanatory models. Each row corresponds to a different model and error bars depicts the 95% confidence interval of the additive model prediction, averaging over diseases. In bold, the main network (STRING) and metric (top 20 hits). The exact values can be found in Table I in S1 Appendix.

Fig 3 contains their predictions for each method, network and cross-validation scheme with 95% confidence intervals, averaged over diseases. The models are complex and we therefore review each main effect separately.

For interpretability within real scenarios, the top 20 hits is regarded as the reference metric in the main body. The standard AUROC (quasi-binomial) clearly led to different conclusions and is kept throughout the results section for comparison. The remaining metrics (AUPRC, pAUROC 5%, pAUROC 10% and top 100 hits) result in similar method prioritisations as top 20 hits, see Figure Q in S1 Appendix. Detailed models can be found in S1 Appendix, indexed by Tables F and G.

#### Comparing cross-validation schemes

Whether protein complexes were properly taken into account when performing the cross-validation (see Methods) stood out as a key influence on the quality of predictions: there was a dramatic reduction in performance for most methods when using a complex-aware cross-validation strategy. For instance, method rf applied on the STRING network dropped from almost 12 correct hits in the top 20 predicted disease genes when using our *classic* cross-validation scheme down to fewer than 4.5 when using either of our complex-aware cross-validation schemes. Likewise, Table E in S1 Appendix ratifies that only the *classic* cross-validation splits complexes. A recent study raised analogous concerns on estimating the performance of supervised methods when learning gene regulatory networks [20]. Random cross-validation would lead to overly optimistic performances when predicting new regulatory contexts, requiring to control for the distinctness between the training and the testing data. This confirms that other areas in computational biology may benefit from adjusted cross-validation strategies.

Our data suggests that the performance drop when choosing the appropriate validation strategy is comparable to the performance gap of competitive methods versus a simple neighbour-voting baseline EGAD (see Fig 2). This highlights the importance of carefully controlling for this bias when estimating the performance of target gene prediction using network propagation. Overall, the *classic* cross-validation scheme gave biased estimates in our dataset, whereas our *block* and *representative* cross-validation schemes had similar effects on the prediction performance. Method ranking was independent of the cross-validation choice thanks to the use of an additive model. Since both the *block* and *representative* schemes led to the same conclusions, we chose to focus on results from the block scheme in the rest of this study.

#### Comparing networks

We found that using STRING as opposite to OmniPath improved overall performance of disease gene prediction methods. Our models for top 20 hits quantified this effect as noticeable although less important than that of the cross-validation strategy. For reference, method rf obtains about 3 true hits under both complex-aware strategies in OmniPath. It has been previously shown that the positive effect on predictive power of having more interactions and coverage in a network can outweigh the negative effect of increased number of false positive interactions [21], which is in line with our findings. The authors also report STRING among the best resources to discover disease genes, which is analogous to our findings on the drug targets.

We focus on the STRING results in the rest of the text.

#### Comparing methods

Having identified the optimal cross-validation scheme and network for our benchmark in the previous sections, we quantitatively compared the performance of the different methods.

First, network topology alone had a slight predictive power, as method pr (PageRank approach that ignores the input gene scores) showed better performance than the random baseline under all the metrics. The randomised diffusion randomraw lied between random and pr in performance, depending on the metric. Both facts support the existence of an inherent network topology-related bias among target genes that benefits diffusion-based methods. This finding is compatible with the existence of a reduced set of critical edges that account for most of the predictive power in GBA methods [22], as highly connected genes are more likely to be involved in those.

Second, the basic GBA approach from EGAD had an advantage over the input-naïve baselines pr, randomraw and random. It also outperformed prioritising genes using other Open Targets data stream scores such as genes associated to disease from pathways, gene expression or animal models, while being comparable to the literature stream (see Table S in S1 Appendix).

**Fig 4:**
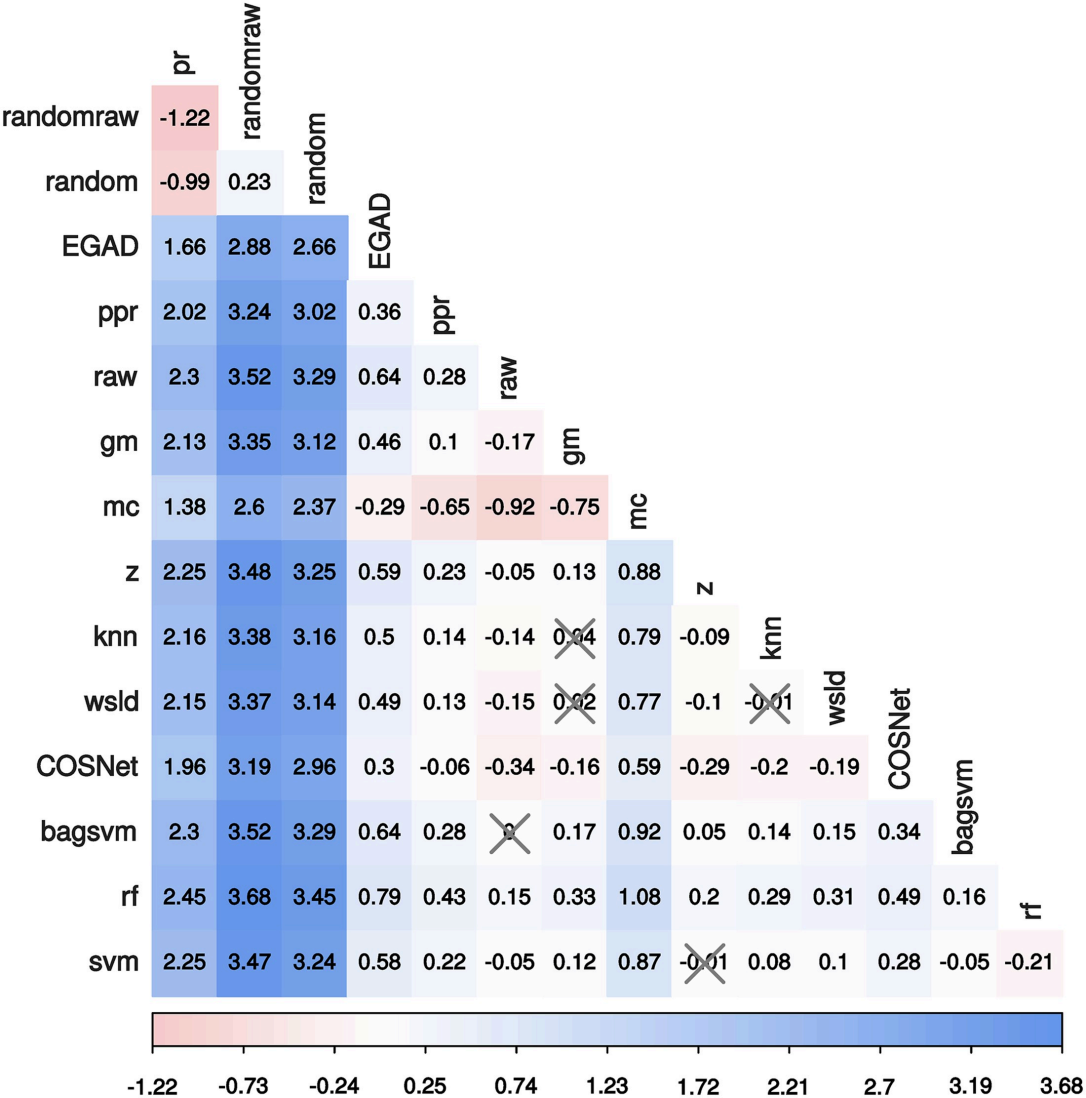
Pairwise contrasts on top 20 hits predicted by the main quasipoisson explanatory model. Differences are expressed in the model space. Most of the pairwise differences are significant (Tukey’s test, p <0.05) – non-significant differences have been crossed out.

**Fig 5:**
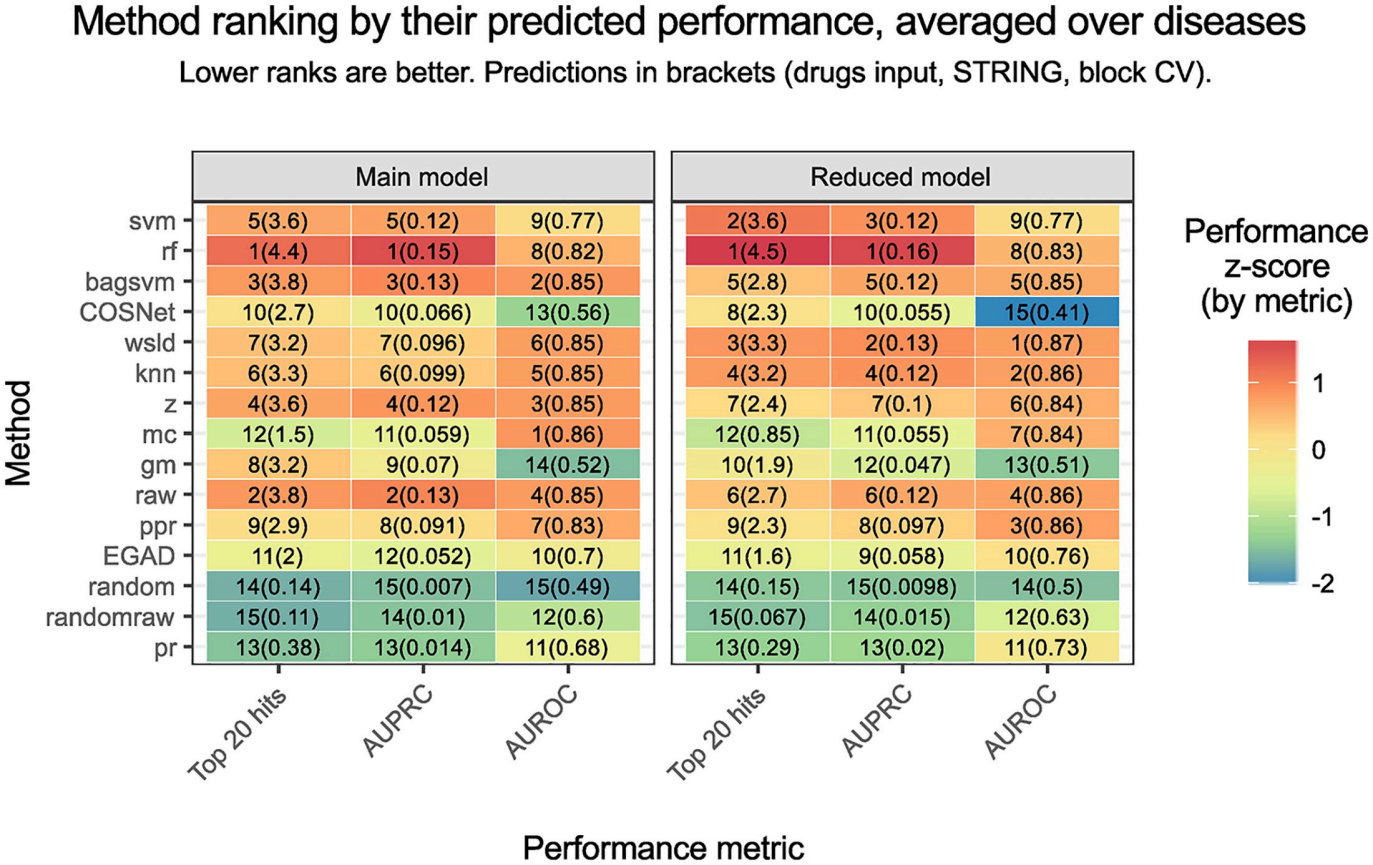
Ranking of all the methods. Ranking according to the predictions of the main explanatory models (left) and the reduced explanatory models within the STRING network and block cross-validation (right), in both cases on the drugs input and averaging over diseases. The main models serve as a global description of the metrics, whereas the reduced models are specific to the scenario of most interest. A column-wise z-score on the predicted mean is depicted, in order to illustrate the magnitude of the difference. Note how the top 20 hits and the AUPRC metrics lead to similar conclusions, as opposed to AUROC.

Most diffusion-based and ML-based methods outperformed EGAD. To formally test the differences between methods, we carried a Tukey’s multiple comparison test on the model coefficients (Fig 4) as implemented in the R package multcomp [23]. Although such differences were in most cases statistically significant after multiplicity adjustment, their actual effect size or magnitude can be modest in practice, see Figs 3 and 5. Results from top 20 hits suggest using rf for the best performance followed by, in order: raw and bagsvm, z and svm (main models panel in Fig 5).

The ranking of methods was similar when using the metrics AUPRC, pAUROC and top *k* hits (see Figure Q in S1 Appendix) and is only intended to be a general reference, given the impact of the problem definition, cross-validation scheme and the network choice.

With AUROC on the other hand, rf lost its edge whilst most diffusion-based and ML-based methods appeared technically tied. Despite its theoretical basis, interpretability and widespread use in similar benchmarks, these results support the assertion that AUROC is a sub-optimal choice in drug discovery practical scenarios.

**Fig 6:**
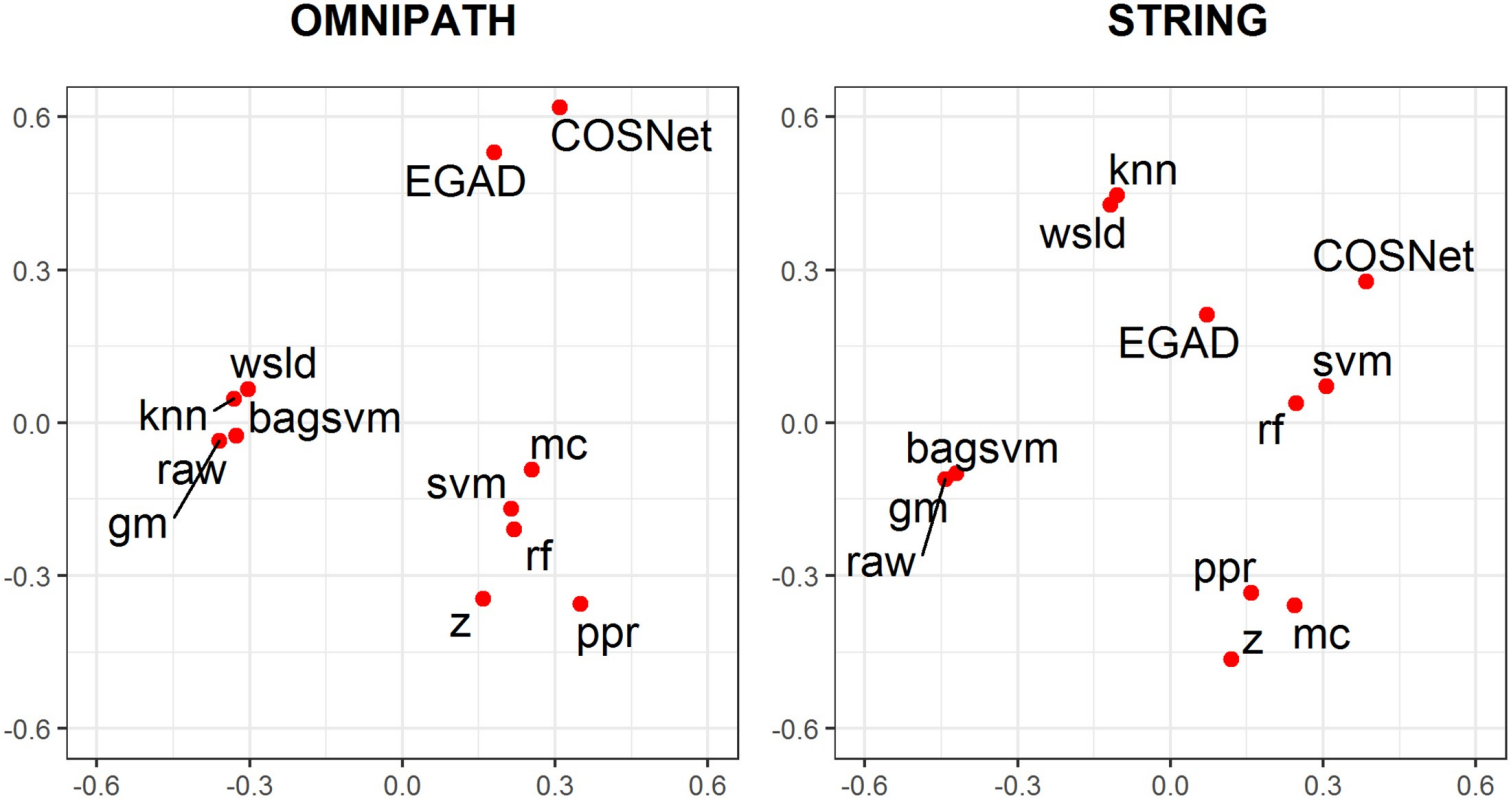
Multi-view MDS plot displaying the preserved Spearman’s footrule distances between methods. The differential ranking of their top 100 novel predictions using known drug target inputs are taken into account across all 22 diseases. Results are shown separately for the 2 networks considered in this study. Seed genes are excluded from the distance calculations.

Fig 6 further shows how the different methods compare with one another. Distances between each pair of method in terms of their top 100 novel predictions were represented graphically. We observe that the supervised bagged Support Vector Machine approach (bagsvm) behaves similarly to the simple diffusion approach (raw), reflecting the fact that they use the same kernel. We also observe that diffusion approaches do not necessarily produce similar results; for instance, raw and z. Besides, methods EGAD (arguably one of the simplest) and COSNet (arguably one of the most complex) seemed to result in similar predictions. Fully supervised and semi-supervised approaches largely group in the top right hand quadrant of the STRING plot away from diffusion methods, possibly showing better learning capability with the larger network.

When comparing overall performances shown in Fig 5 with the prediction differences from the MDS plot (Fig 6), the best methods owed their performance to different reasons as they do not occur within the same region of the plot (e.g. rf and raw). MDS plots on the eight possible combinations of network, input type and inclusion of seed genes are displayed in Figures O and P in S1 Appendix.

Focusing only on the STRING network and the block validation scheme, we fitted six additive explanatory models, called the reduced models, to model the six metrics for the drugs data input as a function of the method and the disease (see Table G in S1 Appendix). Methods were prioritised according to their main effects (Fig 5). The reduced models better described this particular scenario, as they were not forced to fit the trends in all networks and validation schemes in an additive way. Considering the top 20 hits, rf and svm were the optimal choices, followed by wsld and knn.

#### Comparing diseases

**Fig 7:**
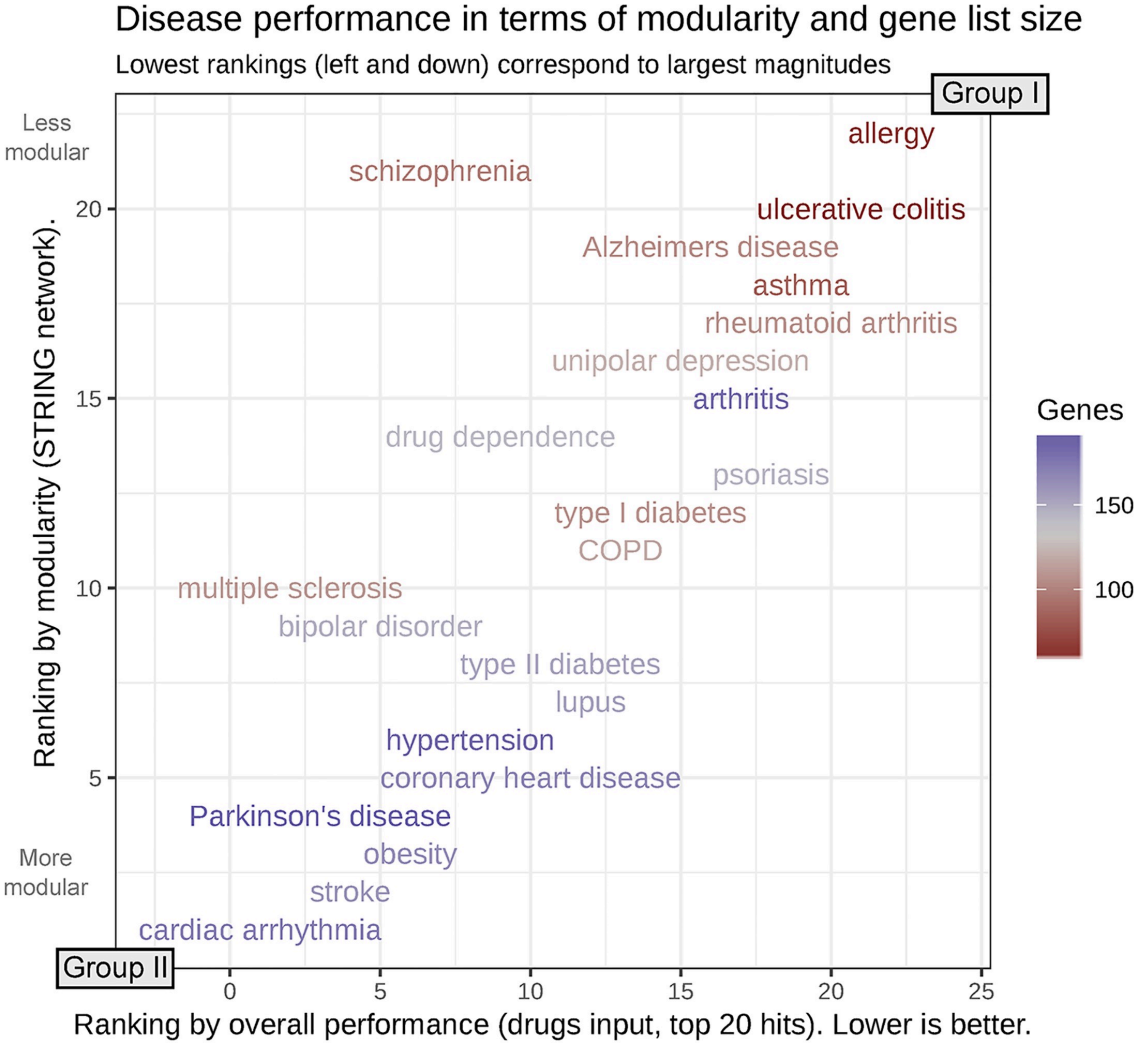
Disease performance in terms of input size and modularity. Disease performance ranked by the number of known target genes and their modularity (obtained using the igraph package, see Figure F in S1 Appendix). Modularity is a measure of the tendency of known target genes to form modules or clusters in the network. Diseases have been ranked using their explanatory model coefficient from the top 20 hits metric with known drug targets as input (x axis) and their modularity (y axis). As discussed in the text, best predicted diseases tend to have longer gene lists and be highly modular.

The top 20 hits model in Fig 2 shows that allergy (the figure’s baseline reference), ulcerative colitis and rheumatoid arthritis (group I) are the diseases for which prediction of target genes was worst, whereas cardiac arrhythmia, Parkinson’s disease, stroke and multiple sclerosis (group II) are those for which it was best. As shown in Fig 7, group I diseases had fewer known target genes and lower modularity compared to group II diseases.

Prediction methods worked better when more known target genes were available as input in the network, with two possible underlying reasons: the greater data availability to train the methods, and the natural bias of top 20 hits towards datasets with more positives. Likewise, a stronger modularity within target genes justifies the guilt-by-association principle and led to better performances. In turn, the number of genes and the modularity were positively correlated, see Figure N in S1 Appendix.

### Performance using genetic associations as input

Using genetically associated genes as input to a prediction approach to find known drug targets mimicked a realistic scenario where novel genetic associations are screened as potential targets. However, inferring known drug targets through the indirect genetic evidence posed problems to prediction strategies, especially those based on machine learning. Learning is done using one class of genes in order to predict genes that belong to another class, and the learning space suffers from intrinsic uncertainties in the genetic associations to disease. Both classes are inherently different: certain genes can be difficult to target, and a gene does not require to have been formally associated genetically to a disease to become a valid target.

Consequently, we observed a major performance drop on all the prioritisation methods: using any network and cross-validation scheme, the predicted top 20 hits were practically bounded by 1. This was more pronounced on supervised machine learning-focused strategies, as rf and svm lost their edge on diffusion-based strategies. The fact that the genetic associations of the validation fold were hidden further hindered the predictions and can be a cause of our pessimistic performance estimates.

#### Comparing cross-validation schemes

For reference, we also ran all three cross-validation schemes on the genetic data to quantify and account for complex-related bias. The models confirm that, contrary to the drugs-related input, the differences between the results for the different cross-validation schemes were rather modest. For example, method raw with the STRING network attains 0.59-0.64, 0.50-0.54 and 0.37-0.40 hits in the top 20 under the classical, block and representative cross-validation strategies. The slightly larger negative effect on top 20 hits observed with the representative scheme is expected because the number of positives that act as validation decreased and this metric is biased by the class imbalance. The agreement between method ranking using AUPRC and top 20 hits was less consistent, possibly due to the performance drop, whilst AUROC yielded a noticeably different ranking again. Further data can be found in Tables O and P in S1 Appendix.

#### Comparing networks

The change in performance for using the OmniPath network instead of the filtered STRING network was also limited. For AUROC the effect was negative, whereas for the top 20 hits metric the performance improved. Method raw changed from 0.50-0.54 top 20 hits in STRING to 0.61-0.66 in OmniPath under the block validation strategy.

#### Comparing methods

To be consistent with the drugs section, we take as reference the block cross-validation strategy and the STRING network.

The baseline approach pr that effectively makes use of the network topology alone proved difficult to improve upon, with 0.43-0.47 expected true hits in the top 20. Methods raw and rf respectively achieved 0.50-0.54 and 0.23-0.26 – although significant, the difference in practice would be minimal. The best performing method was mc with 0.65-0.7 hits. All the performance estimates can be found in Table P in S1 Appendix. To give an idea of the effort that would be required in a realistic setting to find novel targets, the number of correct hits in the top 100 hits was 3.29-3.45 with the best performing method (in this case, ppr), against 2.25-2.38 of pr.

Two main conclusions can be drawn from these results. First, the network topology baseline retained some predictive power upon which most diffusion-based methods, as well as machine-learning approaches COSNet and bagsvm, only managed to add minor improvements, if any. Second, drug targets could still be found by combining network analysis and genes with genetic associations to disease, but with a substantially lower performance and with a marginal gain compared to a baseline approach that would only use the network topology to find targets (e.g. by screening the most connected genes in the network).

It is worth noting that gene-disease genetic association scores themselves have drawbacks and that better prediction accuracy could result as genetic association data improves.

## Discussion

We performed an extensive analysis of the ability of several approaches based on network propagation to identify novel non-cancerous disease target genes. We explored the effect of various choices in factors including the biological network, the definition of disease genes acting as seeds, and the statistical framework being used to evaluate methods performance. We show that carefully choosing an appropriate cross-validation framework and suitable performance metric has an important effect in evaluating the utility of these methods.

Our main conclusion is that network propagation seems effective for drug target discovery, reflecting the fact that drug targets tend to cluster within the network. This may be due to the fact that the scientific community has so far been focusing on testing the same proven mechanisms, which can induce some ascertainment bias.

In a strict cross-validation setting, we found that even the most basic guilt-by-association method was useful, with ∼2 correct hits in its top 20 predictions, compared to ∼0.1 when using a random ranking. The best diffusion based algorithm improved that figure to ∼3.75, and the best overall performing method was a random forest classifier on network-based features (∼4.4 hits). Leading approaches can be notably different in terms of their top predictions, suggesting potential complementarity. We found a better performance when using a network with more coverage at the expense of more false positive interactions. In a more conservative network, random forest performance dropped to ∼3.1 hits. Comparing performance on different diseases shows that the more known target genes, and the more clustered these are in the network, the better the performance of network propagation approaches for finding novel targets for it.

We also explored the prediction of known drug target genes by seeding the network with an indirect data stream, in particular, genetic association data. Here, the best performing methods were diffusion-based and presented a statistically significant, but marginal, improvement over approaches that only look at network centrality.

We conclude that network propagation methods can help identify novel targets for disease, but that the choice of the input network and the seed scores on the genes needs careful consideration. Based on our approach and endorsed benchmarks, we recommend the use of methods employing representations of diffusion-based information (the MashUp network-based features and the diffusion kernels), namely random forest, the support vector machine variants, and raw diffusion algorithms for optimal results.

## Materials and methods

### Selection of methods for investigation

Network propagation algorithms were selected for validation based on the following criteria:

1. Published in a peer-reviewed journal, with evidence of improved performance in gene disease prediction relative to contenders.
2. Implemented as a well documented open source package, that is efficient, robust and usable within a batch testing framework.
3. Directly applicable for gene disease identification from a single gene or protein interaction network, without requiring fundamental changes to the approach or additional annotation information.
4. Capable of outputting a ranked list of individual genes (as opposed to gene modules, for example).

In addition, we selected methods that were representative of a diverse panel of algorithms, including diffusion variants, supervised learning on features derived from network propagation, and a number of baseline approaches (see Table 1).

**Table 1:** List of methods included in this benchmark.

Method Identifier	Method Name	Method Class	Implementation	Reference
pr	PageRank with a uniform prior	Baseline	igraph (Bioconductor [24, 25] package)	[26]
random	Random	Baseline	R	(see text)
randomraw	Random Raw	Baseline	R	(see text)
EGAD	Extending Guilt by Association’ by Degree	Baseline	EGAD (Bioconductor package)	[27]
ppr	Personalized PageRank	Diffusion	igraph (R package)	[28]
raw	Raw Diffusion	Diffusion	diffuStats (Bioconductor package)	[29]
gm	GeneMania-based weights	Diffusion	diffuStats (Bioconductor package)	[30]
mc	Monte Carlo normalised scores	Diffusion	diffuStats (Bioconductor package)	[31]
z	Z-scores	Diffusion	diffuStats (Bioconductor package)	[31]
knn	K nearest neighbours	Semi-supervised learning	RANKS (R package)	[32]
wsld	Weighted Sum with Linear Decay	Semi-supervised learning	RANKS (R package)	[32]
COSNet	COst Sensitive neural Network	Supervised learning	COSNet (R package)	[33]
bagsvm	Bagging SVM (based on ProDiGe1)	Supervised learning	kernlab (R package)	[34]
rf	Random Forest	Supervised learning	randomForest (R package) + Matlab (features)	[35]
svm	Support Vector Machine	Supervised learning	kernlab (R package) + Matlab (features)	[35]

Method identifiers are shortened method names used throughout the text. Other columns are self-explanatory.

### Testing framework, algorithms and parameterisation

All tests and batch runs were set-up and conducted using the R statistical programming language [36]. When no R package was available, the methodology was re-implemented, building upon existing R packages whenever possible. Standard R machine learning libraries were used to train the support vector machine and random forest classifiers. Only the MashUp algorithm [35] required feature generation outside of the R environment, using the Matlab code from their publication. Further details on the methods implementation can be found in S1 Appendix, section “Method details”.

EGAD [27], a pure neighbour-voting approach, was used here as a baseline comparator.

Diffusion (propagation) methods are central in this study. We used the random walk-based personalised PageRank [26], previously used in similar tasks [28], as implemented in igraph [37]. The remaining diffusion-based methods were run on top of the regularised Laplacian kernel [38], computed through diffuStats [39]. We included the classical diffusion raw, a weighted approach version gm that assigns a bias term to the unlabelled nodes, and two statistically normalised scores (mc and z), as implemented in diffuStats. The normalised scores adjust for systematic biases in the diffusion scores that relate to the graph topology, in order to provide a more uniform ranking. In the scope of positive-unlabelled learning [40, 41], we included the kernelised scores knn and the linear decayed wsld from RANKS [42]. knn computes each gene score based on the k-nearest positive examples, using the graph kernel to compute the distances. Conversely, wsld uses all the kernel similarities to the positive examples, but applies a decaying factor to downweight the furthest positives. Closing this category, we implemented the bagging Support Vector Machine approach from ProDiGe1 [34], here bagsvm, which trains directly on the graph kernel to find the optimal hyperplane separating positive and negative genes.

Purer ML-based methods were also included. On one hand, network-based features were generated using MashUp [35] and two classical classifiers were fitted to them, based on caret [43] and mlr [44]. These are svm, the Support Vector Machine as implemented in kernlab [45], and rf, the Random Forest found in the randomForest package [46]. On the other hand, we tried the parametric Hopfield recurrent neural network classifier in the COSNet R package [33, 47]. COSNet estimates network parameters on the sub-network containing the labelled nodes, extends them to the sub-network containing the unlabelled ones and then predicts the labels.

Finally, we defined three naive baseline methods: (1) pr, a PageRank with a uniform prior, where input scores on the genes are ignored; (2) randomraw, which applies the raw diffusion approach to randomly permuted input scores on the genes; and (3) random, a uniform re-ranking of input genes without any network propagation. The inclusion of pr and randomraw allowed us to quantify the predictive power of the network topology alone, without any consideration for the input scores on the genes.

### Biological networks

The biological network used in the validation is of critical importance as current network resources contain both false positive and false negative interactions, possibly affecting subsequent predictions [21].

Here, we used two human networks with different general properties, one more likely to contain false positive interactions (STRING [48]), and another more conservative (OmniPath [49]), to test the effect of the network itself on network propagation performance. We further filtered STRING [48] to retain only a subset of interactions. Having tested several filters, we settled upon high-confidence interactions (combined score > 700) with some evidence from the “Experiments” or “Databases” data sources (see Table B in S1 Appendix). Applying these filters and taking the largest connected component resulted in a connected network of 11,748 nodes and 236,963 edges. Edges were assigned weights between 0 and 1 by rescaling the STRING combined score.

We did not filter the OmniPath network [49]. After removing duplicated edges and taking the largest connected component, the OmniPath network contained 8,580 nodes and 42,145 unweighted edges.

### Disease gene data

We used the Open Targets platform [19] to select known disease-related genes. In this analysis we defined positive genes as those reported in Open Targets as being the target of any known drug against the disease of interest, from which all the metrics were computed. We decided to use drug targets, including unsuccessful ones, as proxies for disease genes on the basis that genes for which a drug programme has been started, generally with significant investment, are most likely to have strong evidence linking them to the disease. We therefore regard them as a set of high-confidence true positive disease genes. This choice means we potentially miss genes that have strong genetic associations to the disease but are not druggable. In other words, we focus on limiting false positives in our reference set of positives, at the expense of having more false negatives in our set of negatives. Alternatively, genes with a genetic association of sufficient confidence with the disease were also used as an input data stream, in order to assess the predictive power of an indirect source of evidence. Associations were binarised: any non-zero drugs-related association was considered positive, implying that the methods would predict genes on which a drug has been essayed, regardless of whether the drug was eventually approved. Likewise, only genetic associations with an Open Targets score above 0.16 (see Figure A in S1 Appendix) were considered positive. We considered exclusively common diseases with at least 1,000 Open Targets associations, of which a minimum of 50 could be based on known drugs and 50 on genetic associations, in order to avoid empty folds in the nested cross-validations. By applying these filters, we generated a list of phenotypes and diseases which we then manually curated to remove, non-disease phenotype terms (e.g. “body weight and measures”) as well as vague or broad terms (e.g.“cerebrovascular disorder” or “head disease”) and infectious diseases. We also decided to exclude cancers from this analysis. Cancer is a complex process starting from the driver mutation(s) causing disruptive processes involving clonal expansions, which are known to carry their own specific and resultant (non-causal) passenger mutations. Also, the fundamental genetic and biological mechanisms underlying cancers [50] are generally very distinct from other diseases. We considered this might affect the reliability of the seed genes and cancers would therefore deserve a separate benchmark. This left 22 diseases considered in this study (Table 2). Further descriptive material on the role of genes associated with disease within the STRING network can be found in the section “Descriptive disease statistics in the STRING network” from S1 Appendix.

**Table 2:** List of diseases included in this study.

Disease	N(genetic)	N(drugs)	Overlap	P-value	FDR
allergy	112	57	1	4.22e-01	4.42e-01
Alzheimers disease	208	103	4	1.10e-01	1.42e-01
arthritis	174	188	6	6.08e-02	1.03e-01
asthma	105	80	6	7.77e-05	5.70e-04
bipolar disorder	117	148	3	1.83e-01	2.12e-01
cardiac arrhythmia	75	177	6	9.15e-04	3.36e-03
chronic obstructive pulmonary disease (COPD)	154	116	6	4.18e-03	1.31e-02
coronary heart disease	111	171	4	7.86e-02	1.24e-01
drug dependence	75	143	6	2.96e-04	1.30e-03
hypertension	66	188	2	2.85e-01	3.14e-01
multiple sclerosis	71	167	4	1.83e-02	4.03e-02
obesity	69	194	3	1.06e-01	1.42e-01
Parkinson’s disease	55	145	0	1	1
psoriasis	131	105	7	1.68e-04	9.23e-04
rheumatoid arthritis	138	95	5	5.18e-03	1.42e-02
schizophrenia	410	163	17	5.44e-05	5.70e-04
stroke	90	156	3	1.18e-01	1.44e-01
systemic lupus erythematosus (lupus)	126	109	5	6.30e-03	1.54e-02
type I diabetes mellitus	87	106	3	4.39e-02	8.04e-02
type II diabetes mellitus	130	154	4	9.14e-02	1.34e-01
ulcerative colitis	136	51	7	1.81e-06	3.98e-05
unipolar depression	123	121	4	3.81e-02	7.63e-02

Diseases included in this study, with a minimum of 50 associated genes both in the known drug targets and the genetic categories (see text). The overlap between these two lists of genes showed a degree of dependence between these two Open Targets data streams for some of the diseases. P-values were calculated using Fisher’s exact test and are reported without and with correction for false discovery rate [51].

### Validation strategies

#### Input gene scores

**Fig 8:**
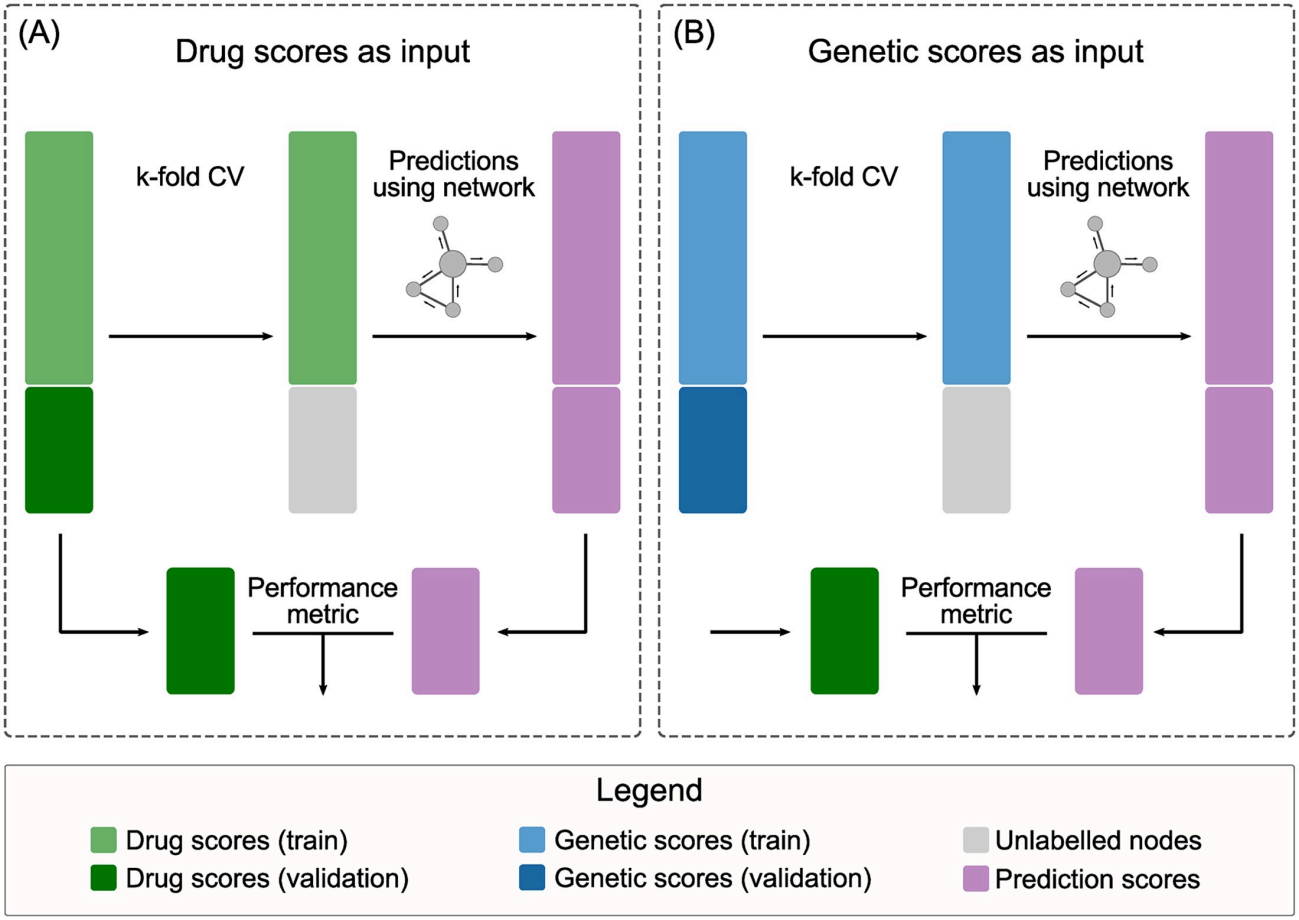
Input gene scores. Two input types were used to feed the prioritisation algorithms: the binary drug scores in panel (A) and the binary genetic scores in panel (B). In both cases, the validation genes were deemed unlabelled in the input to the prioritisers. Cross-validation folds were always calculated taking into account the drugs input and reused on the genetic input.

We used the binarised drug association scores and genetic association scores from Open Targets as input gene-level scores to seed the network propagation analyses (Fig 8) and test their ability to recover known drug targets. With the first approach (panel (A) in Fig 8), we tested the predictive power of current network propagation methods for drug target identification using a direct source of evidence (known drug targets). In the second approach (panel (B) in Fig 8), we assessed the ability of a reasonable but indirect source of evidence – genetic associations to disease – in combination with network propagation to recover known drug targets.

#### Metrics

Methods were systematically compared using standard performance metrics. The Area under the Receiver Operating Characteristic curve (AUROC) is extensively used in the literature for binary classification of disease genes [52], but can be misleading in this context given the extent of the class imbalance between target and non-target genes [53]. We however included it in our benchmark for comparison with previous literature. More suitable measures of success in this case are Area under the Precision-Recall curve (AUPRC) [53] and partial AUROC (pAUROC) [54].

Based on the notation in [54–56], let *Z* be a real-valued random variable corresponding to the output of a given prioritiser, so that largest values correspond to top ranked genes. Let *X* and *Y* be the outputs for negative and positive genes, i.e. *Z* is a mixture of *X* and *Y*, representing by *D* the indicator variable (*D* = 0 for negatives and *D* = 1 for positives). For an arbitrary threshold *c*, the following metrics can be defined: true positive rate *TPR*(*c*) = *P*(*Y* > *c*) = *P*(*Z* > *c* | *D* = 1), false positive rate *FPR*(*c*) = *P*(*X* > *c*) = *P*(*Z* > *c* | *D* = 0), precision *Prec*(*c*) = *P*(*D* = 1 | *Z* > *c*) and recall *Recall*(*c*) = *P*(*Y* > *c*). Then:

$$\begin{array}{c} \text{AUROC}=\int_{c=\infty }^{-\infty }TPR\left(c\right)\;dFPR\left(c\right) \end{array}$$ (1)

$$\begin{array}{c} \text{pAUROC}\left(p\right)=\frac{1}{p}\int_{c=\infty }^{c_{p}}TPR\left(c\right)\;dFPR\left(c\right)\;\text{where}\;FPR\left(c_{p}\right)=p\in \left(0,1\right) \end{array}$$ (2)

$$\begin{array}{c} \text{AUPRC}=\int_{c=\infty }^{-\infty }Prec\left(c\right)\;dRecall\left(c\right) \end{array}$$ (3)

Note that pAUROC contains a normalising constant $\frac{1}{p}$ because the partial area is bounded between 0 and *p*; the constant allows the metric to lie in [0, 1] again. AUROC, AUPRC and pAUROC were computed with the precrec R package [57]. We also included top *k* hits, defined as the number of true positives in the top *k* predicted genes (proportional to precision at *k*). Given the output of a prioritiser on *n* genes, *z*_1_ ≥ *z*_2_ ≥ *z*_3_ ≥ … ≥ *z*_*n*_:

$$\begin{array}{c} \text{top}\left(k\right)=\sum_{i=z_{1}}^{z_{k}}D_{i} \end{array}$$ (4)

It is straightforward, intuitive and most likely to be useful in practice, such as a screening experiment where only a small number of predicted hits can be assayed.

The main body focuses on AUROC, AUPRC and top 20 hits. We considered another 3 metrics, reported only in S1 Appendix: partial AUROC up to 5% FPR, partial AUROC up to 10% FPR, and number of hits within the top 100 genes.

#### Cross-validation schemes and protein complexes

Standard (stratified) and modified k-fold cross-validation were used to estimate the performance of the methods. Folds were based upon known drugs-related genes, regardless of which type of input was used (see Fig 8). Genes in the training fold were negatively or positively labelled according to the input type, whereas genes in the validation fold were left unlabelled.

The direct application of cross-validation to this problem posed a challenge: known drug targets often consist of protein complexes, e.g. multi-protein receptors. Drug-target associations typically have complex-level resolution. The drug target data from Open Targets comes from ChEmbl [58], in which all the proteins in the targeted complex are labelled as targets.

**Fig 9:**
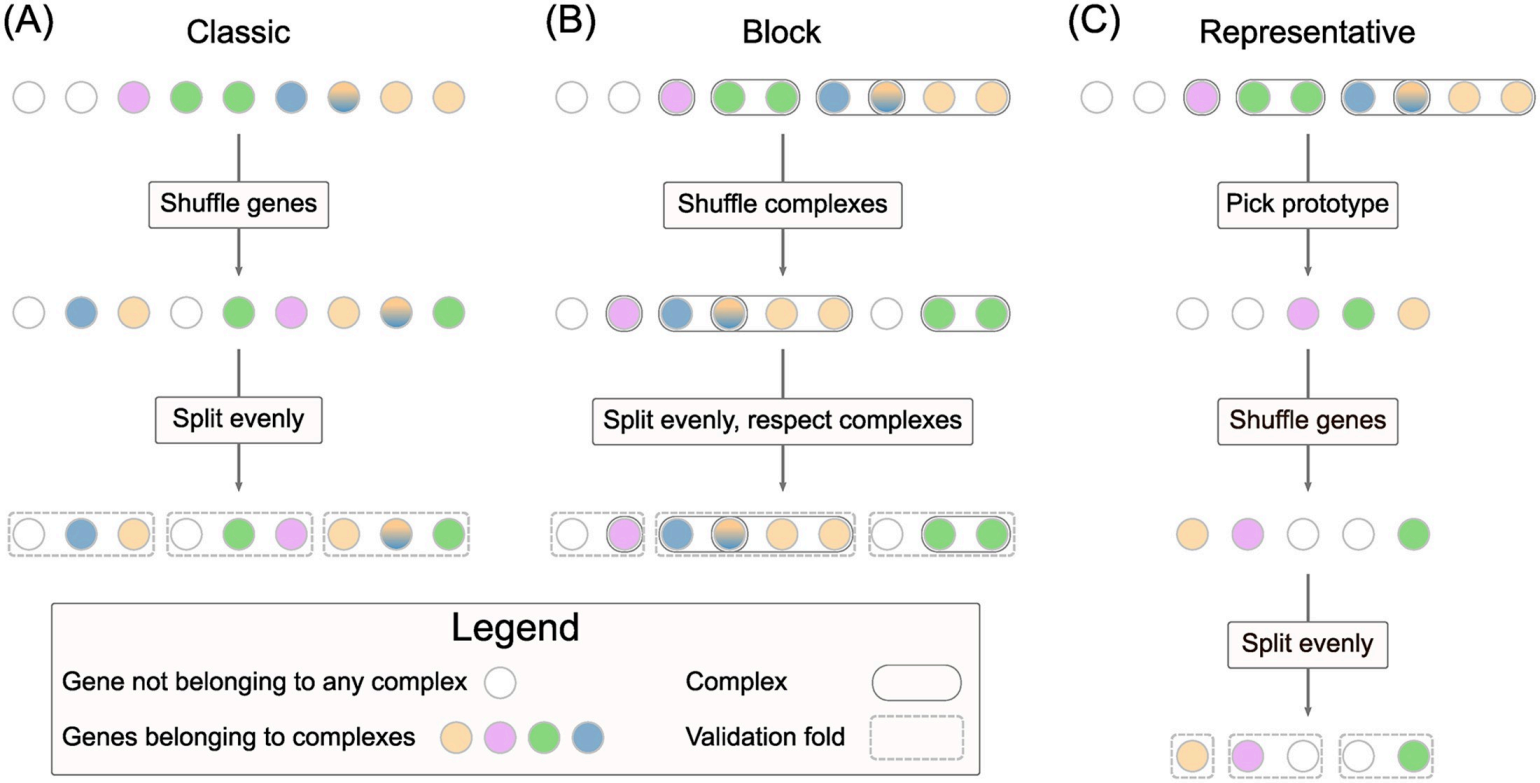
Cross-validation schemes. Three cross-validation schemes were tested. **(A)**: standard k-fold stratified cross-validation that ignored the complex structure. **(B)**: block k-fold cross-validation. Overlapping complexes were merged and the resulting complexes were shuffled. The folds were computed as evenly as possible without breaking any complex. **(C)**: representative k-fold cross-validation. Overlapping complexes were merged and the resulting complexes from which unique representatives were chosen uniformly at random. Then a standard k-fold cross-validation was run on the representatives, but excluding the non-representatives from train and validation.

If left uncorrected, this could bias cross-validation results: networks densely connect proteins within a complex, random folds would frequently split positively labelled complexes between train and validation, and therefore network propagation methods would have an unfair advantage at finding positives in the training folds. In view of this, we benchmarked the methods under three cross-validation strategies: a standard cross-validation (A) in line with usual practice and two (B, C) complex-aware schemes (Fig 9) addressing non-independence between folds when the known drug targets act as input.

Strategy (A), called classic, was a regular stratified *k*-fold repeated cross-validation. We used *k* = 3 folds, averaging metrics over each set of folds, repeated 25 times (see also Fig 1).

Strategy (B), named block, performed a repeated cross-validation while explicitly preventing any complexes that contain disease genes to be split across folds. The key point is that, where involved, shuffling was performed at the complex level instead of the gene level – overlapping complexes that shared at least one known drug target were merged into a larger pseudo-complex before shuffling. Fold boundaries were chosen so that no complex was divided into two folds, while keeping them as close as possible to those that would give a balanced partition, see Fig 9. Nevertheless, a limitation of this scheme is that it can fail to balance fold sizes in the presence of large complexes (see Figure I in S1 Appendix). For example, chronic obstructive pulmonary disease exhibited imbalanced folds, as 50 of the proteins involved belong to the Mitochondrial Complex I.

Strategy (C), referred to as representative, selected only a single representative or prototype gene for each complex to ensure that gene information in a complex was not mixed between training and validation folds. In each repetition of cross-validation, after merging the overlapping complexes, a single gene from each complex was chosen uniformly at random and kept as positive. The remaining genes from the complexes involved in the disease were set aside from the training and validation sets, in order (1) not to mislead methods into assuming their labels were negative in the training phase, and (2) not to overestimate (if set as positives) or penalise (if set as negatives) methods that ranked them highly, as they were expected to do so. This strategy kept the folds balanced, but at the expense of a possible loss of information by summarising each complex by a single gene at a time, reducing the number of positives for training and validation.

### Additive performance models

For a systematic comparison between diseases, methods, cross-validation schemes and input types, we fitted an additive, explanatory regression model to the performance metrics of each (averaged) fold from the cross-validation. The use of main effect models eased the evaluation of each individual factor while correcting for the other covariates. We modelled each metric *f* separately for each input type, not to mix problems of different nature:

$$\begin{array}{c} f∼\text{cv}\_\text{scheme}+\text{network}+\text{method}+\text{disease} \end{array}$$ (5)

We fitted dispersion-adjusted logistic-like *quasibinomial* variance models for the metrics AUROC, pAUROC and AUPRC and *quasipoisson* for top *k* hits. The quasi-likelihood formalism protected against over and under-dispersion issues, in which the observed variance is either higher or lower than that of the theorical fitted distribution [59], affecting subsequent statistical tests. *The effect of changing any of the four main effects is discussed in separate sub-sections in Results, following the order from the formula above*. After a data-driven choice of cross-validation scheme and network, we fitted reduced explanatory models within them for a more accurate description:

$$\begin{array}{c} f∼\text{method}+\text{disease} \end{array}$$ (6)

### Qualitative methods comparison

The rankings produced by the different algorithms were qualitatively compared using Spearman’s footrule [60]. Distances were computed between all method ranking pairs for each individual combination of disease, input type, network and for the top *N* predicted genes, excluding the original seed genes. This part does not involve cross-validation – all known disease-associated genes were used for gene prioritisations. Pairs of rankings could include genes uniquely ranked highly by a single algorithm from the comparison, so mismatch counts (i.e. percentage mismatches) between these rankings were also taken into account. Mismatches occur when a gene features in the top *N* predictions of one algorithm and is missing from the corresponding ranking by another algorithm. A compact visualisation of distance matrices was obtained using a multi-view extension of MDS [61–63]. For this we used the R package *multiview* [64] that generates a single, low-dimensional projection of combined inputs (disease, input and network).

## Supporting information

S1 Appendix Supplement. This document contains complementary material that supports our claims in the main body. It includes topics such as descriptive statistics, topological properties of disease-associated genes, raw metrics plots, method details, MDS plots, alternative performance metrics and further explanatory models.
(PDF)

S1 File MDS plots. Complementary single-disease MDS plots and distance matrices.
(ZIP)

S2 File Interactions HTML viewer. Stand-alone viewer to explore models with interaction terms.
(ZIP)
