# Supplementary material for: Benchmarking network propagation methods for disease gene identification
Source: PLoS Comput Biol. 2019 Sep 3;15(9):e1007276. doi: 10.1371/journal.pcbi.1007276 (PMC6743778; doi:10.1371/journal.pcbi.1007276)
Supplement: S1 Appendix — This document contains complementary material that supports our claims in the main body. It includes topics such as descriptive statistics, topological properties of disease-associated genes, raw metrics plots, method details, MDS plots, alternative performance metrics and further explanatory models. (PDF) [file pcbi.1007276.s001.pdf]

Appendix: Benchmarking network propagation methods for disease gene identification

Sergio Picart-Armada, Steven J. Barrett, David R. Willé,
Alexandre Perera-Lluna, Alex Gutteridge, Benoit H. Dessailly

Descriptive statistics

OpenTargets data streams

File `17.06_association_data.json` with gene-disease associations from June 2017 was downloaded from the Open Targets data download page. The original table consists of 187,246 rows and 7 data streams, encompassing associations between 90 diseases and genes with evidence on one or more streams. We selected those diseases that had at least 50 drug-associated and genetically-associated genes, resulting in a final list of 22 common diseases as shown in the main body.

Table A: Descriptive statistics on the seven OpenTargets data streams and the overall score. Included are the binarised scores (genetic and drugs) used for the benchmark.

Statistic	N	Mean	St. Dev.	Min	Pctl(25)	Median	Pctl(75)	Max
association_score.overall	187,246	0.079	0.160	0.0004	0.016	0.032	0.064	1.000
association_score.datatypes.genetic_association	187,246	0.015	0.082	0.000	0.000	0.000	0.000	1.000
association_score.datatypes.somatic_mutation	187,246	0.0004	0.015	0.000	0.000	0.000	0.000	1.000
association_score.datatypes.known_drug	187,246	0.023	0.140	0.000	0.000	0.000	0.000	1.000
association_score.datatypes.affected_pathway	187,246	0.0003	0.017	0	0	0	0	1
association_score.datatypes.rna_expression	187,246	0.015	0.037	0.000	0.000	0.000	0.015	0.651
association_score.datatypes.literature	187,246	0.021	0.030	0.000	0.000	0.014	0.030	0.321
association_score.datatypes.animal_model	187,246	0.008	0.036	0.000	0.000	0.000	0.000	0.313
known_drug_binary	187,246	0.035	0.184	0	0	0	0	1
known_gene_binary	187,246	0.032	0.176	0	0	0	0	1

Networks from the STRING database

STRING data: version 10, species 9606, score threshold 400. STRING uses the ENSEMBL protein identifiers [ZAA+18], so the OpenTargets associations were mapped from ENSEMBL gene to ENSEMBL protein through the *map()* function from the STRINGdb package [48]. No collisions (i.e. two genes mapping to the same protein) were encountered.

Table B: Summary of the STRING networks with several filtering options; edges that meet the filtering condition are dropped. Described are the number of nodes, edges, rows from the disease table whose protein maps to the network (originally, 187,246 rows) and coverage of the binarised drugs and genetic scores. Numbers referring to the largest connected component are outside the parentheses, while the original amount is detailed inside them. The filters apply only to the edges, therefore all the networks have the same order (18884), mapped rows and mapped genes before taking the largest connected component.

<table>
<tr><th>network</th><th>filter</th><th>nodes</th><th>edges</th><th>coverage_allrows</th><th>coverage_drug</th><th>coverage_genetic</th></tr>
<tr><td>Net1</td><td>combined_score < 400 | experiments < 1</td><td>13307(18884)</td><td>103607(103648)</td><td>153747(178622)</td><td>5687(6395)</td><td>4593(5751)</td></tr>
<tr><td>Net2</td><td>experiments < 600</td><td>8854(18884)</td><td>37084(37288)</td><td>109535(178622)</td><td>3791(6395)</td><td>3115(5751)</td></tr>
<tr><td>Net3</td><td>experiments < 400 & database < 400</td><td>14149(18884)</td><td>284759(284786)</td><td>159554(178622)</td><td>6190(6395)</td><td>4750(5751)</td></tr>
<tr><td>Net4</td><td>combined_score < 700 | (experiments < 1 & database < 1)</td><td>11748(18884)</td><td>236963(237049)</td><td>144920(178622)</td><td>6121(6395)</td><td>4170(5751)</td></tr>
<tr><td>Net5</td><td>combined_score < 700 | (experiments < 1 & database < 1 & textmining < 900)</td><td>12022(18884)</td><td>240082(240193)</td><td>147635(178622)</td><td>6160(6395)</td><td>4266(5751)</td></tr>
<tr><td>Net6</td><td>database < 400</td><td>7564(18884)</td><td>205866(206177)</td><td>110293(178622)</td><td>5711(6395)</td><td>2881(5751)</td></tr>
<tr><td>All</td><td>combined_score < 0</td><td>18884(18884)</td><td>740950(740950)</td><td>178622(178622)</td><td>6395(6395)</td><td>5751(5751)</td></tr>
</table>

We choose Net4 as our main network, as it provides a good balance between mapping and coverage. The edge weights were obtained by rescaling the STRING combined score to lie in $[0, 1]$. For the MashUp network-based feature generation, the experimental and the database STRING-based networks before combined through their algorithm, instead of using the combined weight provided by STRING.

The (unweighted) shortest path distribution of the final STRING network is depicted in figure B:

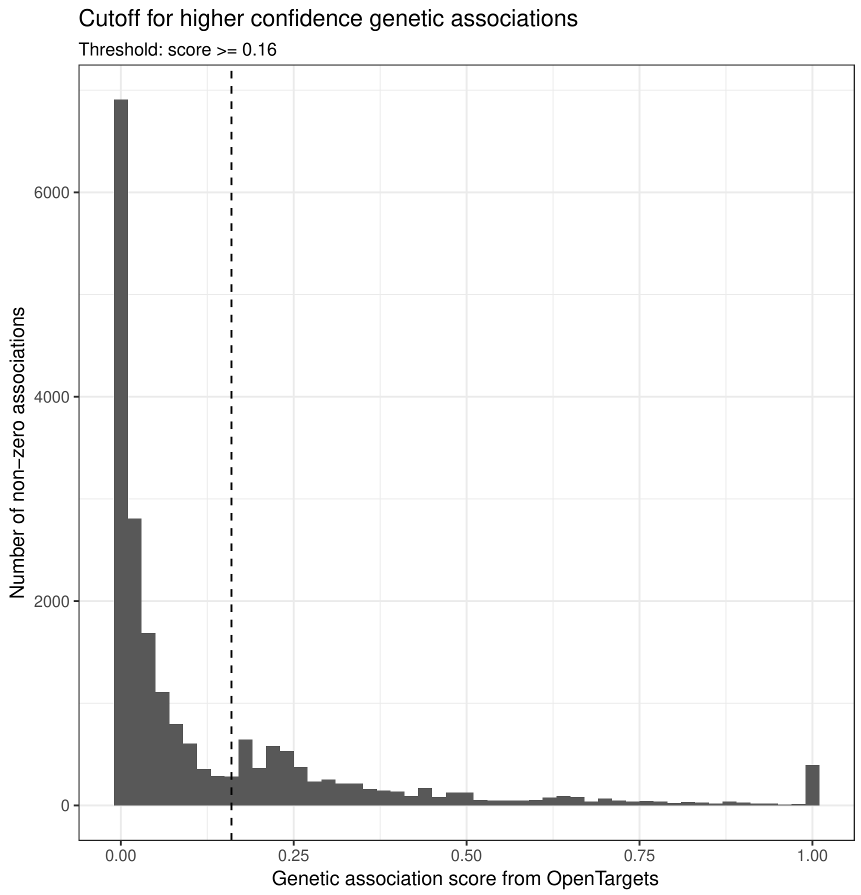

Figure A: Histogram of genetic associations. The proposed threshold of 0.16 separates lower and higher quality genetic associations and is therefore used to binarise this data stream.

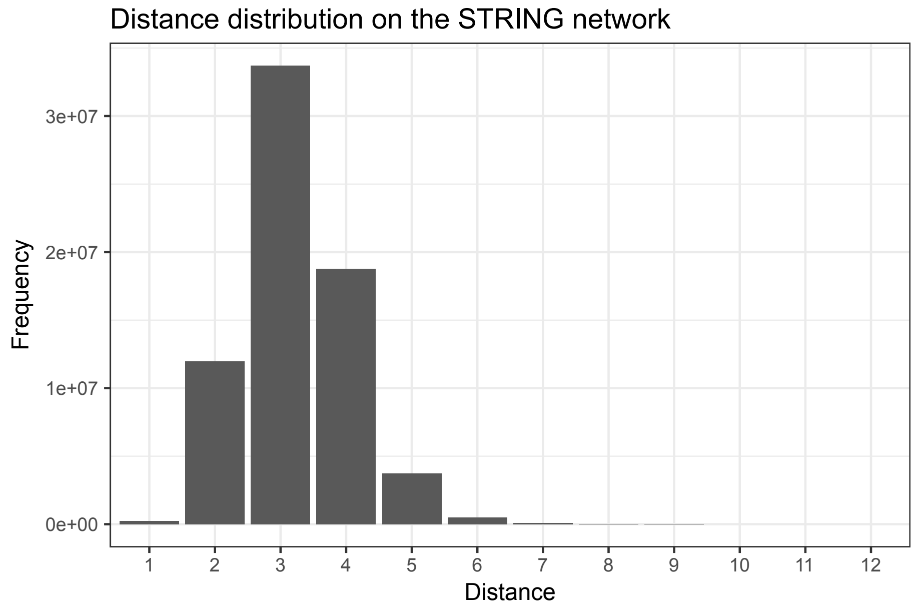

Figure B: Distance distribution in the STRING network, computed for every pair of nodes. Most of the nodes lie within a distance of 5 or less, suggesting the presence of biological hubs.

The OmniPath network

The original OmniPath file contained a total of 8,951 nodes and 50,247 edges. Removing duplicated edges and keeping the largest component left a network with 8,580 nodes and 42,145 edges. The proteins are represented by their UniProt identifier [The17] and later mapped to ENSEMBL protein [ZAA^{+}18]. After mapping, 62 genes

mapped to a non-unique protein. For these proteins with multiple gene annotations, we chose:

1. The gene with a known drug target
2. In case of tie(s), the gene with a known genetic association
3. In case of tie(s), the gene with the highest overall association score
4. In case of tie(s), pick a random gene

From the original 187,246 rows in the disease table, 125,007 mapped to the OmniPath network, encompassing a total of 5,084 drugs-related genes and 3,442 genetics-related genes.

Descriptive disease statistics in the STRING network

After mapping the (drugs-related) disease genes to the main STRING network, we observe that every pair of diseases shows overlap. This can range from a modest amount (less than 10 genes) to more than 100 genes. Examples of the latter include type II diabetes, coronary heart disease, obesity, hypertension and bipolar disorder, all of which share a notable background.

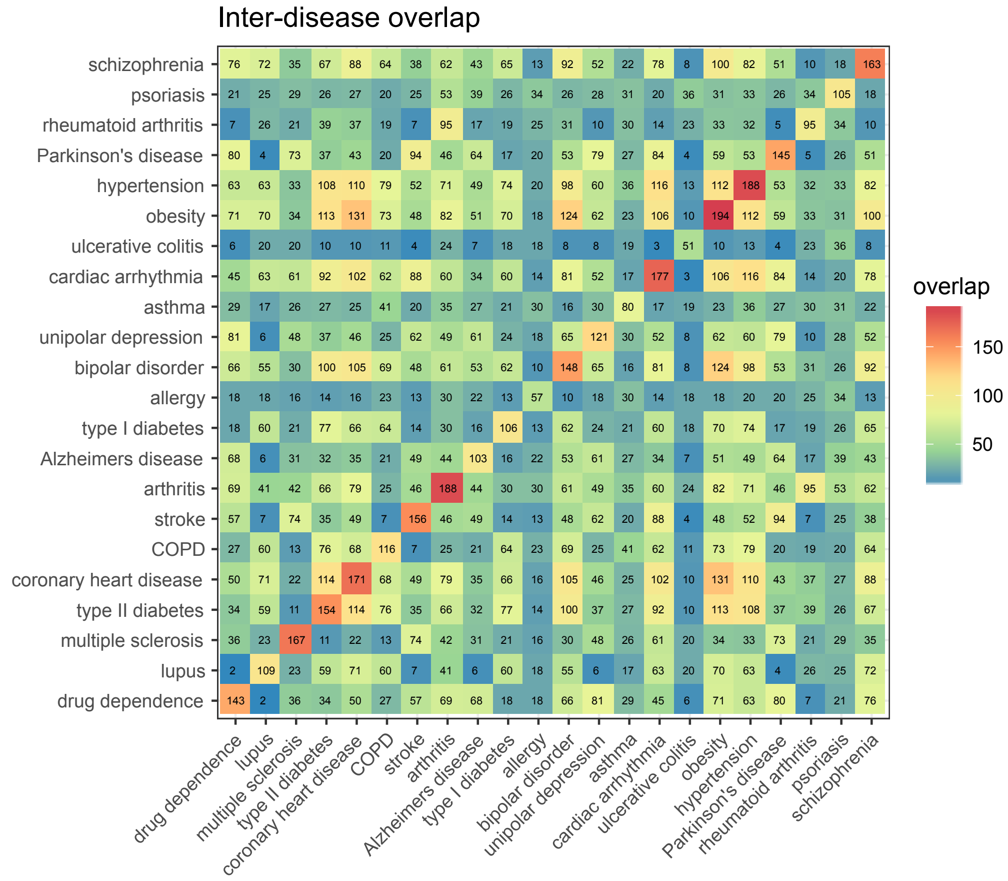

Figure C: Disease overlap after mapping the genes to the STRING network. There are no disjoint diseases.

In turn, this suggests that some genes might participate in multiple diseases, which is confirmed in figure D. Several genes participate in 10 or more diseases (out of 22), thus creating an overlap between any pair of them.

The fact that all diseases share at least one gene implies that their distance within the network is always 0. However, we can examine the mean distance between two diseases, defined as the mean of the distance of every pair of genes (g_i, g_j) with g_i belonging to the first disease and g_j to the second. If we group the rows and columns using the UPGMA algorithm [GM07], taking into account that, at the starting point, every disease is in practice equivalent to a cluster of genes.

Each disease, in turn, tends to form a module within the network. To show this, we have computed the modularity of each disease, as implemented in the `modularity` function from the igraph R package [37]. A modularity greater than zero indicates that the number of connections within the disease genes is greater than

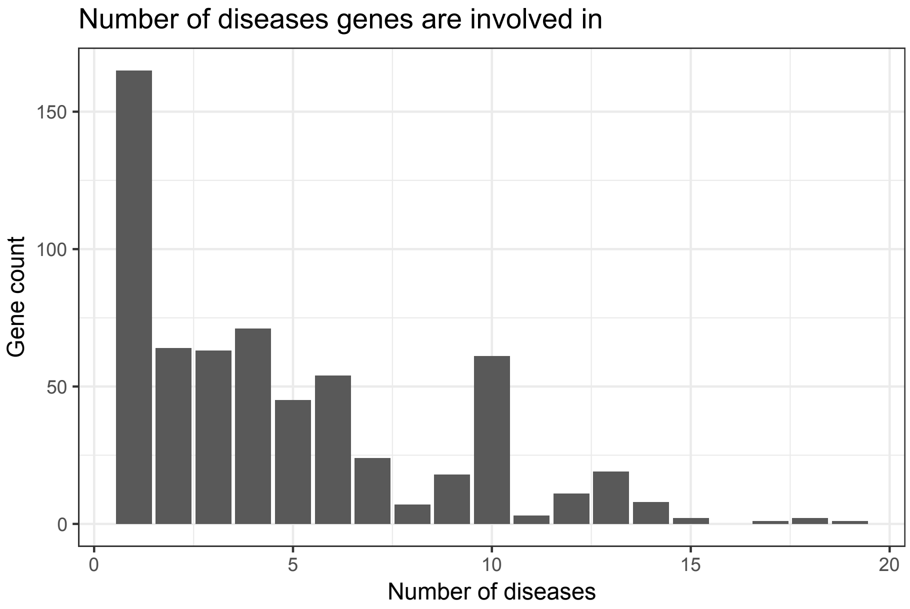

Figure D: Histogram of the number of diseases genes participate in. The majority of genes belong to a single disease, but a small part of genes are found in 10 or more diseases, unveiling a common core in drug targets.

that of a randomly rewired network. Figure F shows how all diseases deviate from their randomised gene sets, which is something expected. Diseases with higher modularity can be easier to predict: an example is cardiac arrhythmia, very modular and well predicted, see the additive models on drugs data.

Another way to examine how close disease genes lie is by representing the mean distance to the disease genes, starting either (1) from the disease genes or (2) from the rest of genes (i.e. non-disease genes for this particular disease). Figure G shows how drugs-related genes from a given disease have a shorter mean distance to themselves than the rest of genes in the network.

Finally, we observe that drugs-related disease genes have larger centrality measures than the rest of nodes in the network (figure H). This supports the hypothesis that the centrality itself has predictive power, hereby examined by including the PageRank centrality measure as a baseline method.

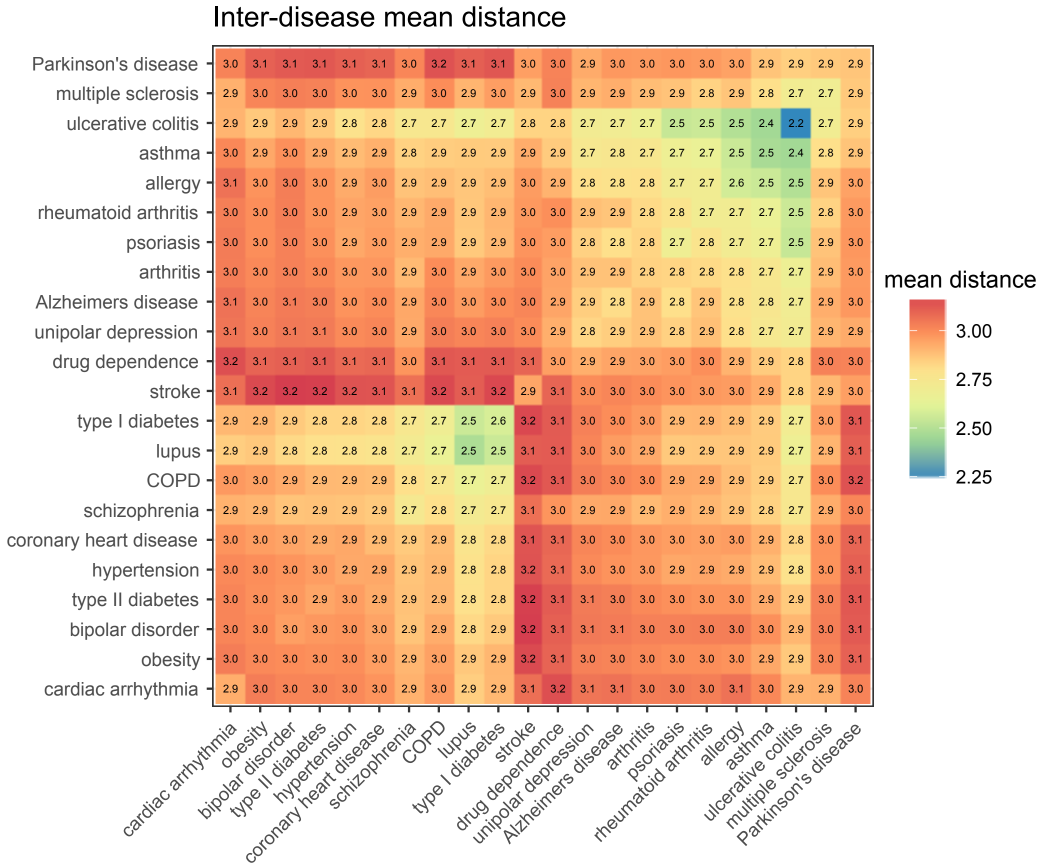

Figure E: Mean distance between diseases on the STRING network, grouping rows and columns by UPGMA. The lower-left block is coincident with the diseases having a high overlap, as shown in figure C.

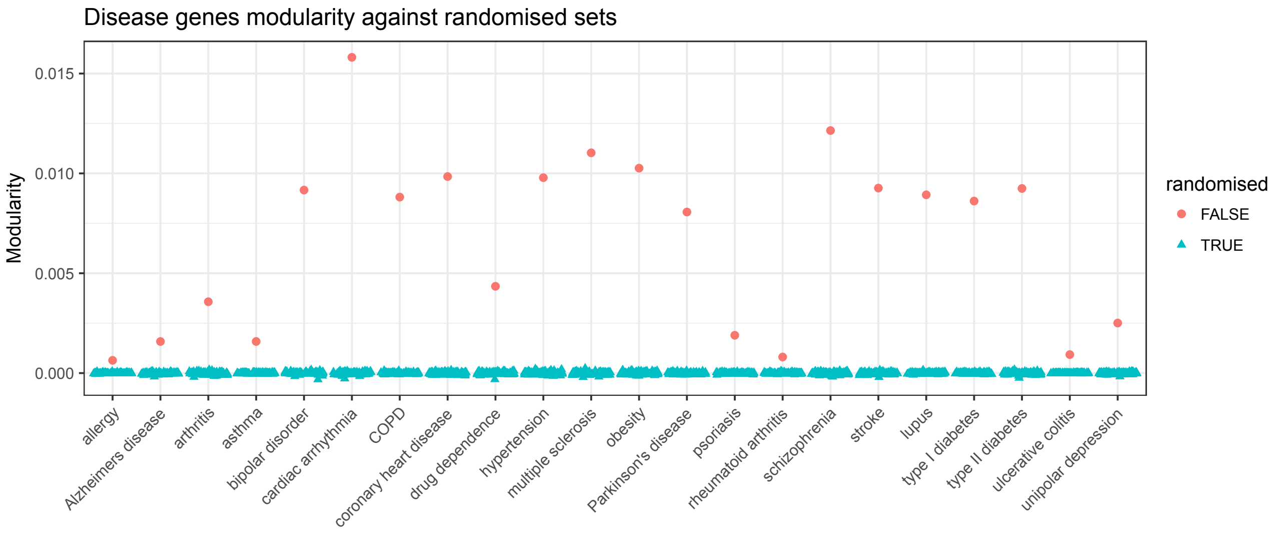

Figure F: Modularity by disease, compared to randomised trials of the same number of genes. First, we have computed the modularity of the drugs-related disease genes for a given disease, represented through a red dot. Then, we have sampled the same number of genes uniformly from the network, a total of 100 times per disease, and computed their modularity (blue triangles). Random trials lie close to 0, due to the definition of modularity itself, and actual diseases show positive values.

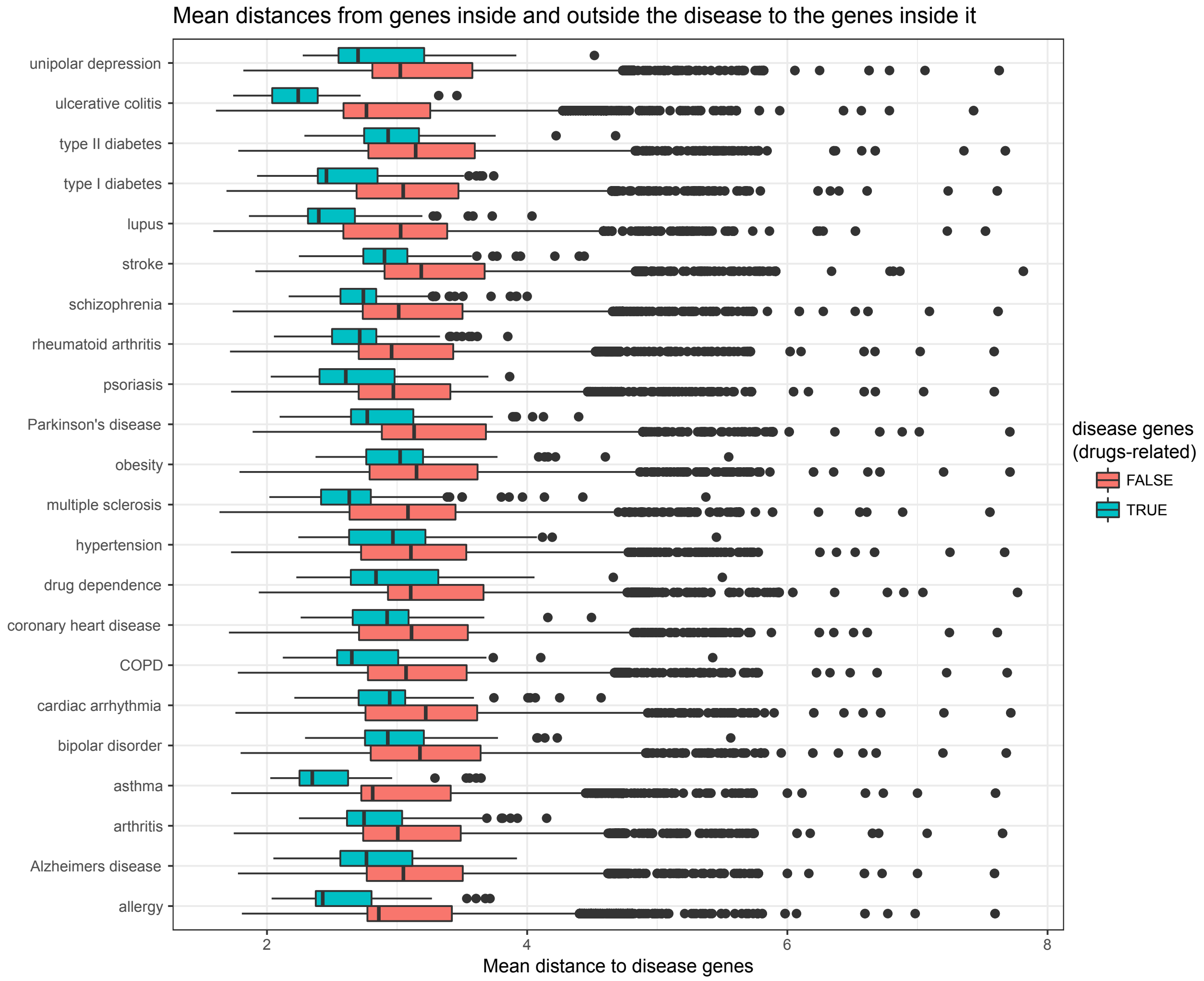

Figure G: Mean distances to the drugs-related disease genes, computed either from the disease genes (average distance from a disease gene to all the disease genes) or from the non-disease genes (average distance from a non-disease gene to all the disease genes).

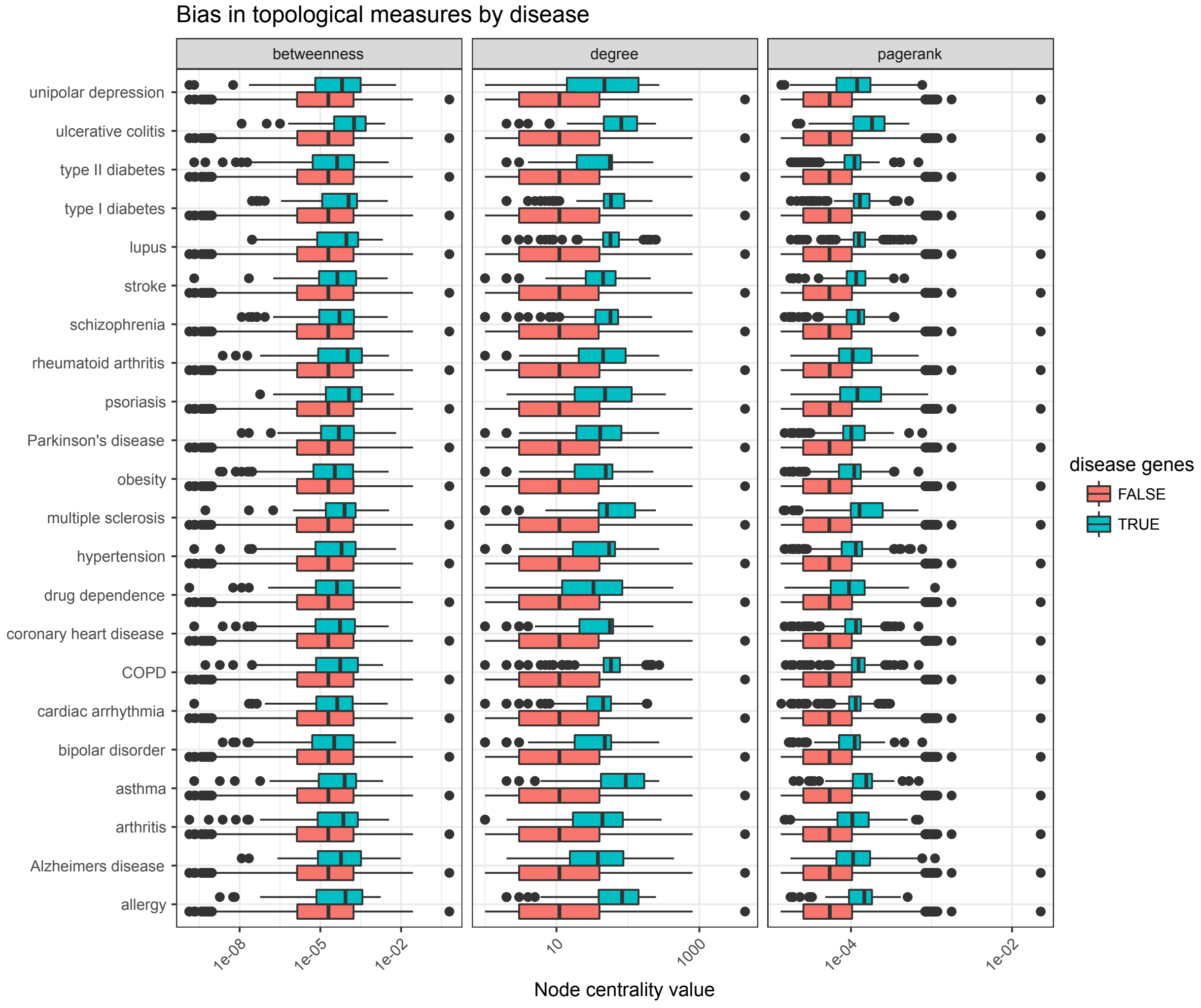

Figure H: Comparison of the centrality of disease and non-disease genes. We have computed three centrality measures for all the genes in the network: the node degree, the PageRank (as implemented in `page.rank`, with uniform prior and default damping factor $d = 0.85$) in the R package igraph [37], and the node betweenness, also implemented in the `betweenness` function in igraph. Note that centrality measures are a topological property and do not use disease data as input. For each disease, all the genes have been separated into drugs-related disease genes (blue) and non-disease (red). We can appreciate how, consistently along the three metrics, drugs-related genes tend to have higher centralities.

Complex data

Chembl complex data was retrieved from `https://www.ebi.ac.uk/chembl/downloads`, specifically release 23 (doi `10.6019/CHEMBL.database.23`). The original data comprises 214 complexes with a mean of 9.29 proteins in each and a standard error of 14.08. Having mapped the complexes to the STRING network, 207 non-empty complexes remain, with a mean of 3.251 ENSEMBL ids in each and a standard error of 4.728.

Table C: Summary statistics of the size (in proteins per complex) of the 214 complexes before and after mapping to the STRING network. Complexes that fail to map have been dropped, hence the differences in their total number N.

Statistic	N	Mean	St. Dev.	Min	Pctl(25)	Median	Pctl(75)	Max
original	214	9.290	14.080	2	4	6	9	120
mapped to STRING	207	3.251	4.728	1	2	2	3	47
mapped to OmniPath	206	2.981	3.828	1	2	2	3	37

Cross validation splits

Table D: Number of folds computed for the cross validation in the STRING network. Block cross validation contains slightly less folds because invalid folds have been discarded.

cv_scheme	count
classic	1650
block	1647
representative	1650

Table E: Summary statistics on the cross validation folds on drugs input. Specifically, on (1) the number of positives in the training fold, (2) positives in the validation fold and (3) number of split complexes. The mean values are outside the parentheses, which contain its standard deviation. We can observe how classic cross validation splits complexes, but none of the complex-aware strategies do. Also, block cross validation can lead to data imbalance, contrary to classic and representative schemes.

	train_pos			validation_pos			split_complexes		
disease	classic	block	representative	classic	block	representative	classic	block	representative
allergy	38.0(0.00)	38.0(1.42)	32.0(0.00)	19.0(0.00)	19.0(1.42)	16.0(0.00)	5.2(1.73)	0.0(0.00)	0.0(0.00)
Alzheimers disease	68.7(0.47)	68.7(4.79)	48.0(0.00)	34.3(0.47)	34.3(4.79)	24.0(0.00)	19.0(3.79)	0.0(0.00)	0.0(0.00)
arthritis	125.3(0.47)	125.3(4.91)	81.3(0.47)	62.7(0.47)	62.7(4.91)	40.7(0.47)	20.5(4.05)	0.0(0.00)	0.0(0.00)
asthma	53.3(0.47)	53.3(1.22)	48.7(0.47)	26.7(0.47)	26.7(1.22)	24.3(0.47)	6.3(3.23)	0.0(0.00)	0.0(0.00)
bipolar disorder	98.7(0.47)	98.7(20.02)	50.0(0.00)	49.3(0.47)	49.3(20.02)	25.0(0.00)	16.8(3.34)	0.0(0.00)	0.0(0.00)
cardiac arrhythmia	118.0(0.00)	118.0(22.54)	59.3(0.47)	59.0(0.00)	59.0(22.54)	29.7(0.47)	17.6(3.68)	0.0(0.00)	0.0(0.00)
COPD	77.3(0.47)	77.3(21.00)	44.7(0.47)	38.7(0.47)	38.7(21.00)	22.3(0.47)	6.9(3.34)	0.0(0.00)	0.0(0.00)
coronary heart disease	114.0(0.00)	114.0(19.94)	57.3(0.47)	57.0(0.00)	57.0(19.94)	28.7(0.47)	19.8(3.35)	0.0(0.00)	0.0(0.00)
drug dependence	95.3(0.47)	95.3(10.61)	58.7(0.47)	47.7(0.47)	47.7(10.61)	29.3(0.47)	24.8(4.46)	0.0(0.00)	0.0(0.00)
hypertension	125.3(0.47)	125.3(18.51)	70.7(0.47)	62.7(0.47)	62.7(18.51)	35.3(0.47)	24.7(4.82)	0.0(0.00)	0.0(0.00)
multiple sclerosis	111.3(0.47)	111.3(9.62)	74.0(0.00)	55.7(0.47)	55.7(9.62)	37.0(0.00)	11.5(2.00)	0.0(0.00)	0.0(0.00)
obesity	129.3(0.47)	129.3(16.64)	69.3(0.47)	64.7(0.47)	64.7(16.64)	34.7(0.47)	20.3(3.80)	0.0(0.00)	0.0(0.00)
Parkinson's disease	96.7(0.47)	96.7(3.73)	77.3(0.47)	48.3(0.47)	48.3(3.73)	38.7(0.47)	17.3(3.91)	0.0(0.00)	0.0(0.00)
psoriasis	70.0(0.00)	70.0(3.18)	53.3(0.47)	35.0(0.00)	35.0(3.18)	26.7(0.47)	17.6(3.87)	0.0(0.00)	0.0(0.00)
rheumatoid arthritis	63.3(0.47)	63.3(2.30)	51.3(0.47)	31.7(0.47)	31.7(2.30)	25.7(0.47)	7.3(2.29)	0.0(0.00)	0.0(0.00)
schizophrenia	108.7(0.47)	108.7(20.06)	46.7(0.47)	54.3(0.47)	54.3(20.06)	23.3(0.47)	9.1(1.94)	0.0(0.00)	0.0(0.00)
stroke	104.0(0.00)	104.0(8.53)	66.0(0.00)	52.0(0.00)	52.0(8.53)	33.0(0.00)	18.1(3.23)	0.0(0.00)	0.0(0.00)
lupus	72.7(0.47)	71.7(22.34)	30.7(0.47)	36.3(0.47)	37.3(22.34)	15.3(0.47)	5.8(1.71)	0.0(0.00)	0.0(0.00)
type I diabetes mellitus	70.7(0.47)	70.2(22.46)	32.7(0.47)	35.3(0.47)	35.8(22.46)	16.3(0.47)	5.7(1.78)	0.0(0.00)	0.0(0.00)
type II diabetes mellitus	102.7(0.47)	102.7(17.99)	54.7(0.47)	51.3(0.47)	51.3(17.99)	27.3(0.47)	15.6(3.43)	0.0(0.00)	0.0(0.00)
ulcerative colitis	34.0(0.00)	34.0(1.68)	27.3(0.47)	17.0(0.00)	17.0(1.68)	13.7(0.47)	5.1(1.70)	0.0(0.00)	0.0(0.00)
unipolar depression	80.7(0.47)	80.7(3.49)	59.3(0.47)	40.3(0.47)	40.3(3.49)	29.7(0.47)	22.9(4.20)	0.0(0.00)	0.0(0.00)

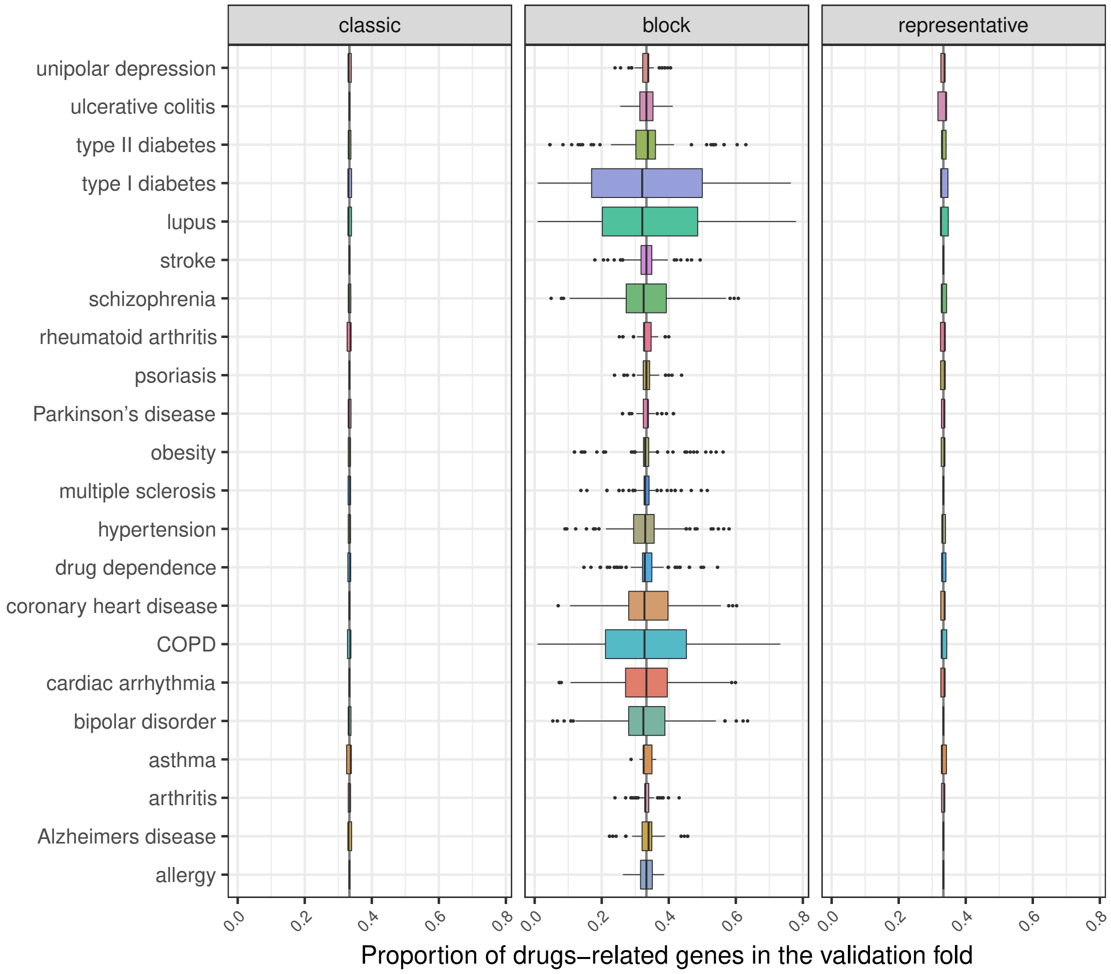

Figure I: Data balance by cross-validation strategy. Each boxplot summarises the folds for a particular disease and cross-validation strategy, whilst the vertical grey line corresponds to the theoretical balanced proportion. Due to their definition, the classic and representative strategies keep the dataset balanced: one third of the drugs-related genes are used to validate and two thirds are used as seed genes. Inevitably, small deviations arise if the total number of disease genes is not a multiple of 3. Note, however, how the block scheme sometimes keeps the balance (diseases such as asthma and Parkinson's disease), but can lead to data imbalance if large complexes are involved, like in COPD and type I diabetes.

Raw metrics plots

By method

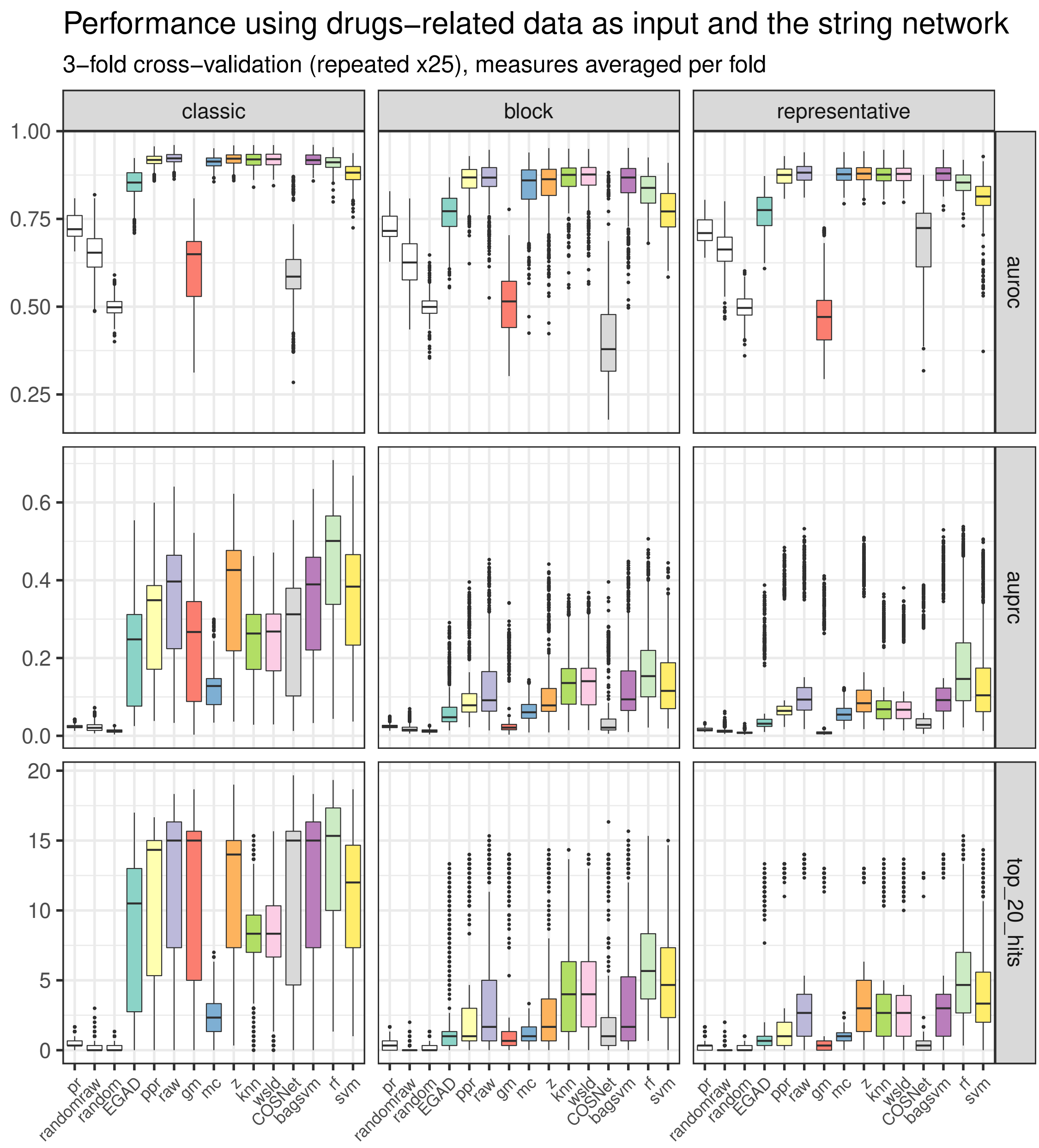

Figure J: Performance by method using drugs input and the STRING network. Methods `pr`, `randomraw` and `random` have no fill colour to represent their "null model" role.

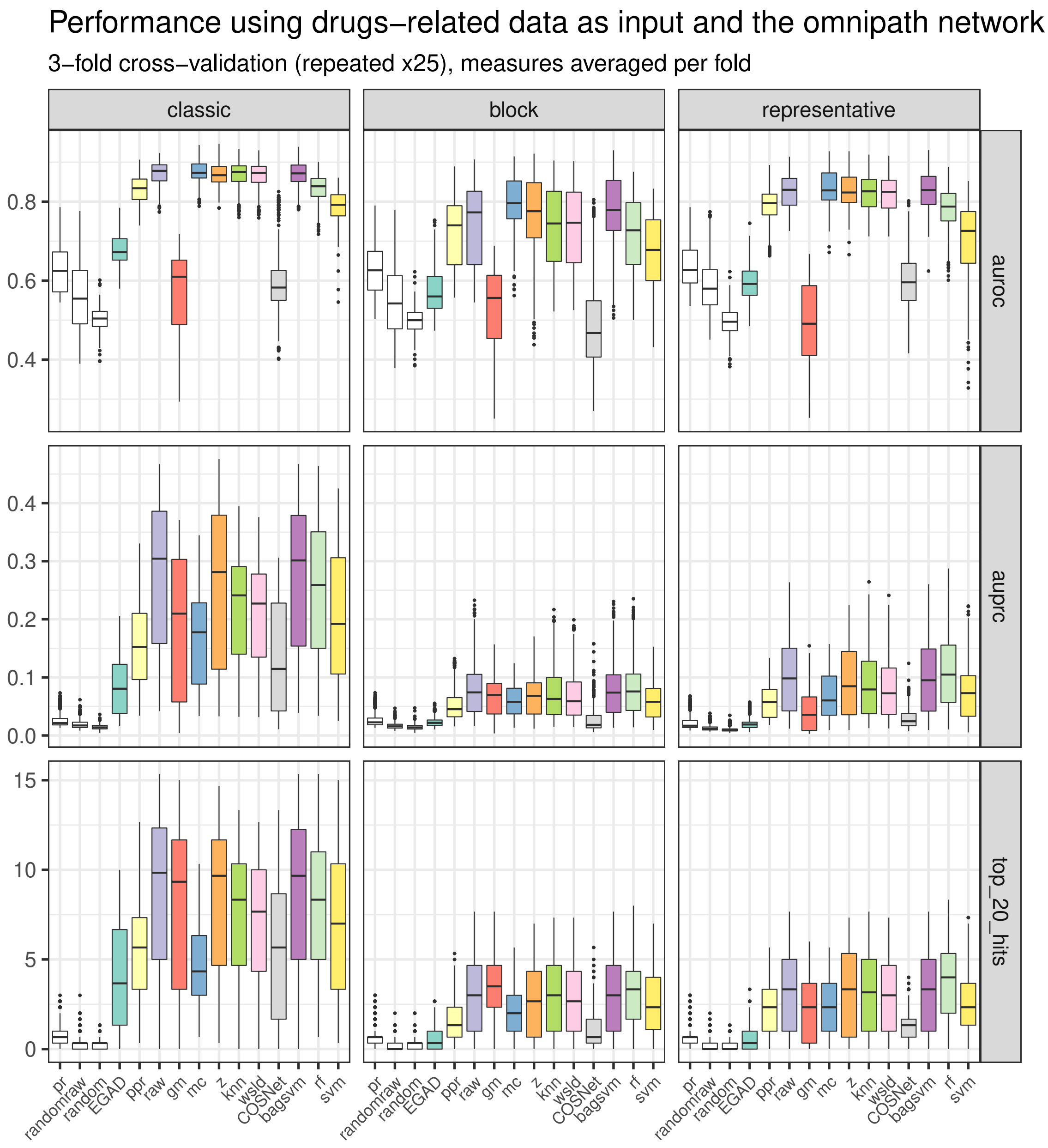

Figure K: Performance by method using drugs input and the OmniPath network. Methods `pr`, `randomraw` and `random` have no fill colour to represent their "null model" role.

By disease

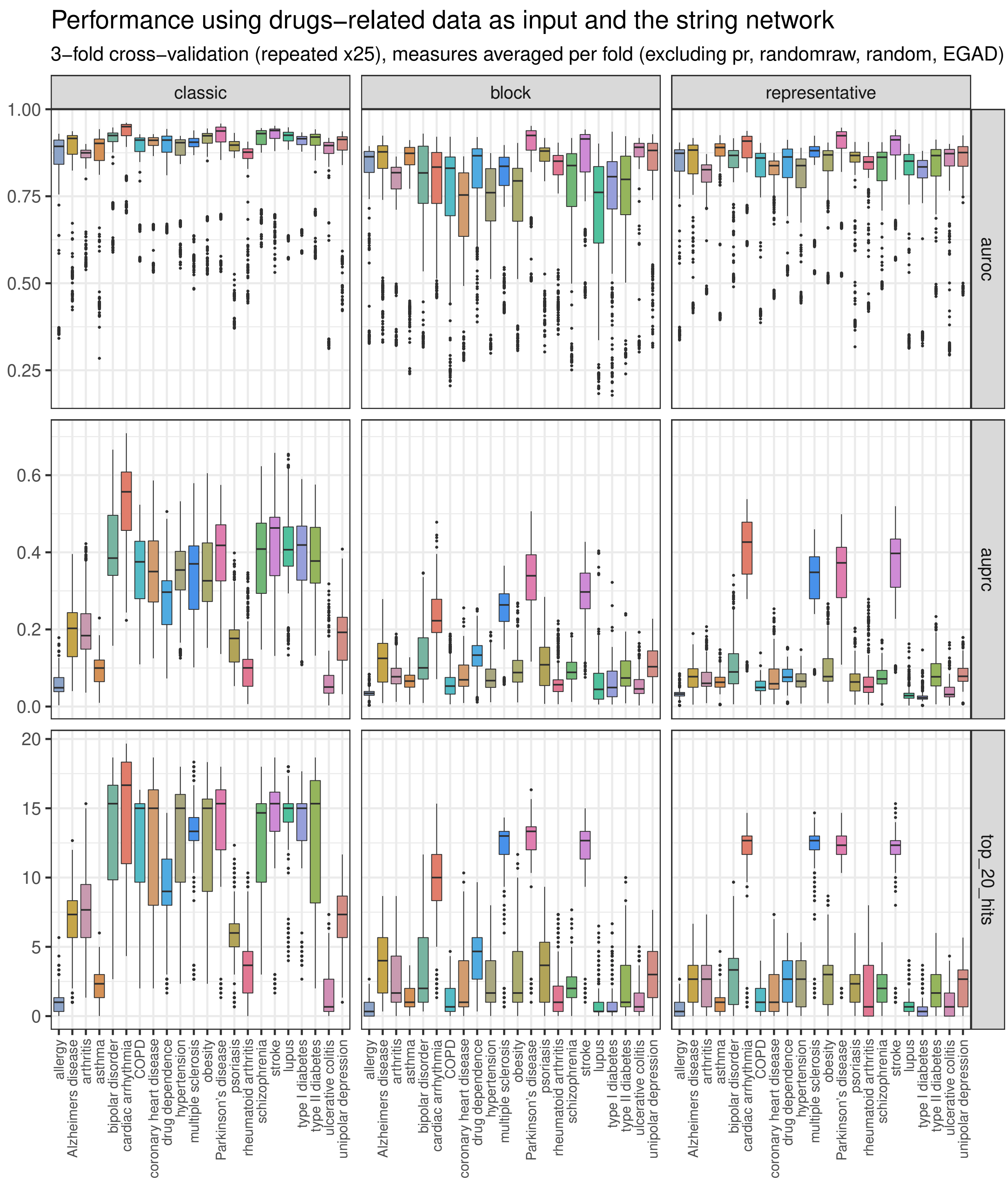

Figure L: Performance by disease using drugs input and the STRING network. Baseline methods `pr`, `randomraw`, `random` and `EGAD` are left out for visual clarity.

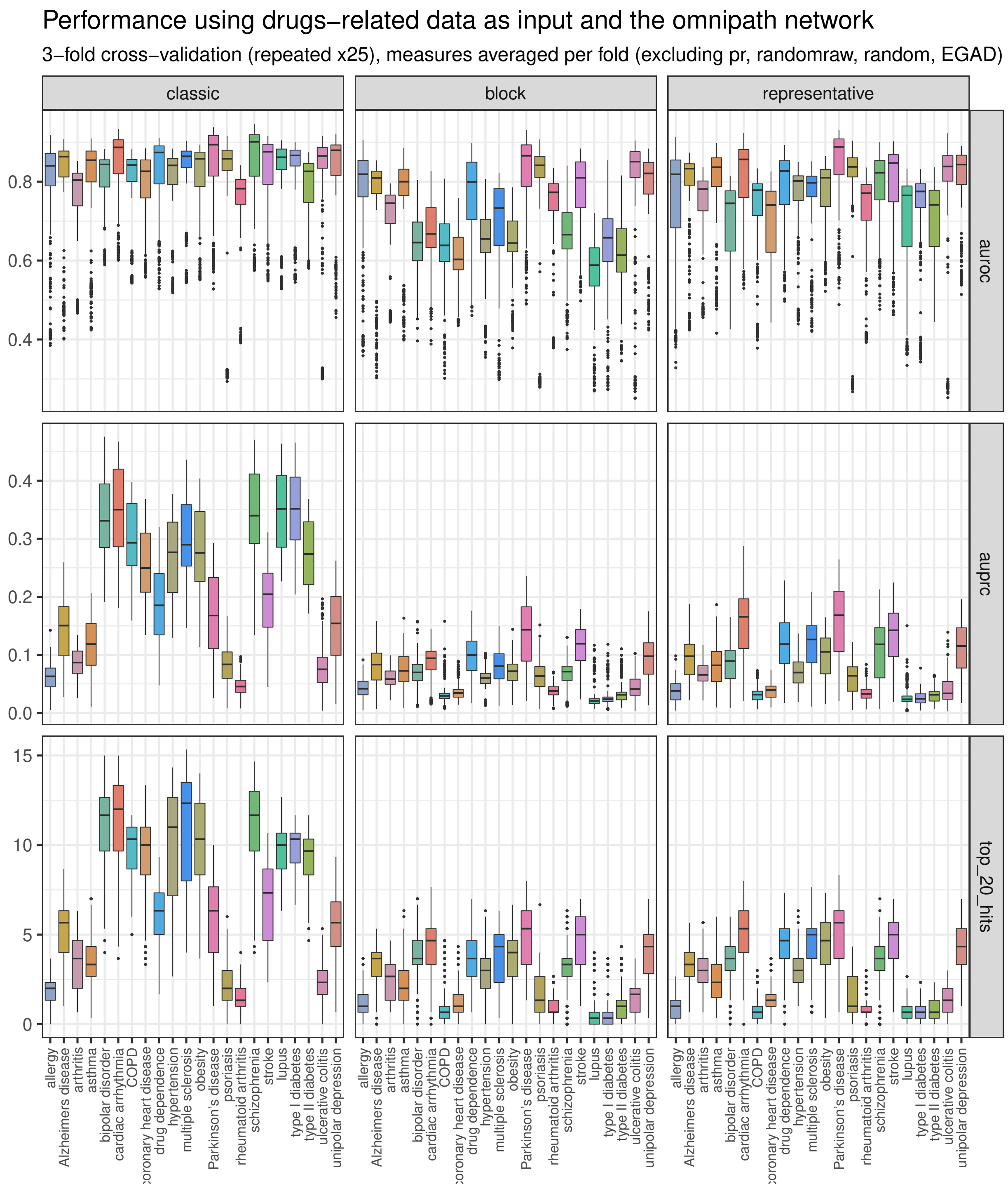

Figure M: Performance by disease using drugs input and the OmniPath network. Baseline methods `pr`, `randomraw`, `random` and `EGAD` are left out for visual clarity.

Overall performance by disease

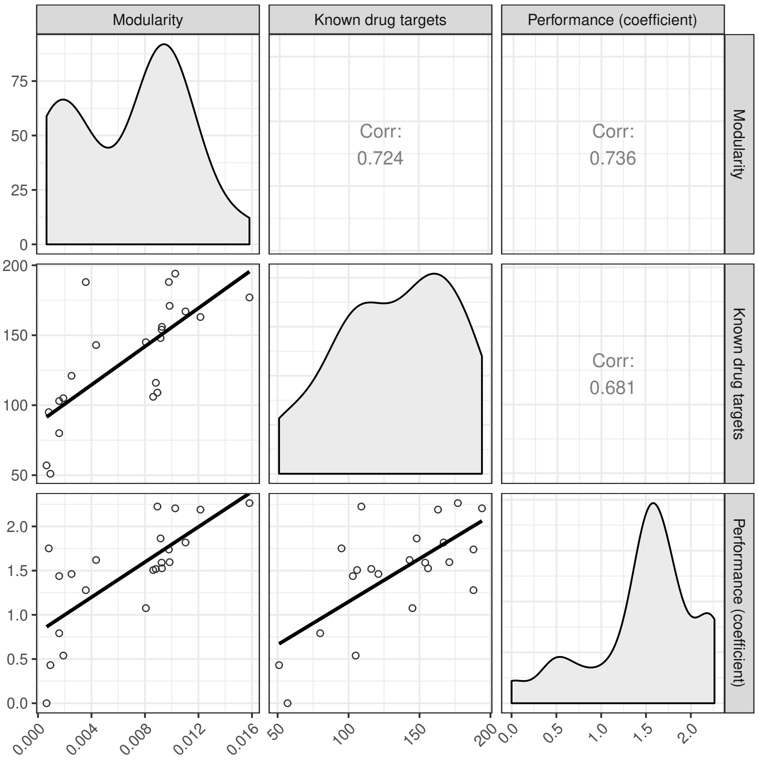

Figure N: Pairs plot involving disease-level performance (top 20 hits), number of known drug targets and disease modularity (in STRING). The three magnitudes correlate positively, implying that in general diseases with (i) more drugs-related genes and (ii) higher modularity in the network used by the prioritisers will exhibit better performance. Likewise, diseases with larger gene lists tend to be more modular.

Network-based methods

Method details

All tests and batch runs were set-up and conducted using the R statistical programming language [36]. When no R package was available, the methodology was re-implemented, building upon existing R packages whenever possible. Standard R machine learning libraries were used to train the support vector machine and random forest classifiers. Only the MashUp algorithm [35] required feature generation outside of the R environment, using the Matlab code from their publication. The versions of the R packages can be found in table T.

EGAD (Extending "Guilt by Association" by Degree [27]) was used here as a baseline comparator. EGAD performs a naïve diffusion approach via near-neighbours voting since EGAD's *neighbor_voting* function uses the adjacency matrix of the network and no additional parameters.

PageRank is a standard web ranking technology based upon the original work of Page et al. [26]. The igraph R package implementation of PageRank (here `ppr`) was used with default damping factor, `d = 0.85`. The latter implements what is commonly referred to as personalised PageRank, because of the custom prior distribution. This prior gives a probability of $1/n_{\text{input}}$ to each input gene and 0 otherwise, and forces random walks to start from the input genes. PageRank has been employed to diffuse disease seeding information across a two-layered network comprising PPI and GO hierarchy information [28]. Two approaches were developed: BirgRank (applying traditional PageRank with fixed decay parameters) and AptRank (with an adaptive diffusion mechanism). Here we considered only fixed decay parameter PageRank diffusion on the regular, weighted PPI network.

The diffuStats Bioconductor package [39] implements a variety of diffusion kernels and scoring schemes. Here we employed the regularised Laplacian kernel with the following diffusion propagation scores, as summarised in [39]: `raw`, `gm` (Genemania-based weighting for positives, negatives and unlabelled nodes), `mc` and `z`. `raw` comes from [29], while `gm` uses the weighting scheme from [30]. `mc` was inspired in [BMR$^+$16] and `z` is an exact version of [EBEK11] without controlling for degree; both have been used for the enrichment of metabolomics data [31]. The regularised Laplacian kernel had the following (default) parameters: `sigma2 = 1`, `add_diag = 1`, `normalized = FALSE`. The weights from the network are scaled to lie in $[0, 1]$.

RANKS (RAnking of Nodes with Kernelized Score functions [32]) employs kernelised score functions in semi-supervised learning (here `knn` and `wsld`), and has been assessed for disease gene identification [42]. Default package settings were used in all cases (number of neighbours, $k = 3$ for k-nn and coefficient of linear decay, `d = 2` for `wsld`). We used the kernel computed with diffuStats.

The bagging SVM method (here `bagsvm`) is an implementation of ProDiGe1 [34]. It approximates a form of PU-learning [40,41] by iteratively choosing random subsets from the unlabelled genes (i.e. those genes that are not known to be associated with the disease) when training classifiers. This method was directly applied to the regularised Laplacian kernel computed with diffuStats.

The `svm` (Support Vector Machine; kernlab R package) and `rf` (randomForest R package) methods apply classical machine learning approaches on network-based features. Network-based features were generated using MashUp with default parameters for the human network (800-dimensional, as recommended by the authors) [35]. We used the caret [43] and mlr [44] R packages to define the classification tasks, grid-search the parameters and make predictions for these two methods.

The SVM method used here is a nu-SVM with RBF kernel. In training, the negative class examples were randomly under-sampled to match the number of positive class examples. Parameters were determined via inner cross-validation with parameter ranges of $(0.1, 0.9)$ for `nu` and $(10^{-6}, 10^{2})$ for `sigma`, with search space linear on `nu` and logarithmic on `sigma`. A grid of resolution 5 in each direction was explored to choose the best parameters, with an internal loop of 3 repetitions of 3-fold CV.

Random forest parameters were set to default values (see mlr documentation on *classif.randomForest*) apart from those tuned via inner cross validation. These were ranges of $(10, 500)$ for `ntree` and $(1, 5)$ for `nodesize`, with linear search space in both. A grid of resolution 3 in each direction was explored to choose optimal parameters with an internal loop of 3 repetitions of standard 3-fold CV.

COSNet (COst Sensitive neural Network [33,47]) consists of a parametric Hopfield recurrent neural network classifier, employed within a semi-supervised, cost-sensitive learning context to deal with networks seeded with highly unbalanced labellings. The cost (regularisation) parameter in the COSNet R package was set to 0.0001 following the documentation guidelines.

Finally, we included the following three naive baseline methods, for comparison purposes: (1) `pr`, a classic problem naïve 'non-personalised' PageRank implementation where input scores on the genes are ignored; (2) `randomraw`, which applies the `raw` diffusion approach from diffuStats [39] to randomly permuted input scores on the genes; and (3) `random`, a uniform re-ranking of input genes without any network propagation (using *sample(n)* in R, with `n =` number of genes in the test fold).

Comparing methods

Comparing methods using their predictions on seed and novel genes can give insights on similarities and differences among the various methods families. The main body contains a comparison using the drugs genes as seeds and excluding the seeds for the comparison, while here we also include the seeds (figure O) and an analogous analysis on the genetically associated genes (figure P). Classical MDS plots for specific diseases can be found in the supplementary file `mds_plots.zip` and are qualitatively consistent with their multiview counterparts.

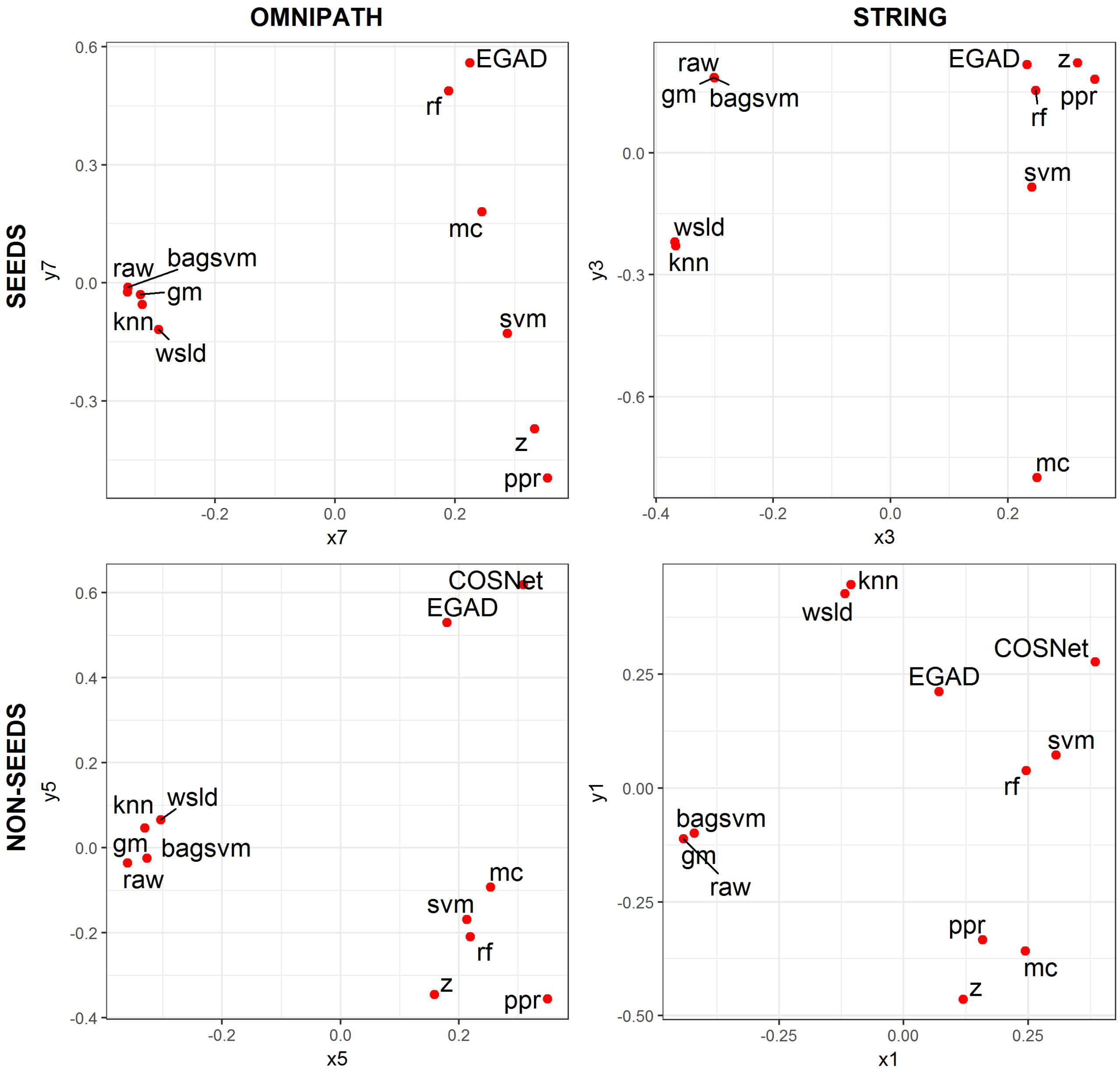

Figure O: Multi-view MDS plot displaying the preserved Spearman's footrule distances representing the differential ranking behaviours of methods across all 22 diseases when individual sets of drugs seeds were input. Each plot is for a different combination of input network (columns) and the predicted gene set that was ranked (rows). Note how COSNet is excluded from seeds prediction, as by its definition it does not order the seeds.

In figure O, two groups of diffusion-based methods consistently clustered together: (i) `raw`, `gm`, `bagsvm`, and (ii) `knn` and `wsld`. As a consequence, the supervised, bagged SVM based in the regularised Laplacian kernel behaved like usual diffusion scores (`raw`) that use the same kernel. Despite their common background, groups (i) and (ii) appeared together in OmniPath but not in STRING, implying that even small methodological differences can have a noticeable overall impact. A third group (iii) was formed by `ppr`, `z` and `mc`, although the latter did not cluster as clearly in some cases. These methods are also diffusion-based, but `mc` and `z` have statistical differences with `raw` [39] and this is reflected in the MDS plot. The supervised methods (iv) `rf` and `svm` also tended to agree, since they were trained on the same network-based features. Finally, (v) `EGAD` and `COSNet` closes the method grouping, suggesting that the artificial neural network from `COSNet` resembled

neighbour voting approaches.

In figure P, groups (i) and (ii) stick together in all the scenarios and become one single family. Group (iii) is only obvious in STRING and non-seed genes, becoming diluted in the rest.

The tight five method group (`raw`, `gm`, `bagsvm`, `knn` and `wsld`), seen only for OmniPath with input drug seeds in the main body, is apparent with both networks. Group (iv) and (v) still behaves as so, further justifying this classification. Despite some differences, these groupings do agree to those seen for the corresponding networks under drug seed input in figure O.

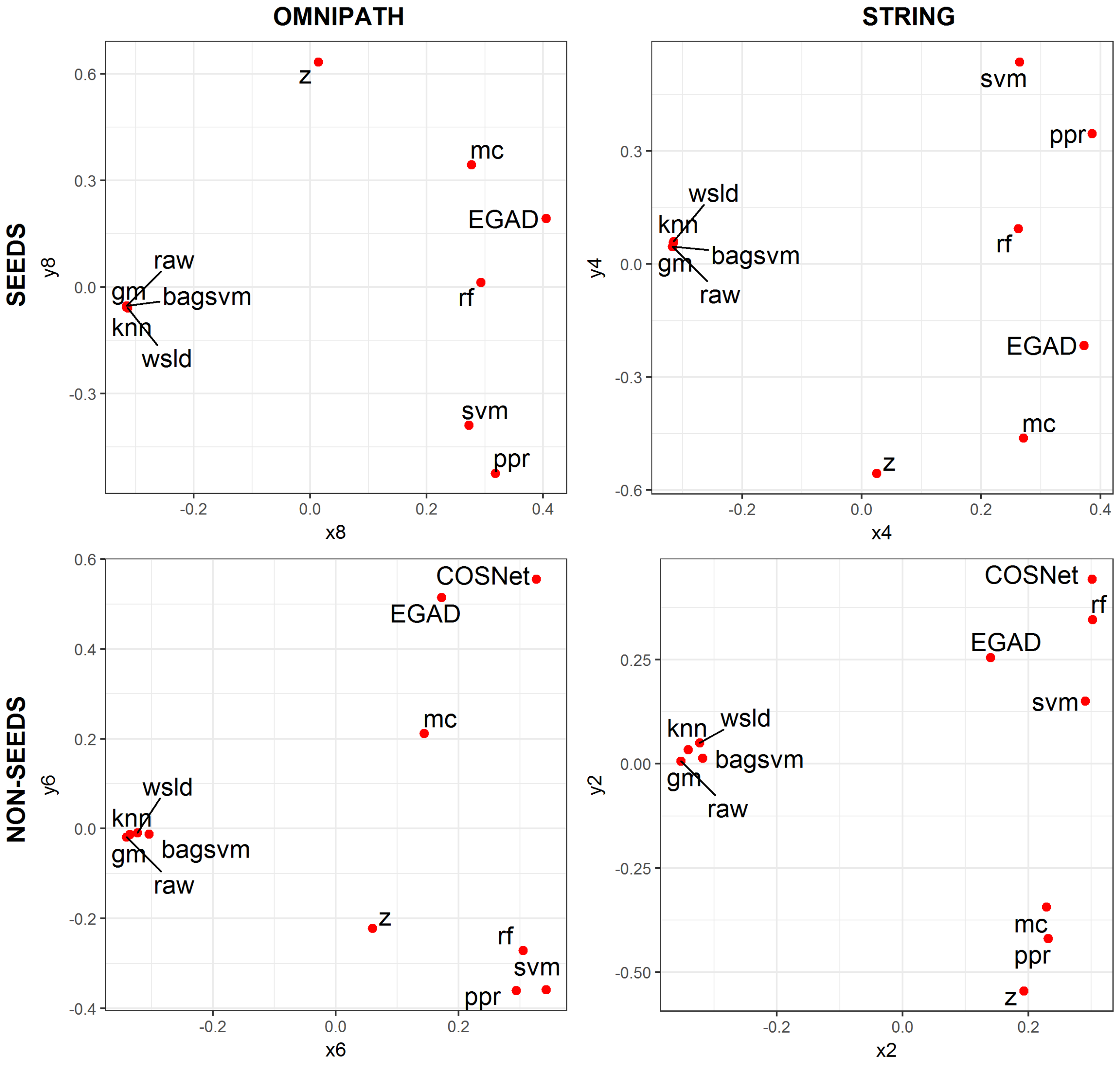

Figure P: Multi-view MDS plot displaying the preserved Spearman's footrule distances representing the differential ranking behaviours of methods across all 22 diseases when individual sets of genetic seeds were input.

Methods ranking using all the metrics

In the main text we show the method prioritisations using the main metrics. Figure Q contains the same data for all the metrics hereby analysed. The metrics have been arranged from farthest (top 20 hits) to closest to AUROC. Two conclusions can be drawn from figure Q. First, AUROC behaves differently from the other five metrics, which in turn behave alike. This is expected as AUPRC, pAUROC and top k hits emphasise on the performance at the top ranked entities. Second, as the parameter of pAUROC and top k hits grows, both metrics rank closer to AUROC, which is also natural.

The fact that top 20 hits, top 100 hits and AUPRC behave so similarly suggests that the ranking under top k hits is robust for small values of k ($k \leq 100$) and that AUPRC is indeed a meaningful performance metric for real scenarios in drug development.

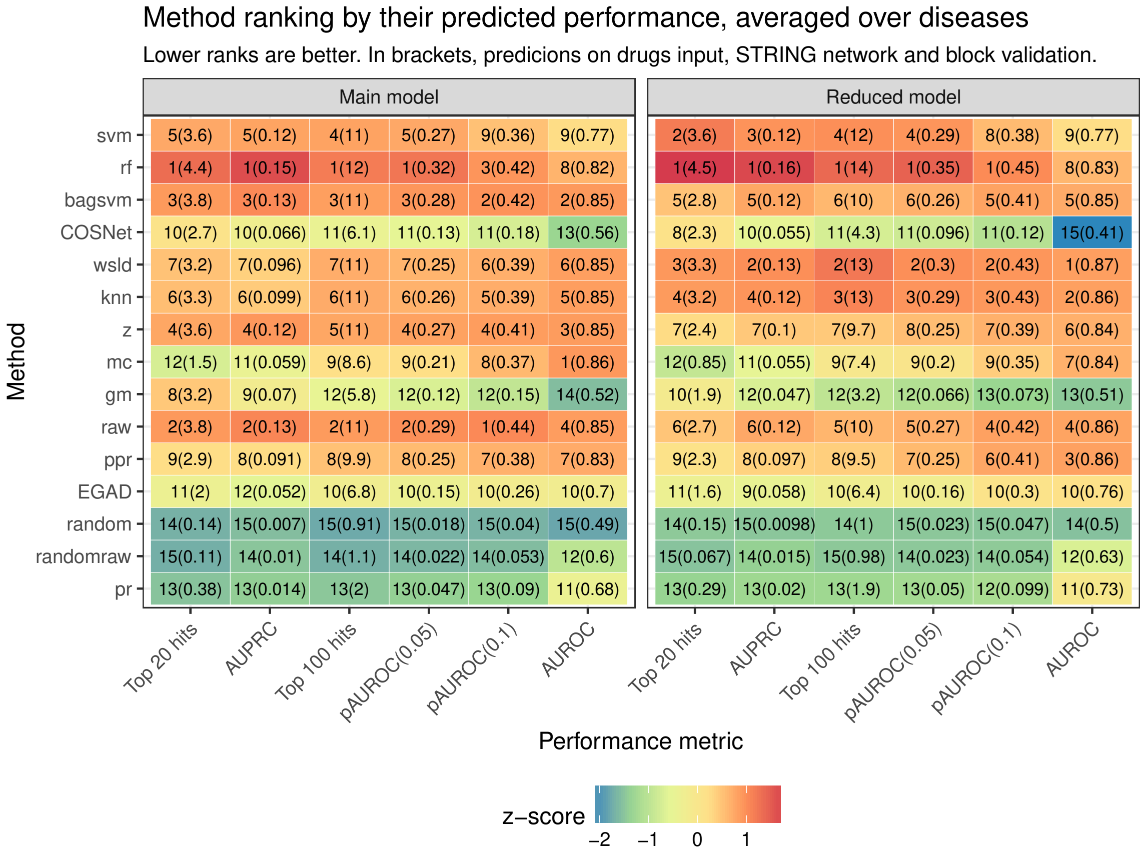

Figure Q: Ranking of all the methods, using the predictions of the main and the reduced models on the drugs input, STRING network, block cross validation and averaging over diseases. A column-wise z-score on the predicted mean is depicted, in order to illustrate the magnitude of the difference.

Model summaries and confidence intervals on predictions

Model description

Table F: Summary of all the complete models fitted in this study. Models are adjusted separately by input type, not to mix notably different patterns. The model formulae are R-like, where the left hand side contains the response and the right hand side describes the independent variables. In all these models, the reference levels are the **pr** method, **STRING** network, **classic** cross-validation scheme and **allergy**. Statistical significance on each coefficient is computed by comparing the full model with the model that lacks such regressor.

Model	Input type	Model type	Family	Formula
DA1	Drugs	Additive	Quasibinomial	AUROC ∼ method + cv_scheme + network + disease
DA2	Drugs	Additive	Quasibinomial	AUPRC ∼ method + cv_scheme + network + disease
DA3	Drugs	Additive	Quasipoisson	Top20 ∼ method + cv_scheme + network + disease
DA4	Drugs	Additive	Quasibinomial	pAUROC0.1∼ method + cv_scheme + network + disease
DA5	Drugs	Additive	Quasibinomial	pAUROC0.05 ∼ method + cv_scheme + network + disease
DA6	Drugs	Additive	Quasipoisson	Top100 ∼ method + cv_scheme + network + disease
GA1	Genetic	Additive	Quasibinomial	AUROC ∼ method + cv_scheme + network + disease
GA2	Genetic	Additive	Quasibinomial	AUPRC ∼ method + cv_scheme + network + disease
GA3	Genetic	Additive	Quasipoisson	Top20 ∼ method + cv_scheme + network + disease
GA4	Genetic	Additive	Quasibinomial	pAUROC0.1∼ method + cv_scheme + network + disease
GA5	Genetic	Additive	Quasibinomial	pAUROC0.05 ∼ method + cv_scheme + network + disease
GA6	Genetic	Additive	Quasipoisson	Top100 ∼ method + cv_scheme + network + disease
SA1	Stream	Additive	Quasibinomial	AUROC ∼ method + cv_scheme + network + disease
SA2	Stream	Additive	Quasibinomial	AUPRC ∼ method + cv_scheme + network + disease
SA3	Stream	Additive	Quasipoisson	Top20 ∼ method + cv_scheme + network + disease
SA4	Stream	Additive	Quasibinomial	pAUROC0.1∼ method + cv_scheme + network + disease
SA5	Stream	Additive	Quasibinomial	pAUROC0.05 ∼ method + cv_scheme + network + disease
SA6	Stream	Additive	Quasipoisson	Top100 ∼ method + cv_scheme + network + disease

Table G: Summary of all the reduced models. These additive models have been fitted to the most relevant scenario: **drugs** input, **STRING** network and **block** cross-validation strategy. In all these models, the reference levels are the **pr** method and **allergy**.

Model	Input type	Model type	Family	Formula
DA1r	Drugs	Additive	Quasibinomial	AUROC ∼ method + disease
DA2r	Drugs	Additive	Quasibinomial	AUPRC ∼ method + disease
DA3r	Drugs	Additive	Quasipoisson	Top20 ∼ method + disease
DA4r	Drugs	Additive	Quasibinomial	pAUROC0.1∼ method + disease
DA5r	Drugs	Additive	Quasibinomial	pAUROC0.05 ∼ method + disease
DA6r	Drugs	Additive	Quasipoisson	Top100 ∼ method + disease

Drugs input

Additive models

Table H: Models for the metrics auroc, auprc, top_20_hits using the drugs input (model names DA1, DA2 and DA3)

	auroc	auprc	top_20_hits
Constant	1.264*** (1.243, 1.285)	−4.286*** (−4.346, −4.227)	−1.462*** (−1.539, −1.385)
methodrandomraw	−0.328*** (−0.345, −0.312)	−0.308*** (−0.372, −0.244)	−1.223*** (−1.334, −1.112)
methodrandom	−0.773*** (−0.790, −0.757)	−0.685*** (−0.756, −0.613)	−0.994*** (−1.096, −0.892)
methodEGAD	0.122*** (0.105, 0.139)	1.358*** (1.310, 1.406)	1.662*** (1.604, 1.720)
methodppr	0.861*** (0.842, 0.880)	1.964*** (1.917, 2.010)	2.022*** (1.965, 2.078)
methodraw	0.990*** (0.970, 1.009)	2.352*** (2.306, 2.397)	2.299*** (2.244, 2.355)
methodgm	−0.652*** (−0.668, −0.636)	1.681*** (1.634, 1.728)	2.126*** (2.070, 2.182)
methodmc	1.044*** (1.024, 1.064)	1.488*** (1.440, 1.536)	1.376*** (1.317, 1.436)
methodz	1.005*** (0.986, 1.025)	2.286*** (2.241, 2.332)	2.253*** (2.197, 2.308)
methodknn	0.981*** (0.962, 1.001)	2.060*** (2.014, 2.106)	2.162*** (2.106, 2.217)
methodwsld	0.976*** (0.956, 0.995)	2.028*** (1.982, 2.074)	2.148*** (2.092, 2.204)
methodCOSNet	−0.511*** (−0.527, −0.494)	1.615*** (1.568, 1.662)	1.962*** (1.906, 2.019)
methodbagsvm	1.028*** (1.008, 1.048)	2.337*** (2.292, 2.383)	2.299*** (2.243, 2.354)
methodrf	0.782*** (0.763, 0.801)	2.569*** (2.524, 2.615)	2.454*** (2.399, 2.509)
methodsvm	0.462*** (0.445, 0.480)	2.233*** (2.187, 2.279)	2.246*** (2.190, 2.302)
cv_schemeblock	−0.441*** (−0.450, −0.433)	−1.243*** (−1.256, −1.230)	−0.984*** (−0.997, −0.970)
cv_schemerepresentative	−0.218*** (−0.227, −0.210)	−1.182*** (−1.195, −1.169)	−1.003*** (−1.017, −0.990)
networkomnipath	−0.392*** (−0.399, −0.385)	−0.517*** (−0.528, −0.506)	−0.357*** (−0.367, −0.346)
diseaseAlzheimers disease	−0.001 (−0.024, 0.022)	1.081*** (1.032, 1.131)	1.439*** (1.377, 1.500)
diseasearthritis	−0.192*** (−0.214, −0.169)	0.846*** (0.795, 0.897)	1.279*** (1.216, 1.341)
diseaseasthma	0.012 (−0.011, 0.035)	0.671*** (0.618, 0.723)	0.792*** (0.725, 0.859)
diseasebipolar disorder	−0.211*** (−0.234, −0.188)	1.652*** (1.604, 1.699)	1.864*** (1.805, 1.924)
diseasecardiac arrhythmia	0.007 (−0.016, 0.030)	2.291*** (2.245, 2.337)	2.264*** (2.206, 2.322)
diseaseCOPD	−0.188*** (−0.210, −0.165)	1.301*** (1.252, 1.350)	1.519*** (1.457, 1.580)
diseasecoronary heart disease	−0.299*** (−0.321, −0.276)	1.299*** (1.250, 1.348)	1.596*** (1.535, 1.656)
diseasedrug dependence	−0.018 (−0.041, 0.005)	1.356*** (1.308, 1.405)	1.620*** (1.559, 1.681)
diseasehypertension	−0.207*** (−0.230, −0.185)	1.372*** (1.324, 1.421)	1.739*** (1.679, 1.799)
diseasemultiple sclerosis	−0.071*** (−0.094, −0.048)	1.970*** (1.923, 2.017)	2.225*** (2.167, 2.283)
diseaseobesity	−0.164*** (−0.186, −0.141)	1.506*** (1.458, 1.554)	1.819*** (1.759, 1.878)
diseaseParkinson's disease	0.207*** (0.184, 0.231)	2.080*** (2.034, 2.127)	2.205*** (2.147, 2.263)
diseasepsoriasis	0.083*** (0.059, 0.106)	0.856*** (0.804, 0.907)	1.076*** (1.012, 1.140)
diseaserheumatoid arthritis	−0.163*** (−0.185, −0.140)	0.355*** (0.300, 0.410)	0.539*** (0.469, 0.609)
diseaseschizophrenia	−0.083*** (−0.106, −0.060)	1.603*** (1.555, 1.651)	1.752*** (1.692, 1.812)
diseasestroke	0.085*** (0.062, 0.109)	2.057*** (2.011, 2.104)	2.190*** (2.132, 2.249)
diseaselupus	−0.248*** (−0.270, −0.225)	1.396*** (1.347, 1.444)	1.526*** (1.465, 1.587)
diseasetype I diabetes	−0.174*** (−0.196, −0.151)	1.364*** (1.316, 1.413)	1.506*** (1.445, 1.568)
diseasetype II diabetes	−0.252*** (−0.274, −0.229)	1.373*** (1.324, 1.421)	1.590*** (1.530, 1.651)
diseaseulcerative colitis	0.132*** (0.108, 0.155)	0.251*** (0.195, 0.308)	0.431*** (0.360, 0.502)
diseaseunipolar depression	0.022* (−0.002, 0.045)	1.113*** (1.064, 1.163)	1.462*** (1.400, 1.523)
Observations	49,500	49,500	49,500
Note:			*p<0.1; **p<0.05; ***p<0.01

Table I: Predictions of the models DA1, DA2, DA3 (95% confidence intervals after averaging over disease).

Input: drugs data		STRING			OmniPath		
metric	method	classic	block	representative	classic	block	representative
	pr	(0.764, 0.768)	(0.675, 0.681)	(0.722, 0.727)	(0.686, 0.691)	(0.584, 0.590)	(0.637, 0.643)
	randomraw	(0.699, 0.705)	(0.599, 0.606)	(0.652, 0.657)	(0.611, 0.617)	(0.503, 0.509)	(0.558, 0.565)
	random	(0.599, 0.605)	(0.490, 0.496)	(0.545, 0.552)	(0.502, 0.508)	(0.393, 0.399)	(0.448, 0.454)
	EGAD	(0.785, 0.789)	(0.701, 0.707)	(0.746, 0.751)	(0.711, 0.717)	(0.613, 0.620)	(0.665, 0.671)
	ppr	(0.884, 0.887)	(0.831, 0.835)	(0.860, 0.864)	(0.837, 0.842)	(0.768, 0.774)	(0.805, 0.810)
	raw	(0.896, 0.900)	(0.848, 0.852)	(0.874, 0.878)	(0.854, 0.858)	(0.790, 0.795)	(0.825, 0.829)
	gm	(0.627, 0.633)	(0.520, 0.526)	(0.575, 0.581)	(0.532, 0.538)	(0.423, 0.429)	(0.478, 0.484)
auroc	mc	(0.901, 0.904)	(0.855, 0.859)	(0.880, 0.884)	(0.861, 0.865)	(0.799, 0.804)	(0.832, 0.837)
	z	(0.898, 0.901)	(0.850, 0.854)	(0.876, 0.880)	(0.856, 0.860)	(0.793, 0.798)	(0.827, 0.832)
	knn	(0.896, 0.899)	(0.847, 0.851)	(0.873, 0.877)	(0.853, 0.857)	(0.789, 0.794)	(0.823, 0.828)
	wsld	(0.895, 0.898)	(0.846, 0.850)	(0.873, 0.877)	(0.852, 0.856)	(0.788, 0.793)	(0.823, 0.827)
	COSNet	(0.660, 0.666)	(0.555, 0.561)	(0.609, 0.615)	(0.567, 0.573)	(0.457, 0.464)	(0.513, 0.519)
	bagsvm	(0.900, 0.903)	(0.853, 0.857)	(0.879, 0.882)	(0.859, 0.863)	(0.796, 0.802)	(0.830, 0.835)
	rf	(0.876, 0.879)	(0.819, 0.824)	(0.850, 0.854)	(0.826, 0.831)	(0.754, 0.760)	(0.793, 0.798)
	svm	(0.837, 0.841)	(0.767, 0.772)	(0.805, 0.809)	(0.776, 0.781)	(0.690, 0.696)	(0.736, 0.741)
	pr	(0.045, 0.048)	(0.013, 0.014)	(0.014, 0.015)	(0.027, 0.029)	(0.008, 0.009)	(0.008, 0.009)
	randomraw	(0.033, 0.036)	(0.010, 0.011)	(0.010, 0.011)	(0.020, 0.022)	(0.006, 0.006)	(0.006, 0.007)
	random	(0.023, 0.025)	(0.007, 0.007)	(0.007, 0.008)	(0.014, 0.015)	(0.004, 0.004)	(0.004, 0.005)
	EGAD	(0.156, 0.162)	(0.051, 0.053)	(0.054, 0.056)	(0.099, 0.104)	(0.031, 0.032)	(0.033, 0.034)
	ppr	(0.254, 0.261)	(0.089, 0.093)	(0.094, 0.098)	(0.168, 0.174)	(0.055, 0.057)	(0.058, 0.061)
	raw	(0.334, 0.343)	(0.126, 0.131)	(0.133, 0.138)	(0.230, 0.237)	(0.079, 0.082)	(0.084, 0.087)
	gm	(0.204, 0.211)	(0.069, 0.072)	(0.073, 0.076)	(0.132, 0.137)	(0.042, 0.044)	(0.045, 0.047)
auprc	mc	(0.174, 0.181)	(0.057, 0.060)	(0.061, 0.063)	(0.111, 0.116)	(0.035, 0.037)	(0.037, 0.039)
	z	(0.320, 0.328)	(0.119, 0.124)	(0.126, 0.130)	(0.219, 0.225)	(0.075, 0.078)	(0.079, 0.082)
	knn	(0.272, 0.280)	(0.097, 0.101)	(0.103, 0.107)	(0.182, 0.189)	(0.060, 0.063)	(0.064, 0.067)
	wsld	(0.266, 0.274)	(0.095, 0.098)	(0.100, 0.104)	(0.178, 0.184)	(0.059, 0.061)	(0.062, 0.065)
	COSNet	(0.193, 0.200)	(0.064, 0.067)	(0.068, 0.071)	(0.125, 0.130)	(0.039, 0.041)	(0.042, 0.044)
	bagsvm	(0.331, 0.339)	(0.125, 0.129)	(0.131, 0.136)	(0.228, 0.234)	(0.078, 0.081)	(0.083, 0.086)
	rf	(0.384, 0.393)	(0.152, 0.158)	(0.160, 0.166)	(0.271, 0.278)	(0.097, 0.100)	(0.102, 0.106)
	svm	(0.308, 0.316)	(0.114, 0.118)	(0.120, 0.124)	(0.210, 0.216)	(0.071, 0.074)	(0.075, 0.078)
	pr	(0.96, 1.07)	(0.36, 0.40)	(0.35, 0.39)	(0.67, 0.75)	(0.25, 0.28)	(0.25, 0.27)
	randomraw	(0.27, 0.33)	(0.10, 0.12)	(0.10, 0.12)	(0.19, 0.23)	(0.07, 0.09)	(0.07, 0.08)
	random	(0.34, 0.41)	(0.13, 0.15)	(0.13, 0.15)	(0.24, 0.29)	(0.09, 0.11)	(0.09, 0.11)
	EGAD	(5.21, 5.46)	(1.94, 2.05)	(1.91, 2.01)	(3.64, 3.83)	(1.36, 1.43)	(1.33, 1.41)
	ppr	(7.49, 7.80)	(2.80, 2.92)	(2.74, 2.87)	(5.24, 5.47)	(1.96, 2.05)	(1.92, 2.01)
	raw	(9.91, 10.28)	(3.70, 3.85)	(3.63, 3.78)	(6.93, 7.20)	(2.59, 2.70)	(2.54, 2.64)
	gm	(8.32, 8.65)	(3.10, 3.24)	(3.04, 3.18)	(5.82, 6.06)	(2.17, 2.27)	(2.13, 2.23)
top_20_hits	mc	(3.90, 4.12)	(1.46, 1.54)	(1.43, 1.51)	(2.73, 2.89)	(1.02, 1.08)	(1.00, 1.06)
	z	(9.45, 9.81)	(3.53, 3.68)	(3.46, 3.60)	(6.61, 6.87)	(2.47, 2.57)	(2.42, 2.52)
	knn	(8.62, 8.96)	(3.22, 3.36)	(3.16, 3.29)	(6.03, 6.28)	(2.25, 2.35)	(2.21, 2.31)
	wsld	(8.51, 8.84)	(3.17, 3.31)	(3.11, 3.25)	(5.95, 6.19)	(2.22, 2.32)	(2.18, 2.28)
	COSNet	(7.05, 7.36)	(2.63, 2.76)	(2.58, 2.70)	(4.93, 5.15)	(1.84, 1.93)	(1.81, 1.89)
	bagsvm	(9.90, 10.27)	(3.69, 3.85)	(3.62, 3.77)	(6.93, 7.19)	(2.58, 2.69)	(2.53, 2.64)
	rf	(11.58, 11.98)	(4.32, 4.49)	(4.24, 4.40)	(8.10, 8.39)	(3.02, 3.14)	(2.96, 3.08)
	svm	(9.39, 9.74)	(3.50, 3.65)	(3.44, 3.58)	(6.57, 6.83)	(2.45, 2.56)	(2.40, 2.51)

The **DA3** model was used in the main body to statistically compare method performances. We explored its diagnostic plots (figure R) to ensure we drew sound conclusions from it. The first panel in figure R contains the deviance residuals against the predicted values. The lack of tendencies in it, reflected by the flat red line, supports that the residuals are healthy and that the poisson is a suitable distribution to describe the data [Zuu09]. The fourth panel from figure R shows that there are no influential observations using Cook's distance statistic.

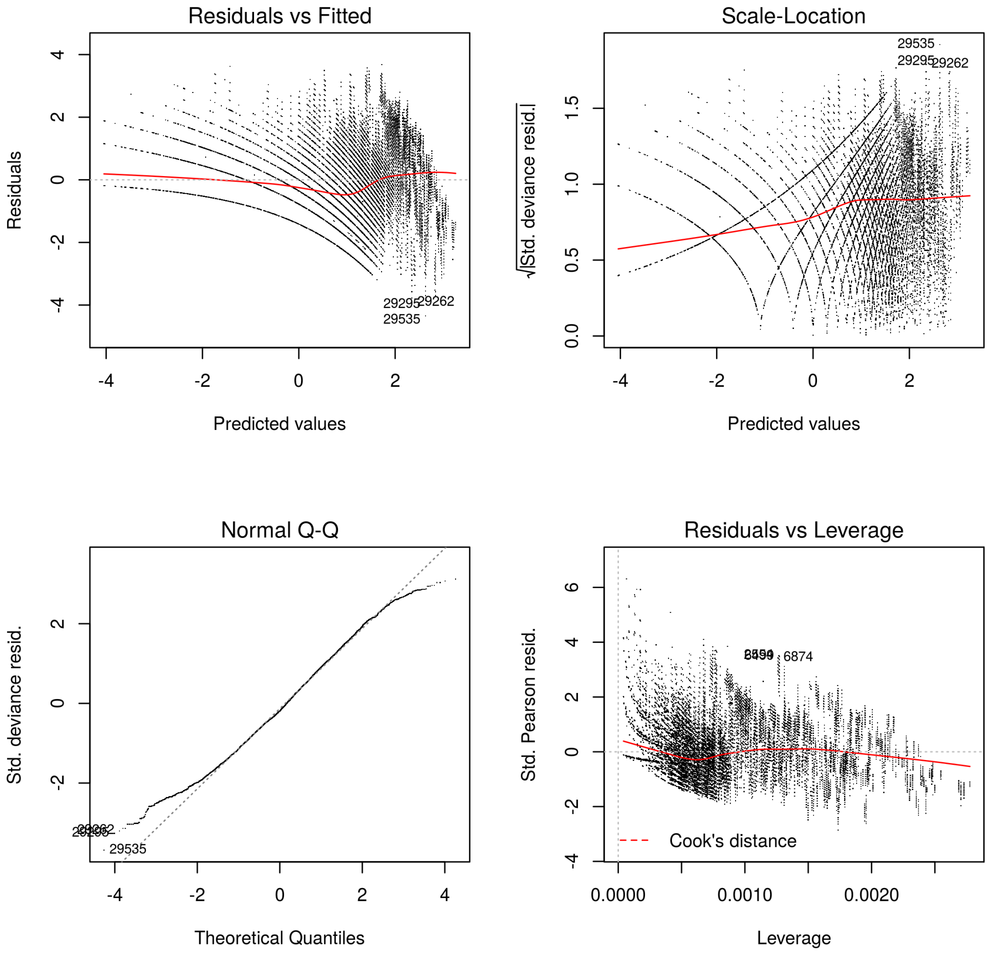

Figure R: Diagnostics plots for the top 20 hits quasipoisson model **DA3**.

Table J: Models for the metrics partial_auroc_0.10, partial_auroc_0.05, top_100_hits using the drugs input (model names DA4, DA5 and DA6)

	partial_auroc_0.10	partial_auroc_0.05	top_100_hits
Constant	−1.544*** (−1.575, −1.514)	−2.334*** (−2.372, −2.297)	0.533*** (0.496, 0.570)
methodrandomraw	−0.574*** (−0.610, −0.537)	−0.778*** (−0.828, −0.727)	−0.550*** (−0.593, −0.506)
methodrandom	−0.869*** (−0.908, −0.829)	−0.992*** (−1.046, −0.938)	−0.782*** (−0.829, −0.735)
methodEGAD	1.265*** (1.238, 1.293)	1.287*** (1.253, 1.322)	1.236*** (1.206, 1.266)
methodppr	1.838*** (1.811, 1.865)	1.907*** (1.874, 1.941)	1.606*** (1.577, 1.635)
methodraw	2.061*** (2.034, 2.088)	2.116*** (2.083, 2.149)	1.736*** (1.708, 1.765)
methodgm	0.549*** (0.519, 0.578)	1.005*** (0.970, 1.040)	1.079*** (1.048, 1.110)
methodmc	1.791*** (1.764, 1.819)	1.712*** (1.679, 1.745)	1.472*** (1.442, 1.501)
methodz	1.956*** (1.929, 1.983)	2.034*** (2.001, 2.067)	1.697*** (1.668, 1.726)
methodknn	1.862*** (1.835, 1.889)	1.955*** (1.922, 1.988)	1.683*** (1.654, 1.711)
methodwsld	1.851*** (1.824, 1.878)	1.936*** (1.903, 1.969)	1.674*** (1.645, 1.703)
methodCOSNet	0.792*** (0.764, 0.821)	1.140*** (1.105, 1.174)	1.121*** (1.091, 1.152)
methodbagsvm	2.011*** (1.985, 2.038)	2.089*** (2.056, 2.122)	1.724*** (1.695, 1.753)
methodrf	2.000*** (1.973, 2.027)	2.245*** (2.212, 2.278)	1.837*** (1.809, 1.866)
methodsvm	1.752*** (1.725, 1.780)	2.001*** (1.968, 2.034)	1.711*** (1.682, 1.739)
cv_schemeblock	−0.830*** (−0.841, −0.820)	−0.958*** (−0.969, −0.946)	−0.672*** (−0.680, −0.663)
cv_schemerepresentative	−0.530*** (−0.541, −0.520)	−0.636*** (−0.647, −0.625)	−0.833*** (−0.842, −0.824)
networkomnipath	−0.474*** (−0.483, −0.466)	−0.432*** (−0.441, −0.423)	−0.309*** (−0.316, −0.302)
diseaseAlzheimers disease	0.083*** (0.055, 0.110)	0.304*** (0.272, 0.335)	0.693*** (0.662, 0.723)
diseasearthritis	−0.430*** (−0.459, −0.401)	−0.297*** (−0.330, −0.263)	0.832*** (0.802, 0.862)
diseaseasthma	0.120*** (0.092, 0.148)	0.216*** (0.185, 0.248)	0.509*** (0.477, 0.540)
diseasebipolar disorder	0.156*** (0.128, 0.183)	0.511*** (0.480, 0.542)	1.024*** (0.995, 1.053)
diseasecardiac arrhythmia	0.548*** (0.521, 0.575)	0.952*** (0.922, 0.982)	1.429*** (1.401, 1.457)
diseaseCOPD	−0.080*** (−0.108, −0.051)	0.143*** (0.112, 0.175)	0.644*** (0.613, 0.675)
diseasecoronary heart disease	−0.169*** (−0.198, −0.141)	0.076*** (0.044, 0.108)	0.923*** (0.893, 0.953)
diseasedrug dependence	0.264*** (0.236, 0.292)	0.492*** (0.461, 0.523)	1.075*** (1.046, 1.104)
diseasehypertension	−0.165*** (−0.194, −0.137)	0.101*** (0.069, 0.133)	1.074*** (1.045, 1.103)
diseasemultiple sclerosis	0.211*** (0.183, 0.239)	0.580*** (0.550, 0.611)	1.288*** (1.260, 1.316)
diseaseobesity	0.042*** (0.015, 0.070)	0.301*** (0.270, 0.333)	1.186*** (1.157, 1.214)
diseaseParkinson's disease	0.581*** (0.553, 0.608)	0.842*** (0.812, 0.872)	1.322*** (1.294, 1.350)
diseasepsoriasis	−0.102*** (−0.130, −0.073)	−0.041** (−0.073, −0.008)	0.500*** (0.469, 0.532)
diseaserheumatoid arthritis	−0.353*** (−0.382, −0.324)	−0.236*** (−0.269, −0.203)	0.276*** (0.242, 0.309)
diseaseschizophrenia	0.238*** (0.211, 0.266)	0.506*** (0.476, 0.537)	1.141*** (1.112, 1.170)
diseasestroke	0.463*** (0.436, 0.491)	0.765*** (0.735, 0.795)	1.281*** (1.253, 1.309)
diseaselupus	−0.084*** (−0.112, −0.056)	0.205*** (0.173, 0.236)	0.601*** (0.570, 0.632)
diseasetype I diabetes	−0.103*** (−0.131, −0.075)	0.145*** (0.113, 0.177)	0.536*** (0.504, 0.567)
diseasetype II diabetes	−0.125*** (−0.153, −0.097)	0.138*** (0.106, 0.170)	0.827*** (0.797, 0.857)
diseaseulcerative colitis	0.088*** (0.060, 0.116)	0.173*** (0.141, 0.205)	0.030* (−0.006, 0.065)
diseaseunipolar depression	0.145*** (0.117, 0.173)	0.323*** (0.291, 0.354)	0.890*** (0.860, 0.920)
Observations	49,500	49,500	49,500
Note:			*p<0.1; **p<0.05; ***p<0.01

Table K: Predictions of the models DA4, DA5, DA6 (95% confidence intervals after averaging over disease).

Input: drugs data		STRING			OmniPath		
metric	method	classic	block	representative	classic	block	representative
	pr	(0.181, 0.188)	(0.088, 0.092)	(0.115, 0.120)	(0.121, 0.126)	(0.057, 0.059)	(0.075, 0.078)
	randomraw	(0.110, 0.116)	(0.051, 0.054)	(0.068, 0.072)	(0.072, 0.076)	(0.033, 0.034)	(0.043, 0.046)
	random	(0.084, 0.090)	(0.039, 0.041)	(0.051, 0.055)	(0.054, 0.058)	(0.024, 0.026)	(0.033, 0.035)
	EGAD	(0.441, 0.450)	(0.256, 0.263)	(0.317, 0.325)	(0.330, 0.337)	(0.176, 0.182)	(0.224, 0.230)
	ppr	(0.584, 0.592)	(0.379, 0.387)	(0.452, 0.460)	(0.466, 0.474)	(0.276, 0.282)	(0.339, 0.347)
	raw	(0.637, 0.644)	(0.433, 0.441)	(0.508, 0.516)	(0.522, 0.530)	(0.322, 0.329)	(0.391, 0.399)
	gm	(0.278, 0.286)	(0.144, 0.149)	(0.185, 0.191)	(0.193, 0.199)	(0.094, 0.098)	(0.123, 0.128)
partial_auroc_0.10	mc	(0.572, 0.580)	(0.368, 0.376)	(0.440, 0.449)	(0.454, 0.462)	(0.266, 0.273)	(0.329, 0.336)
	z	(0.612, 0.620)	(0.407, 0.415)	(0.481, 0.490)	(0.495, 0.504)	(0.300, 0.307)	(0.366, 0.374)
	knn	(0.590, 0.597)	(0.385, 0.393)	(0.458, 0.466)	(0.472, 0.480)	(0.280, 0.287)	(0.345, 0.352)
	wsld	(0.587, 0.595)	(0.382, 0.390)	(0.455, 0.463)	(0.469, 0.477)	(0.278, 0.285)	(0.342, 0.349)
	COSNet	(0.329, 0.338)	(0.176, 0.182)	(0.224, 0.231)	(0.234, 0.241)	(0.118, 0.122)	(0.152, 0.157)
	bagsvm	(0.625, 0.633)	(0.421, 0.429)	(0.495, 0.503)	(0.509, 0.517)	(0.311, 0.319)	(0.379, 0.387)
	rf	(0.622, 0.630)	(0.418, 0.426)	(0.492, 0.500)	(0.506, 0.514)	(0.309, 0.316)	(0.376, 0.384)
	svm	(0.563, 0.571)	(0.359, 0.367)	(0.431, 0.439)	(0.445, 0.453)	(0.259, 0.265)	(0.320, 0.328)
	pr	(0.111, 0.117)	(0.046, 0.048)	(0.062, 0.065)	(0.075, 0.079)	(0.030, 0.032)	(0.041, 0.043)
	randomraw	(0.054, 0.058)	(0.021, 0.023)	(0.029, 0.032)	(0.035, 0.038)	(0.014, 0.015)	(0.019, 0.021)
	random	(0.044, 0.047)	(0.017, 0.019)	(0.024, 0.026)	(0.029, 0.031)	(0.011, 0.012)	(0.015, 0.017)
	EGAD	(0.313, 0.322)	(0.149, 0.154)	(0.194, 0.201)	(0.228, 0.235)	(0.102, 0.106)	(0.135, 0.140)
	ppr	(0.459, 0.468)	(0.246, 0.253)	(0.310, 0.318)	(0.356, 0.364)	(0.175, 0.180)	(0.226, 0.232)
	raw	(0.512, 0.520)	(0.287, 0.294)	(0.357, 0.365)	(0.405, 0.413)	(0.207, 0.213)	(0.265, 0.271)
	gm	(0.256, 0.264)	(0.116, 0.121)	(0.154, 0.159)	(0.182, 0.189)	(0.079, 0.082)	(0.105, 0.110)
partial_auroc_0.05	mc	(0.411, 0.420)	(0.211, 0.218)	(0.270, 0.277)	(0.312, 0.320)	(0.148, 0.153)	(0.194, 0.199)
	z	(0.491, 0.500)	(0.270, 0.277)	(0.338, 0.346)	(0.385, 0.393)	(0.194, 0.199)	(0.249, 0.255)
	knn	(0.471, 0.480)	(0.255, 0.262)	(0.320, 0.328)	(0.366, 0.375)	(0.182, 0.187)	(0.234, 0.241)
	wsld	(0.466, 0.475)	(0.251, 0.258)	(0.316, 0.324)	(0.362, 0.370)	(0.179, 0.184)	(0.231, 0.237)
	COSNet	(0.282, 0.291)	(0.131, 0.136)	(0.172, 0.178)	(0.203, 0.210)	(0.089, 0.093)	(0.119, 0.123)
	bagsvm	(0.505, 0.513)	(0.281, 0.288)	(0.350, 0.358)	(0.398, 0.406)	(0.202, 0.208)	(0.259, 0.266)
	rf	(0.544, 0.552)	(0.314, 0.321)	(0.387, 0.395)	(0.436, 0.445)	(0.229, 0.235)	(0.290, 0.298)
	svm	(0.483, 0.492)	(0.264, 0.271)	(0.331, 0.338)	(0.377, 0.386)	(0.189, 0.194)	(0.243, 0.249)
	pr	(3.77, 3.98)	(1.93, 2.04)	(1.64, 1.73)	(2.77, 2.92)	(1.42, 1.49)	(1.20, 1.27)
	randomraw	(2.16, 2.32)	(1.10, 1.18)	(0.94, 1.01)	(1.59, 1.70)	(0.81, 0.87)	(0.69, 0.74)
	random	(1.70, 1.84)	(0.87, 0.94)	(0.74, 0.80)	(1.25, 1.35)	(0.64, 0.69)	(0.54, 0.59)
	EGAD	(13.14, 13.54)	(6.71, 6.93)	(5.71, 5.89)	(9.65, 9.95)	(4.93, 5.09)	(4.19, 4.33)
	ppr	(19.06, 19.55)	(9.73, 10.00)	(8.28, 8.51)	(14.00, 14.36)	(7.14, 7.35)	(6.08, 6.25)
	raw	(21.74, 22.27)	(11.10, 11.39)	(9.44, 9.69)	(15.96, 16.36)	(8.15, 8.37)	(6.93, 7.12)
	gm	(11.22, 11.59)	(5.73, 5.92)	(4.87, 5.04)	(8.24, 8.51)	(4.21, 4.35)	(3.58, 3.70)
top_100_hits	mc	(16.66, 17.11)	(8.50, 8.75)	(7.23, 7.45)	(12.23, 12.57)	(6.24, 6.43)	(5.31, 5.47)
	z	(20.89, 21.41)	(10.67, 10.95)	(9.07, 9.32)	(15.34, 15.73)	(7.83, 8.04)	(6.66, 6.85)
	knn	(20.59, 21.11)	(10.51, 10.79)	(8.94, 9.19)	(15.12, 15.51)	(7.72, 7.93)	(6.57, 6.75)
	wsld	(20.42, 20.94)	(10.43, 10.71)	(8.87, 9.11)	(15.00, 15.38)	(7.66, 7.86)	(6.51, 6.69)
	COSNet	(11.71, 12.08)	(5.98, 6.18)	(5.09, 5.26)	(8.60, 8.88)	(4.39, 4.54)	(3.73, 3.86)
	bagsvm	(21.47, 22.00)	(10.96, 11.25)	(9.32, 9.58)	(15.77, 16.16)	(8.05, 8.27)	(6.85, 7.03)
	rf	(24.06, 24.62)	(12.28, 12.59)	(10.45, 10.72)	(17.66, 18.09)	(9.02, 9.25)	(7.67, 7.87)
	svm	(21.18, 21.71)	(10.81, 11.10)	(9.20, 9.45)	(15.55, 15.95)	(7.94, 8.15)	(6.75, 6.94)

Reduced models

Table L: Models for the metrics auroc, auprc, top_20_hits using the drugs input, the STRING network and the block cross-validation strategy (model names rDA1, rDA2 and rDA3)

	auroc	auprc	top_20_hits
Constant	1.166*** (1.120, 1.212)	−5.030*** (−5.132, −4.928)	−2.853*** (−3.043, −2.664)
methodrandomraw	−0.451*** (−0.489, −0.414)	−0.282*** (−0.385, −0.180)	−1.452*** (−1.732, −1.172)
methodrandom	−0.986*** (−1.023, −0.949)	−0.720*** (−0.837, −0.604)	−0.676*** (−0.887, −0.466)
methodEGAD	0.191*** (0.151, 0.231)	1.112*** (1.032, 1.191)	1.697*** (1.564, 1.830)
methodppr	0.850*** (0.805, 0.895)	1.664*** (1.588, 1.740)	2.064*** (1.935, 2.194)
methodraw	0.821*** (0.776, 0.866)	1.856*** (1.781, 1.931)	2.249*** (2.121, 2.377)
methodgm	−0.938*** (−0.975, −0.901)	0.877*** (0.795, 0.958)	1.878*** (1.747, 2.009)
methodmc	0.674*** (0.631, 0.718)	1.046*** (0.966, 1.126)	1.083*** (0.942, 1.224)
methodz	0.682*** (0.638, 0.726)	1.712*** (1.636, 1.787)	2.136*** (2.007, 2.265)
methodknn	0.869*** (0.824, 0.915)	1.935*** (1.861, 2.010)	2.421*** (2.294, 2.549)
methodwsld	0.879*** (0.833, 0.924)	1.954*** (1.880, 2.029)	2.431*** (2.304, 2.558)
methodCOSNet	−1.351*** (−1.389, −1.314)	1.050*** (0.969, 1.130)	2.070*** (1.941, 2.200)
methodbagsvm	0.739*** (0.695, 0.783)	1.862*** (1.787, 1.937)	2.258*** (2.130, 2.387)
methodrf	0.613*** (0.570, 0.656)	2.229*** (2.156, 2.303)	2.740*** (2.614, 2.866)
methodsvm	0.251*** (0.210, 0.291)	1.941*** (1.867, 2.016)	2.538*** (2.412, 2.665)
diseaseAlzheimers disease	−0.080*** (−0.133, −0.027)	1.252*** (1.163, 1.341)	1.961*** (1.805, 2.117)
diseasearthritis	−0.350*** (−0.402, −0.299)	0.885*** (0.792, 0.977)	1.561*** (1.401, 1.722)
diseaseasthma	−0.037 (−0.090, 0.016)	0.638*** (0.542, 0.734)	0.885*** (0.712, 1.059)
diseasebipolar disorder	−0.253*** (−0.305, −0.201)	1.308*** (1.220, 1.397)	1.797*** (1.639, 1.954)
diseasecardiac arrhythmia	−0.143*** (−0.195, −0.090)	2.135*** (2.051, 2.218)	2.889*** (2.739, 3.039)
diseaseCOPD	−0.327*** (−0.379, −0.275)	0.656*** (0.560, 0.752)	0.866*** (0.692, 1.040)
diseasecoronary heart disease	−0.526*** (−0.577, −0.475)	0.905*** (0.813, 0.998)	1.501*** (1.339, 1.662)
diseasedrug dependence	−0.088*** (−0.141, −0.035)	1.391*** (1.304, 1.479)	2.056*** (1.901, 2.211)
diseasehypertension	−0.461*** (−0.513, −0.410)	0.868*** (0.775, 0.961)	1.526*** (1.365, 1.687)
diseasemultiple sclerosis	−0.159*** (−0.211, −0.106)	2.190*** (2.107, 2.274)	3.041*** (2.892, 3.190)
diseaseobesity	−0.411*** (−0.463, −0.360)	1.088*** (0.998, 1.179)	1.671*** (1.512, 1.830)
diseaseParkinson's disease	0.267*** (0.212, 0.322)	2.594*** (2.511, 2.676)	3.130*** (2.981, 3.279)
diseasepsoriasis	−0.042 (−0.095, 0.011)	1.205*** (1.115, 1.294)	1.841*** (1.684, 1.998)
diseaserheumatoid arthritis	−0.202*** (−0.254, −0.150)	0.630*** (0.534, 0.726)	1.120*** (0.952, 1.288)
diseaseschizophrenia	−0.245*** (−0.297, −0.192)	1.057*** (0.966, 1.148)	1.379*** (1.216, 1.542)
diseasestroke	0.136*** (0.082, 0.190)	2.395*** (2.312, 2.478)	3.064*** (2.915, 3.213)
diseaselupus	−0.488*** (−0.539, −0.437)	0.648*** (0.552, 0.744)	0.464*** (0.278, 0.650)
diseasetype I diabetes	−0.369*** (−0.420, −0.317)	0.602*** (0.505, 0.698)	0.641*** (0.461, 0.821)
diseasetype II diabetes	−0.353*** (−0.404, −0.301)	0.947*** (0.855, 1.039)	1.395*** (1.232, 1.558)
diseaseulcerative colitis	0.192*** (0.137, 0.247)	0.485*** (0.387, 0.584)	0.809*** (0.634, 0.985)
diseaseunipolar depression	−0.030 (−0.083, 0.023)	1.148*** (1.058, 1.238)	1.772*** (1.614, 1.929)
Observations	8,250	8,250	8,250
Note:			*p<0.1; **p<0.05; ***p<0.01

Table M: Models for the metrics partial_auroc_0.10, partial_auroc_0.05, top_100_hits using the drugs input, the STRING network and the block cross-validation strategy (model names rDA4, rDA5 and rDA6)

	partial_auroc_0.10	partial_auroc_0.05	top_100_hits
Constant	−2.106*** (−2.172, −2.039)	−3.168*** (−3.253, −3.084)	−0.339*** (−0.438, −0.241)
methodrandomraw	−0.663*** (−0.748, −0.579)	−0.791*** (−0.908, −0.673)	−0.640*** (−0.759, −0.521)
methodrandom	−0.812*** (−0.900, −0.724)	−0.832*** (−0.951, −0.713)	−0.623*** (−0.741, −0.505)
methodEGAD	1.369*** (1.307, 1.431)	1.291*** (1.212, 1.370)	1.243*** (1.163, 1.322)
methodppr	1.837*** (1.777, 1.898)	1.866*** (1.790, 1.942)	1.626*** (1.550, 1.703)
methodraw	1.868*** (1.807, 1.928)	1.919*** (1.843, 1.995)	1.730*** (1.654, 1.806)
methodgm	−0.332*** (−0.410, −0.254)	0.289*** (0.199, 0.379)	0.556*** (0.469, 0.644)
methodmc	1.590*** (1.529, 1.651)	1.527*** (1.450, 1.605)	1.387*** (1.309, 1.465)
methodz	1.769*** (1.708, 1.829)	1.844*** (1.768, 1.920)	1.653*** (1.577, 1.730)
methodknn	1.923*** (1.862, 1.984)	2.062*** (1.987, 2.138)	1.913*** (1.838, 1.988)
methodwsld	1.936*** (1.875, 1.996)	2.082*** (2.007, 2.158)	1.962*** (1.888, 2.037)
methodCOSNet	0.192*** (0.121, 0.262)	0.700*** (0.616, 0.784)	0.836*** (0.752, 0.920)
methodbagsvm	1.852*** (1.792, 1.913)	1.914*** (1.839, 1.990)	1.724*** (1.649, 1.800)
methodrf	1.991*** (1.931, 2.052)	2.324*** (2.249, 2.399)	2.016*** (1.942, 2.090)
methodsvm	1.705*** (1.645, 1.766)	2.060*** (1.985, 2.136)	1.874*** (1.799, 1.949)
diseaseAlzheimers disease	0.066** (0.005, 0.128)	0.482*** (0.412, 0.551)	0.999*** (0.917, 1.081)
diseasearthritis	−0.627*** (−0.693, −0.562)	−0.353*** (−0.431, −0.275)	0.949*** (0.866, 1.031)
diseaseasthma	0.158*** (0.096, 0.219)	0.312*** (0.242, 0.383)	0.598*** (0.510, 0.685)
diseasebipolar disorder	−0.040 (−0.101, 0.022)	0.365*** (0.295, 0.436)	1.193*** (1.113, 1.273)
diseasecardiac arrhythmia	0.245*** (0.184, 0.306)	0.869*** (0.802, 0.937)	1.705*** (1.628, 1.781)
diseaseCOPD	−0.388*** (−0.452, −0.325)	−0.189*** (−0.264, −0.113)	0.552*** (0.464, 0.640)
diseasecoronary heart disease	−0.532*** (−0.597, −0.467)	−0.162*** (−0.238, −0.087)	0.900*** (0.817, 0.983)
diseasedrug dependence	0.205*** (0.144, 0.266)	0.553*** (0.484, 0.622)	1.347*** (1.268, 1.426)
diseasehypertension	−0.655*** (−0.721, −0.589)	−0.273*** (−0.350, −0.196)	0.964*** (0.882, 1.047)
diseasemultiple sclerosis	0.099*** (0.038, 0.160)	0.689*** (0.621, 0.757)	1.561*** (1.483, 1.638)
diseaseobesity	−0.385*** (−0.449, −0.321)	−0.062 (−0.136, 0.012)	1.125*** (1.045, 1.206)
diseaseParkinson's disease	0.832*** (0.772, 0.893)	1.302*** (1.236, 1.368)	1.785*** (1.709, 1.861)
diseasepsoriasis	−0.002 (−0.064, 0.060)	0.281*** (0.210, 0.352)	0.841*** (0.757, 0.925)
diseaserheumatoid arthritis	−0.255*** (−0.318, −0.192)	0.097*** (0.025, 0.170)	0.652*** (0.566, 0.739)
diseaseschizophrenia	−0.205*** (−0.267, −0.142)	0.102*** (0.030, 0.175)	1.146*** (1.065, 1.226)
diseasestroke	0.519*** (0.458, 0.579)	0.966*** (0.899, 1.033)	1.661*** (1.584, 1.737)
diseaselupus	−0.619*** (−0.684, −0.553)	−0.348*** (−0.426, −0.270)	0.378*** (0.287, 0.469)
diseasetype I diabetes	−0.640*** (−0.706, −0.574)	−0.457*** (−0.537, −0.377)	0.405*** (0.315, 0.496)
diseasetype II diabetes	−0.442*** (−0.506, −0.377)	−0.122*** (−0.197, −0.047)	0.854*** (0.770, 0.937)
diseaseulcerative colitis	0.371*** (0.310, 0.432)	0.616*** (0.547, 0.685)	0.432*** (0.342, 0.523)
diseaseunipolar depression	0.079** (0.018, 0.141)	0.385*** (0.315, 0.456)	1.060*** (0.978, 1.141)
Observations	8,250	8,250	8,250
Note:			*p<0.1; **p<0.05; ***p<0.01

Table N: Predictions of the models rDA1, rDA2, rDA3, rDA4, rDA5, rDA6 (95% confidence intervals after averaging over disease). These models are adjusted on the drugs data, STRING network and block cross-validation, so the only independent variables are the method and the disease.

method	auroc	partial_auroc_0.10	partial_auroc_0.05	auprc	top_20_hits	top_100_hits
pr	(0.723, 0.734)	(0.095, 0.104)	(0.047, 0.054)	(0.019, 0.021)	(0.25, 0.33)	(1.73, 1.99)
randomraw	(0.624, 0.636)	(0.050, 0.057)	(0.021, 0.026)	(0.014, 0.016)	(0.05, 0.09)	(0.89, 1.08)
random	(0.494, 0.506)	(0.044, 0.050)	(0.020, 0.025)	(0.009, 0.011)	(0.12, 0.17)	(0.91, 1.10)
EGAD	(0.759, 0.770)	(0.295, 0.309)	(0.156, 0.167)	(0.056, 0.061)	(1.49, 1.66)	(6.20, 6.69)
ppr	(0.858, 0.867)	(0.401, 0.417)	(0.248, 0.262)	(0.094, 0.100)	(2.17, 2.37)	(9.16, 9.75)
raw	(0.855, 0.863)	(0.408, 0.424)	(0.258, 0.272)	(0.112, 0.119)	(2.62, 2.84)	(10.18, 10.80)
gm	(0.506, 0.518)	(0.069, 0.077)	(0.062, 0.070)	(0.045, 0.049)	(1.79, 1.98)	(3.07, 3.42)
mc	(0.836, 0.845)	(0.343, 0.358)	(0.190, 0.202)	(0.053, 0.057)	(0.79, 0.91)	(7.18, 7.70)
z	(0.837, 0.846)	(0.385, 0.400)	(0.244, 0.258)	(0.098, 0.105)	(2.33, 2.54)	(9.42, 10.02)
knn	(0.860, 0.869)	(0.422, 0.438)	(0.287, 0.301)	(0.120, 0.127)	(3.12, 3.37)	(12.25, 12.94)
wsld	(0.862, 0.870)	(0.425, 0.441)	(0.291, 0.305)	(0.122, 0.129)	(3.15, 3.40)	(12.88, 13.58)
COSNet	(0.404, 0.416)	(0.113, 0.123)	(0.092, 0.101)	(0.053, 0.057)	(2.18, 2.39)	(4.10, 4.49)
bagsvm	(0.844, 0.853)	(0.405, 0.420)	(0.257, 0.271)	(0.113, 0.119)	(2.64, 2.87)	(10.12, 10.74)
rf	(0.827, 0.836)	(0.439, 0.454)	(0.344, 0.359)	(0.156, 0.163)	(4.31, 4.61)	(13.60, 14.32)
svm	(0.770, 0.780)	(0.370, 0.385)	(0.287, 0.301)	(0.121, 0.128)	(3.51, 3.78)	(11.78, 12.45)

Genetic input

Table O: Models for the metrics auroc, auprc, top_20_hits using the genetic input (model names GA1, GA2 and GA3)

	auroc	auprc	top_20_hits
Constant	0.793*** (0.776, 0.811)	−4.383*** (−4.423, −4.344)	−1.676*** (−1.776, −1.576)
methodrandomraw	−0.337*** (−0.352, −0.322)	−0.305*** (−0.333, −0.277)	−1.069*** (−1.145, −0.993)
methodrandom	−0.759*** (−0.774, −0.744)	−0.676*** (−0.707, −0.645)	−0.981*** (−1.055, −0.908)
methodEGAD	−0.392*** (−0.407, −0.377)	−0.264*** (−0.292, −0.236)	−0.860*** (−0.931, −0.789)
methodppr	−0.001 (−0.016, 0.015)	0.196*** (0.171, 0.221)	0.393*** (0.343, 0.443)
methodraw	−0.129*** (−0.145, −0.114)	0.102*** (0.077, 0.128)	0.135*** (0.082, 0.187)
methodgm	−1.249*** (−1.265, −1.234)	−0.479*** (−0.509, −0.449)	−0.355*** (−0.415, −0.295)
methodmc	−0.310*** (−0.325, −0.295)	0.005 (−0.021, 0.031)	0.403*** (0.353, 0.453)
methodz	−0.330*** (−0.345, −0.314)	0.042*** (0.017, 0.068)	−0.071** (−0.127, −0.016)
methodknn	−0.242*** (−0.257, −0.227)	−0.019 (−0.045, 0.007)	0.103*** (0.050, 0.156)
methodwsld	−0.243*** (−0.258, −0.228)	−0.060*** (−0.087, −0.034)	−0.055* (−0.110, 0.0003)
methodCOSNet	−0.692*** (−0.707, −0.677)	−0.067*** (−0.094, −0.041)	−0.052* (−0.108, 0.003)
methodbagsvm	−0.356*** (−0.371, −0.341)	−0.013 (−0.039, 0.013)	0.106*** (0.053, 0.159)
methodrf	−0.497*** (−0.512, −0.482)	−0.455*** (−0.484, −0.425)	−0.624*** (−0.689, −0.559)
methodsvm	−0.629*** (−0.644, −0.614)	−0.420*** (−0.449, −0.391)	−0.565*** (−0.629, −0.501)
cv_schemeblock	0.045*** (0.038, 0.051)	0.018*** (0.006, 0.030)	−0.172*** (−0.196, −0.147)
cv_schemerepresentative	0.118*** (0.111, 0.125)	−0.328*** (−0.341, −0.315)	−0.473*** (−0.500, −0.446)
diseaseAlzheimers disease	0.082*** (0.064, 0.101)	0.686*** (0.644, 0.729)	1.262*** (1.158, 1.366)
diseasearthritis	0.051*** (0.033, 0.070)	0.938*** (0.898, 0.979)	1.161*** (1.056, 1.266)
diseaseasthma	0.248*** (0.230, 0.267)	0.619*** (0.576, 0.662)	0.516*** (0.401, 0.632)
diseasebipolar disorder	−0.082*** (−0.100, −0.064)	0.587*** (0.544, 0.630)	0.424*** (0.306, 0.542)
diseasecardiac arrhythmia	−0.075*** (−0.093, −0.057)	1.625*** (1.587, 1.662)	2.476*** (2.381, 2.571)
diseaseCOPD	−0.111*** (−0.129, −0.093)	0.584*** (0.541, 0.627)	1.255*** (1.152, 1.359)
diseasecoronary heart disease	−0.243*** (−0.262, −0.225)	0.663*** (0.620, 0.705)	1.261*** (1.158, 1.365)
diseasedrug dependence	−0.036*** (−0.054, −0.018)	0.835*** (0.793, 0.876)	1.597*** (1.497, 1.698)
diseasehypertension	−0.083*** (−0.101, −0.065)	0.995*** (0.955, 1.035)	1.789*** (1.690, 1.888)
diseasemultiple sclerosis	0.041*** (0.023, 0.059)	0.952*** (0.912, 0.993)	1.080*** (0.974, 1.186)
diseaseobesity	−0.070*** (−0.088, −0.052)	0.937*** (0.897, 0.978)	1.147*** (1.042, 1.252)
diseaseParkinson's disease	−0.222*** (−0.240, −0.204)	0.418*** (0.374, 0.462)	0.213*** (0.090, 0.337)
diseasepsoriasis	0.154*** (0.136, 0.173)	0.831*** (0.790, 0.872)	1.494*** (1.393, 1.596)
diseaserheumatoid arthritis	0.183*** (0.165, 0.202)	0.643*** (0.601, 0.686)	1.140*** (1.035, 1.245)
diseaseschizophrenia	0.028*** (0.010, 0.046)	0.748*** (0.706, 0.790)	0.613*** (0.499, 0.727)
diseasestroke	−0.295*** (−0.314, −0.277)	0.427*** (0.383, 0.471)	0.443*** (0.325, 0.560)
diseaselupus	−0.144*** (−0.162, −0.126)	0.416*** (0.372, 0.460)	1.084*** (0.978, 1.190)
diseasetype I diabetes mellitus	−0.023** (−0.041, −0.005)	0.516*** (0.473, 0.559)	1.072*** (0.966, 1.179)
diseasetype II diabetes mellitus	−0.176*** (−0.194, −0.158)	0.494*** (0.450, 0.537)	0.448*** (0.330, 0.565)
diseaseulcerative colitis	0.620*** (0.601, 0.639)	0.829*** (0.788, 0.870)	1.311*** (1.208, 1.414)
diseaseunipolar depression	0.125*** (0.107, 0.143)	0.851*** (0.810, 0.892)	1.382*** (1.280, 1.485)
networkomnipath	−0.167*** (−0.172, −0.161)	−0.052*** (−0.062, −0.041)	0.202*** (0.181, 0.224)
Observations	49,500	49,500	49,500
Note:			*p<0.1; **p<0.05; ***p<0.01

Table P: Predictions of the models GA1, GA2, GA3 (95% confidence intervals after averaging over disease).

Input: genetic data		STRING			OmniPath		
metric	method	classic	block	representative	classic	block	representative
	pr	(0.686, 0.691)	(0.695, 0.700)	(0.710, 0.715)	(0.649, 0.654)	(0.659, 0.664)	(0.675, 0.680)
	randomraw	(0.609, 0.615)	(0.620, 0.625)	(0.637, 0.642)	(0.569, 0.574)	(0.580, 0.585)	(0.597, 0.603)
	random	(0.505, 0.511)	(0.517, 0.522)	(0.535, 0.541)	(0.464, 0.469)	(0.475, 0.481)	(0.493, 0.499)
	EGAD	(0.596, 0.601)	(0.607, 0.612)	(0.624, 0.629)	(0.555, 0.561)	(0.566, 0.572)	(0.584, 0.590)
	ppr	(0.685, 0.691)	(0.695, 0.700)	(0.710, 0.715)	(0.648, 0.654)	(0.659, 0.664)	(0.675, 0.680)
	raw	(0.657, 0.662)	(0.667, 0.672)	(0.683, 0.688)	(0.619, 0.624)	(0.629, 0.635)	(0.646, 0.651)
	gm	(0.385, 0.390)	(0.395, 0.401)	(0.413, 0.419)	(0.346, 0.351)	(0.356, 0.362)	(0.373, 0.379)
auroc	mc	(0.615, 0.621)	(0.626, 0.631)	(0.643, 0.648)	(0.575, 0.581)	(0.586, 0.592)	(0.604, 0.609)
	z	(0.611, 0.616)	(0.621, 0.627)	(0.638, 0.644)	(0.570, 0.576)	(0.581, 0.587)	(0.599, 0.605)
	knn	(0.631, 0.637)	(0.642, 0.647)	(0.658, 0.664)	(0.592, 0.597)	(0.602, 0.608)	(0.620, 0.625)
	wsld	(0.631, 0.637)	(0.641, 0.647)	(0.658, 0.663)	(0.591, 0.597)	(0.602, 0.608)	(0.620, 0.625)
	COSNet	(0.522, 0.528)	(0.533, 0.539)	(0.551, 0.557)	(0.480, 0.486)	(0.492, 0.497)	(0.510, 0.516)
	bagsvm	(0.605, 0.610)	(0.615, 0.621)	(0.632, 0.638)	(0.564, 0.570)	(0.575, 0.581)	(0.593, 0.598)
	rf	(0.570, 0.576)	(0.581, 0.587)	(0.599, 0.605)	(0.529, 0.535)	(0.540, 0.546)	(0.558, 0.564)
	svm	(0.538, 0.544)	(0.549, 0.555)	(0.567, 0.573)	(0.496, 0.502)	(0.507, 0.513)	(0.526, 0.531)
	pr	(0.024, 0.025)	(0.025, 0.026)	(0.018, 0.018)	(0.023, 0.024)	(0.023, 0.024)	(0.017, 0.017)
	randomraw	(0.018, 0.019)	(0.018, 0.019)	(0.013, 0.014)	(0.017, 0.018)	(0.017, 0.018)	(0.012, 0.013)
	random	(0.012, 0.013)	(0.013, 0.013)	(0.009, 0.009)	(0.012, 0.012)	(0.012, 0.013)	(0.009, 0.009)
	EGAD	(0.019, 0.020)	(0.019, 0.020)	(0.014, 0.014)	(0.018, 0.019)	(0.018, 0.019)	(0.013, 0.013)
	ppr	(0.029, 0.030)	(0.030, 0.031)	(0.021, 0.022)	(0.028, 0.029)	(0.028, 0.030)	(0.020, 0.021)
	raw	(0.027, 0.028)	(0.027, 0.028)	(0.019, 0.020)	(0.026, 0.026)	(0.026, 0.027)	(0.018, 0.019)
	gm	(0.015, 0.016)	(0.015, 0.016)	(0.011, 0.011)	(0.014, 0.015)	(0.015, 0.015)	(0.010, 0.011)
auprc	mc	(0.024, 0.025)	(0.025, 0.026)	(0.018, 0.018)	(0.023, 0.024)	(0.024, 0.025)	(0.017, 0.017)
	z	(0.025, 0.026)	(0.026, 0.027)	(0.018, 0.019)	(0.024, 0.025)	(0.024, 0.025)	(0.017, 0.018)
	knn	(0.024, 0.025)	(0.024, 0.025)	(0.017, 0.018)	(0.023, 0.024)	(0.023, 0.024)	(0.016, 0.017)
	wsld	(0.023, 0.024)	(0.023, 0.024)	(0.017, 0.017)	(0.022, 0.023)	(0.022, 0.023)	(0.016, 0.016)
	COSNet	(0.023, 0.024)	(0.023, 0.024)	(0.016, 0.017)	(0.022, 0.022)	(0.022, 0.023)	(0.016, 0.016)
	bagsvm	(0.024, 0.025)	(0.024, 0.025)	(0.017, 0.018)	(0.023, 0.024)	(0.023, 0.024)	(0.016, 0.017)
	rf	(0.015, 0.016)	(0.016, 0.017)	(0.011, 0.012)	(0.015, 0.015)	(0.015, 0.016)	(0.011, 0.011)
	svm	(0.016, 0.017)	(0.016, 0.017)	(0.012, 0.012)	(0.015, 0.016)	(0.015, 0.016)	(0.011, 0.012)
	pr	(0.51, 0.56)	(0.43, 0.47)	(0.32, 0.35)	(0.63, 0.68)	(0.53, 0.58)	(0.39, 0.43)
	randomraw	(0.17, 0.20)	(0.14, 0.17)	(0.11, 0.12)	(0.21, 0.24)	(0.18, 0.20)	(0.13, 0.15)
	random	(0.19, 0.21)	(0.16, 0.18)	(0.12, 0.13)	(0.23, 0.26)	(0.19, 0.22)	(0.14, 0.16)
	EGAD	(0.21, 0.24)	(0.18, 0.20)	(0.13, 0.15)	(0.26, 0.30)	(0.22, 0.25)	(0.16, 0.18)
	ppr	(0.77, 0.82)	(0.64, 0.69)	(0.48, 0.51)	(0.94, 1.01)	(0.79, 0.85)	(0.58, 0.63)
	raw	(0.59, 0.64)	(0.50, 0.54)	(0.37, 0.40)	(0.72, 0.78)	(0.61, 0.66)	(0.45, 0.49)
	gm	(0.36, 0.40)	(0.30, 0.33)	(0.22, 0.25)	(0.44, 0.48)	(0.37, 0.41)	(0.27, 0.30)
top_20_hits	mc	(0.77, 0.83)	(0.65, 0.70)	(0.48, 0.52)	(0.95, 1.02)	(0.80, 0.86)	(0.59, 0.64)
	z	(0.48, 0.52)	(0.40, 0.44)	(0.30, 0.33)	(0.59, 0.64)	(0.49, 0.54)	(0.36, 0.40)
	knn	(0.57, 0.62)	(0.48, 0.52)	(0.35, 0.39)	(0.70, 0.76)	(0.59, 0.64)	(0.43, 0.47)
	wsld	(0.49, 0.53)	(0.41, 0.45)	(0.30, 0.33)	(0.60, 0.65)	(0.50, 0.55)	(0.37, 0.40)
	COSNet	(0.49, 0.53)	(0.41, 0.45)	(0.30, 0.33)	(0.60, 0.65)	(0.50, 0.55)	(0.37, 0.41)
	bagsvm	(0.57, 0.62)	(0.48, 0.52)	(0.36, 0.39)	(0.70, 0.76)	(0.59, 0.64)	(0.44, 0.47)
	rf	(0.27, 0.30)	(0.23, 0.26)	(0.17, 0.19)	(0.33, 0.37)	(0.28, 0.31)	(0.21, 0.23)
	svm	(0.29, 0.32)	(0.24, 0.27)	(0.18, 0.20)	(0.35, 0.39)	(0.30, 0.33)	(0.22, 0.25)

Table Q: Models for the metrics partial_auroc_0.10, partial_auroc_0.05, top_100_hits using the genetic input (model names GA4, GA5 and GA6)

	partial_auroc_0.10	partial_auroc_0.05	top_100_hits
Constant	−2.173*** (−2.207, −2.138)	−2.758*** (−2.801, −2.715)	0.249*** (0.195, 0.302)
methodrandomraw	−0.622*** (−0.654, −0.589)	−0.782*** (−0.824, −0.740)	−0.514*** (−0.555, −0.473)
methodrandom	−0.871*** (−0.905, −0.836)	−0.999*** (−1.044, −0.954)	−0.784*** (−0.828, −0.739)
methodEGAD	−0.040*** (−0.069, −0.012)	−0.349*** (−0.386, −0.312)	−0.267*** (−0.305, −0.229)
methodppr	0.395*** (0.369, 0.421)	0.398*** (0.366, 0.430)	0.377*** (0.345, 0.409)
methodraw	0.170*** (0.143, 0.197)	0.030* (−0.004, 0.065)	0.085*** (0.051, 0.120)
methodgm	−1.096*** (−1.133, −1.059)	−0.931*** (−0.976, −0.887)	−0.553*** (−0.595, −0.512)
methodmc	0.127*** (0.099, 0.154)	0.163*** (0.130, 0.197)	0.196*** (0.162, 0.230)
methodz	0.141*** (0.114, 0.169)	0.112*** (0.078, 0.145)	0.162*** (0.128, 0.196)
methodknn	−0.188*** (−0.217, −0.159)	−0.244*** (−0.280, −0.207)	−0.106*** (−0.142, −0.070)
methodwsld	−0.233*** (−0.263, −0.204)	−0.271*** (−0.308, −0.234)	−0.122*** (−0.158, −0.085)
methodCOSNet	0.009 (−0.019, 0.037)	0.090*** (0.056, 0.123)	0.103*** (0.069, 0.138)
methodbagsvm	0.013 (−0.015, 0.041)	−0.070*** (−0.105, −0.035)	−0.004 (−0.040, 0.031)
methodrf	−0.473*** (−0.504, −0.442)	−0.630*** (−0.670, −0.590)	−0.476*** (−0.516, −0.436)
methodsvm	−0.475*** (−0.507, −0.444)	−0.569*** (−0.608, −0.529)	−0.465*** (−0.505, −0.424)
cv_schemeblock	−0.024*** (−0.038, −0.011)	−0.043*** (−0.060, −0.026)	−0.087*** (−0.103, −0.071)
cv_schemerepresentative	0.005 (−0.008, 0.019)	−0.028*** (−0.044, −0.011)	−0.433*** (−0.451, −0.416)
diseaseAlzheimers disease	0.174*** (0.137, 0.211)	0.255*** (0.209, 0.301)	0.739*** (0.683, 0.796)
diseasearthritis	−0.020 (−0.059, 0.019)	−0.102*** (−0.152, −0.052)	0.930*** (0.875, 0.985)
diseaseasthma	0.339*** (0.303, 0.375)	0.073*** (0.025, 0.121)	0.350*** (0.290, 0.411)
diseasebipolar disorder	−0.075*** (−0.114, −0.036)	−0.307*** (−0.360, −0.254)	0.327*** (0.266, 0.388)
diseasecardiac arrhythmia	0.684*** (0.650, 0.718)	1.035*** (0.994, 1.077)	1.750*** (1.700, 1.801)
diseaseCOPD	−0.073*** (−0.112, −0.034)	−0.140*** (−0.191, −0.090)	0.382*** (0.322, 0.442)
diseasecoronary heart disease	−0.160*** (−0.199, −0.120)	−0.134*** (−0.184, −0.084)	0.701*** (0.644, 0.758)
diseasedrug dependence	0.048** (0.010, 0.086)	0.070*** (0.022, 0.118)	0.759*** (0.702, 0.815)
diseasehypertension	0.148*** (0.110, 0.185)	0.196*** (0.149, 0.243)	1.104*** (1.051, 1.158)
diseasemultiple sclerosis	0.126*** (0.088, 0.164)	−0.039 (−0.089, 0.010)	0.865*** (0.810, 0.921)
diseaseobesity	0.144*** (0.107, 0.182)	0.064*** (0.016, 0.113)	0.988*** (0.934, 1.043)
diseaseParkinson's disease	−0.744*** (−0.791, −0.698)	−0.811*** (−0.872, −0.750)	0.057* (−0.008, 0.121)
diseasepsoriasis	0.407*** (0.371, 0.443)	0.435*** (0.391, 0.480)	0.925*** (0.870, 0.980)
diseaserheumatoid arthritis	0.402*** (0.366, 0.438)	0.385*** (0.340, 0.430)	0.772*** (0.716, 0.828)
diseaseschizophrenia	−0.022 (−0.061, 0.016)	−0.136*** (−0.187, −0.086)	0.700*** (0.643, 0.757)
diseasestroke	−0.509*** (−0.552, −0.465)	−0.535*** (−0.591, −0.479)	0.334*** (0.273, 0.395)
diseaselupus	0.177*** (0.140, 0.214)	0.261*** (0.215, 0.307)	0.558*** (0.499, 0.616)
diseasetype I diabetes mellitus	0.424*** (0.388, 0.459)	0.423*** (0.378, 0.468)	0.658*** (0.601, 0.715)
diseasetype II diabetes mellitus	−0.274*** (−0.315, −0.233)	−0.381*** (−0.434, −0.327)	0.314*** (0.252, 0.375)
diseaseulcerative colitis	1.008*** (0.974, 1.041)	0.977*** (0.936, 1.019)	0.744*** (0.687, 0.800)
diseaseunipolar depression	0.334*** (0.298, 0.370)	0.320*** (0.275, 0.366)	0.943*** (0.888, 0.997)
networkomnipath	−0.145*** (−0.156, −0.134)	−0.105*** (−0.119, −0.091)	0.013* (−0.001, 0.026)
Observations	49,500	49,500	49,500
Note:			*p<0.1; **p<0.05; ***p<0.01

Table R: Predictions of the models GA4, GA5 and GA6 (95% confidence intervals after averaging over disease).

Input: genetic data		STRING			OmniPath		
metric	method	classic	block	representative	classic	block	representative
partial_auroc_0.10	pr	(0.111, 0.116)	(0.109, 0.113)	(0.112, 0.116)	(0.098, 0.102)	(0.095, 0.099)	(0.098, 0.102)
	randomraw	(0.063, 0.066)	(0.061, 0.064)	(0.063, 0.066)	(0.055, 0.058)	(0.053, 0.056)	(0.055, 0.058)
	random	(0.049, 0.052)	(0.048, 0.051)	(0.050, 0.053)	(0.043, 0.046)	(0.042, 0.044)	(0.043, 0.046)
	EGAD	(0.107, 0.112)	(0.105, 0.109)	(0.108, 0.112)	(0.094, 0.098)	(0.092, 0.096)	(0.095, 0.098)
	ppr	(0.157, 0.162)	(0.154, 0.159)	(0.158, 0.163)	(0.139, 0.143)	(0.136, 0.140)	(0.139, 0.144)
	raw	(0.129, 0.134)	(0.126, 0.131)	(0.130, 0.135)	(0.114, 0.118)	(0.111, 0.116)	(0.114, 0.119)
	gm	(0.040, 0.042)	(0.039, 0.041)	(0.040, 0.042)	(0.035, 0.037)	(0.034, 0.036)	(0.035, 0.037)
	mc	(0.124, 0.129)	(0.122, 0.126)	(0.125, 0.130)	(0.109, 0.114)	(0.107, 0.111)	(0.110, 0.114)
	z	(0.126, 0.131)	(0.123, 0.128)	(0.127, 0.131)	(0.111, 0.115)	(0.108, 0.113)	(0.111, 0.116)
	knn	(0.094, 0.098)	(0.092, 0.096)	(0.094, 0.098)	(0.082, 0.086)	(0.080, 0.084)	(0.083, 0.086)
	wsld	(0.090, 0.094)	(0.088, 0.092)	(0.090, 0.094)	(0.079, 0.082)	(0.077, 0.080)	(0.079, 0.083)
	COSNet	(0.112, 0.116)	(0.110, 0.114)	(0.113, 0.117)	(0.098, 0.102)	(0.096, 0.100)	(0.099, 0.103)
	bagsvm	(0.112, 0.117)	(0.110, 0.114)	(0.113, 0.117)	(0.099, 0.103)	(0.097, 0.101)	(0.099, 0.103)
	rf	(0.072, 0.076)	(0.070, 0.074)	(0.072, 0.076)	(0.063, 0.066)	(0.061, 0.065)	(0.063, 0.066)
	svm	(0.072, 0.075)	(0.070, 0.074)	(0.072, 0.076)	(0.063, 0.066)	(0.061, 0.064)	(0.063, 0.066)
partial_auroc_0.05	pr	(0.063, 0.066)	(0.061, 0.064)	(0.061, 0.065)	(0.057, 0.060)	(0.055, 0.058)	(0.056, 0.059)
	randomraw	(0.030, 0.032)	(0.028, 0.030)	(0.029, 0.031)	(0.027, 0.029)	(0.026, 0.028)	(0.026, 0.028)
	random	(0.024, 0.026)	(0.023, 0.025)	(0.023, 0.025)	(0.022, 0.023)	(0.021, 0.022)	(0.021, 0.023)
	EGAD	(0.045, 0.048)	(0.043, 0.046)	(0.044, 0.047)	(0.041, 0.043)	(0.039, 0.042)	(0.040, 0.042)
	ppr	(0.091, 0.095)	(0.088, 0.092)	(0.089, 0.093)	(0.083, 0.087)	(0.080, 0.083)	(0.081, 0.085)
	raw	(0.065, 0.068)	(0.062, 0.066)	(0.063, 0.067)	(0.059, 0.062)	(0.056, 0.059)	(0.057, 0.060)
	gm	(0.026, 0.028)	(0.024, 0.026)	(0.025, 0.027)	(0.023, 0.025)	(0.022, 0.024)	(0.022, 0.024)
	mc	(0.074, 0.077)	(0.071, 0.074)	(0.072, 0.075)	(0.067, 0.070)	(0.064, 0.067)	(0.065, 0.068)
	z	(0.070, 0.074)	(0.067, 0.071)	(0.068, 0.072)	(0.064, 0.067)	(0.061, 0.064)	(0.062, 0.065)
	knn	(0.050, 0.053)	(0.048, 0.051)	(0.049, 0.052)	(0.045, 0.048)	(0.043, 0.046)	(0.044, 0.047)
	wsld	(0.049, 0.052)	(0.047, 0.049)	(0.047, 0.050)	(0.044, 0.047)	(0.042, 0.045)	(0.043, 0.045)
	COSNet	(0.069, 0.072)	(0.066, 0.069)	(0.067, 0.070)	(0.062, 0.065)	(0.060, 0.063)	(0.061, 0.064)
	bagsvm	(0.059, 0.062)	(0.057, 0.060)	(0.057, 0.061)	(0.053, 0.056)	(0.051, 0.054)	(0.052, 0.055)
	rf	(0.034, 0.037)	(0.033, 0.035)	(0.033, 0.036)	(0.031, 0.033)	(0.030, 0.032)	(0.030, 0.032)
	svm	(0.036, 0.039)	(0.035, 0.037)	(0.036, 0.038)	(0.033, 0.035)	(0.032, 0.034)	(0.032, 0.034)
top_100_hits	pr	(2.46, 2.59)	(2.25, 2.38)	(1.59, 1.68)	(2.49, 2.63)	(2.28, 2.41)	(1.61, 1.71)
	randomraw	(1.46, 1.56)	(1.34, 1.43)	(0.95, 1.01)	(1.48, 1.58)	(1.35, 1.45)	(0.96, 1.03)
	random	(1.11, 1.20)	(1.02, 1.10)	(0.72, 0.78)	(1.12, 1.21)	(1.03, 1.11)	(0.73, 0.79)
	EGAD	(1.87, 1.99)	(1.72, 1.83)	(1.21, 1.29)	(1.90, 2.02)	(1.74, 1.85)	(1.23, 1.31)
	ppr	(3.59, 3.77)	(3.29, 3.45)	(2.33, 2.45)	(3.64, 3.81)	(3.34, 3.50)	(2.36, 2.48)
	raw	(2.68, 2.82)	(2.45, 2.59)	(1.73, 1.83)	(2.71, 2.86)	(2.48, 2.62)	(1.76, 1.86)
	gm	(1.40, 1.50)	(1.28, 1.38)	(0.91, 0.98)	(1.42, 1.52)	(1.30, 1.40)	(0.92, 0.99)
	mc	(2.99, 3.15)	(2.74, 2.89)	(1.94, 2.04)	(3.03, 3.19)	(2.78, 2.92)	(1.96, 2.07)
	z	(2.89, 3.04)	(2.65, 2.79)	(1.87, 1.98)	(2.93, 3.08)	(2.68, 2.83)	(1.90, 2.00)
	knn	(2.21, 2.34)	(2.02, 2.14)	(1.43, 1.52)	(2.23, 2.36)	(2.05, 2.17)	(1.45, 1.54)
	wsld	(2.17, 2.30)	(1.99, 2.11)	(1.41, 1.49)	(2.20, 2.33)	(2.02, 2.14)	(1.42, 1.51)
	COSNet	(2.73, 2.87)	(2.50, 2.63)	(1.77, 1.87)	(2.76, 2.91)	(2.53, 2.67)	(1.79, 1.89)
	bagsvm	(2.45, 2.58)	(2.24, 2.37)	(1.58, 1.68)	(2.48, 2.62)	(2.27, 2.40)	(1.60, 1.70)
	rf	(1.52, 1.62)	(1.39, 1.49)	(0.98, 1.05)	(1.54, 1.64)	(1.41, 1.51)	(0.99, 1.07)
	svm	(1.53, 1.64)	(1.41, 1.50)	(0.99, 1.06)	(1.55, 1.66)	(1.42, 1.52)	(1.01, 1.08)

Reference streams

In order to evaluate the extent to which using networks for predicting disease genes is of use compared against not using networks at all, we have also checked the extent to which the gene scores from other data streams in Open Targets could be used to recover known drug targets. To that end, we have computed the metrics between all the remaining streams and the drug targets stream, reusing the partitions from the cross validation folds (see main text).

These metrics are therefore not directly comparable to those presented above for the network-based approaches, as in this case, the concept of cross-validation does not apply. It is rather a data subsetting strategy to compute the estimates, to which the additive models also apply (see table S).

The genes scores from the Open Targets *literature* data stream result in the best alignment with the scores from known drug targets.

There may be some circularity explaining this as the *literature* data stream uses publications mentioning known drug targets and their relation to diseases, and also as a gene with a lot of *literature* describing its relationship to disease may be more likely to be picked as a potential drug target. The *genetic association* data stream is second best in terms of correlation with the known drug target scores, thereby justifying its usage for finding potential targets a posteriori.

Table S: Predictions of the models SA1, SA2, SA3, SA4, SA5 and SA6 (95% confidence intervals after averaging over disease).

Input: streams		STRING			OmniPath		
metric	method (stream)	classic	block	representative	classic	block	representative
auroc	affected_pathway	(0.494, 0.496)	(0.496, 0.498)	(0.505, 0.508)	(0.495, 0.498)	(0.497, 0.500)	(0.506, 0.509)
	animal_model	(0.503, 0.506)	(0.505, 0.508)	(0.514, 0.517)	(0.505, 0.507)	(0.507, 0.509)	(0.516, 0.518)
	genetic_association	(0.516, 0.519)	(0.518, 0.521)	(0.527, 0.530)	(0.517, 0.520)	(0.520, 0.522)	(0.529, 0.531)
	literature	(0.692, 0.694)	(0.693, 0.696)	(0.701, 0.703)	(0.693, 0.695)	(0.695, 0.697)	(0.702, 0.705)
	rna_expression	(0.511, 0.513)	(0.513, 0.515)	(0.522, 0.524)	(0.512, 0.515)	(0.514, 0.517)	(0.523, 0.526)
	somatic_mutation	(0.493, 0.496)	(0.496, 0.498)	(0.505, 0.507)	(0.495, 0.498)	(0.497, 0.500)	(0.506, 0.509)
partial_auroc_0.10	affected_pathway	(0.046, 0.047)	(0.047, 0.049)	(0.054, 0.055)	(0.045, 0.047)	(0.047, 0.048)	(0.053, 0.055)
	animal_model	(0.062, 0.063)	(0.064, 0.066)	(0.072, 0.074)	(0.061, 0.063)	(0.063, 0.065)	(0.072, 0.074)
	genetic_association	(0.081, 0.083)	(0.084, 0.086)	(0.095, 0.097)	(0.080, 0.082)	(0.083, 0.085)	(0.094, 0.096)
	literature	(0.275, 0.279)	(0.282, 0.286)	(0.310, 0.315)	(0.273, 0.277)	(0.280, 0.284)	(0.308, 0.313)
	rna_expression	(0.066, 0.067)	(0.068, 0.070)	(0.077, 0.079)	(0.065, 0.067)	(0.067, 0.069)	(0.076, 0.078)
	somatic_mutation	(0.045, 0.047)	(0.047, 0.048)	(0.053, 0.055)	(0.045, 0.046)	(0.046, 0.048)	(0.053, 0.054)
partial_auroc_0.05	affected_pathway	(0.023, 0.023)	(0.023, 0.024)	(0.027, 0.028)	(0.022, 0.023)	(0.023, 0.024)	(0.027, 0.028)
	animal_model	(0.038, 0.039)	(0.040, 0.041)	(0.046, 0.048)	(0.037, 0.038)	(0.039, 0.040)	(0.045, 0.046)
	genetic_association	(0.054, 0.056)	(0.056, 0.058)	(0.065, 0.067)	(0.053, 0.054)	(0.055, 0.057)	(0.064, 0.066)
	literature	(0.183, 0.186)	(0.189, 0.193)	(0.214, 0.218)	(0.179, 0.182)	(0.185, 0.189)	(0.210, 0.214)
	rna_expression	(0.037, 0.038)	(0.039, 0.040)	(0.045, 0.046)	(0.036, 0.037)	(0.038, 0.039)	(0.044, 0.045)
	somatic_mutation	(0.022, 0.023)	(0.023, 0.024)	(0.027, 0.028)	(0.022, 0.022)	(0.022, 0.023)	(0.026, 0.027)
auprc	affected_pathway	(0.010, 0.010)	(0.010, 0.011)	(0.009, 0.009)	(0.012, 0.012)	(0.012, 0.012)	(0.010, 0.010)
	animal_model	(0.018, 0.018)	(0.018, 0.019)	(0.015, 0.016)	(0.021, 0.021)	(0.021, 0.022)	(0.018, 0.018)
	genetic_association	(0.019, 0.020)	(0.020, 0.020)	(0.016, 0.017)	(0.022, 0.023)	(0.023, 0.024)	(0.019, 0.020)
	literature	(0.050, 0.052)	(0.052, 0.053)	(0.044, 0.045)	(0.059, 0.060)	(0.060, 0.062)	(0.051, 0.052)
	rna_expression	(0.013, 0.013)	(0.013, 0.014)	(0.011, 0.011)	(0.015, 0.016)	(0.016, 0.016)	(0.013, 0.013)
	somatic_mutation	(0.010, 0.010)	(0.010, 0.011)	(0.008, 0.009)	(0.011, 0.012)	(0.012, 0.012)	(0.010, 0.010)
top_20_hits	affected_pathway	(0.21, 0.23)	(0.21, 0.23)	(0.15, 0.17)	(0.20, 0.22)	(0.20, 0.22)	(0.14, 0.16)
	animal_model	(0.75, 0.79)	(0.74, 0.79)	(0.54, 0.58)	(0.70, 0.74)	(0.69, 0.73)	(0.50, 0.54)
	genetic_association	(1.06, 1.12)	(1.05, 1.11)	(0.77, 0.81)	(0.99, 1.05)	(0.98, 1.04)	(0.71, 0.76)
	literature	(1.94, 2.03)	(1.92, 2.01)	(1.40, 1.47)	(1.81, 1.89)	(1.79, 1.88)	(1.30, 1.37)
	rna_expression	(0.44, 0.47)	(0.43, 0.47)	(0.32, 0.34)	(0.41, 0.44)	(0.40, 0.44)	(0.29, 0.32)
	somatic_mutation	(0.20, 0.22)	(0.20, 0.22)	(0.15, 0.16)	(0.19, 0.21)	(0.19, 0.21)	(0.14, 0.15)
top_100_hits	affected_pathway	(1.46, 1.52)	(1.45, 1.51)	(1.09, 1.13)	(1.84, 1.91)	(1.83, 1.90)	(1.37, 1.43)
	animal_model	(1.90, 1.96)	(1.89, 1.95)	(1.41, 1.46)	(2.39, 2.47)	(2.38, 2.46)	(1.78, 1.85)
	genetic_association	(2.15, 2.22)	(2.14, 2.21)	(1.60, 1.65)	(2.71, 2.80)	(2.70, 2.78)	(2.02, 2.09)
	literature	(6.85, 7.01)	(6.82, 6.98)	(5.10, 5.23)	(8.64, 8.84)	(8.61, 8.81)	(6.44, 6.60)
	rna_expression	(1.63, 1.69)	(1.62, 1.68)	(1.21, 1.26)	(2.06, 2.13)	(2.05, 2.12)	(1.53, 1.59)
	somatic_mutation	(1.45, 1.51)	(1.45, 1.50)	(1.08, 1.12)	(1.83, 1.90)	(1.82, 1.89)	(1.36, 1.42)

Interaction effects between CV scheme, network, diseases and methods

As mentioned in the main text, an illustrated by the above tables, in addition to a main effects model, we also considered how model performance could vary with other parameters such as choice of network and disease. In particular, we formally asked whether the differences – or interactions – we observed were in line or greater than those that would be expected by chance.

Interaction effects were omitted from our main analysis to avoid overfitting the data and a corresponding underestimation of the residual error and inflation in statistical significance. Given the large number of combinations possible, this was a risk even where the majority of interactions were not significant. This scenario was however in contrast to the current exploration were the sizes of any such effects were of interest per se. On the other hand, this exploratory analysis shows that the interaction terms that would improve the model involve poorly performing methods. Given their lack of interest in the final recommendations, including such terms would make the formal comparisons cumbersome, without providing any added value.

To simplify our analysis, motivated by standard statistical theory for multi-factorial statistical screening designs [Mon17], high order interactions, such as those between say CV scheme, network and disease, were omitted from our calculations and assumed to be little different to statistical noise. In contrast to standard screening methods, however, which typically address all binary or two level factors, all factor levels where considered adding to the complexity of the plots. In a further deviation, two-way interactions, say, were considered independently from all lower order terms, here one way or main effects, which were removed from the signal prior to analysis. This, done at the cost of over counting by one the total degrees of freedom in our data, served to improve interpretability since otherwise we would have one fewer interaction terms than combinations in the looked for effects. See figure S for an example and the html viewer in the supplementary file `interaction_html_viewer.zip` for other models and interaction terms.

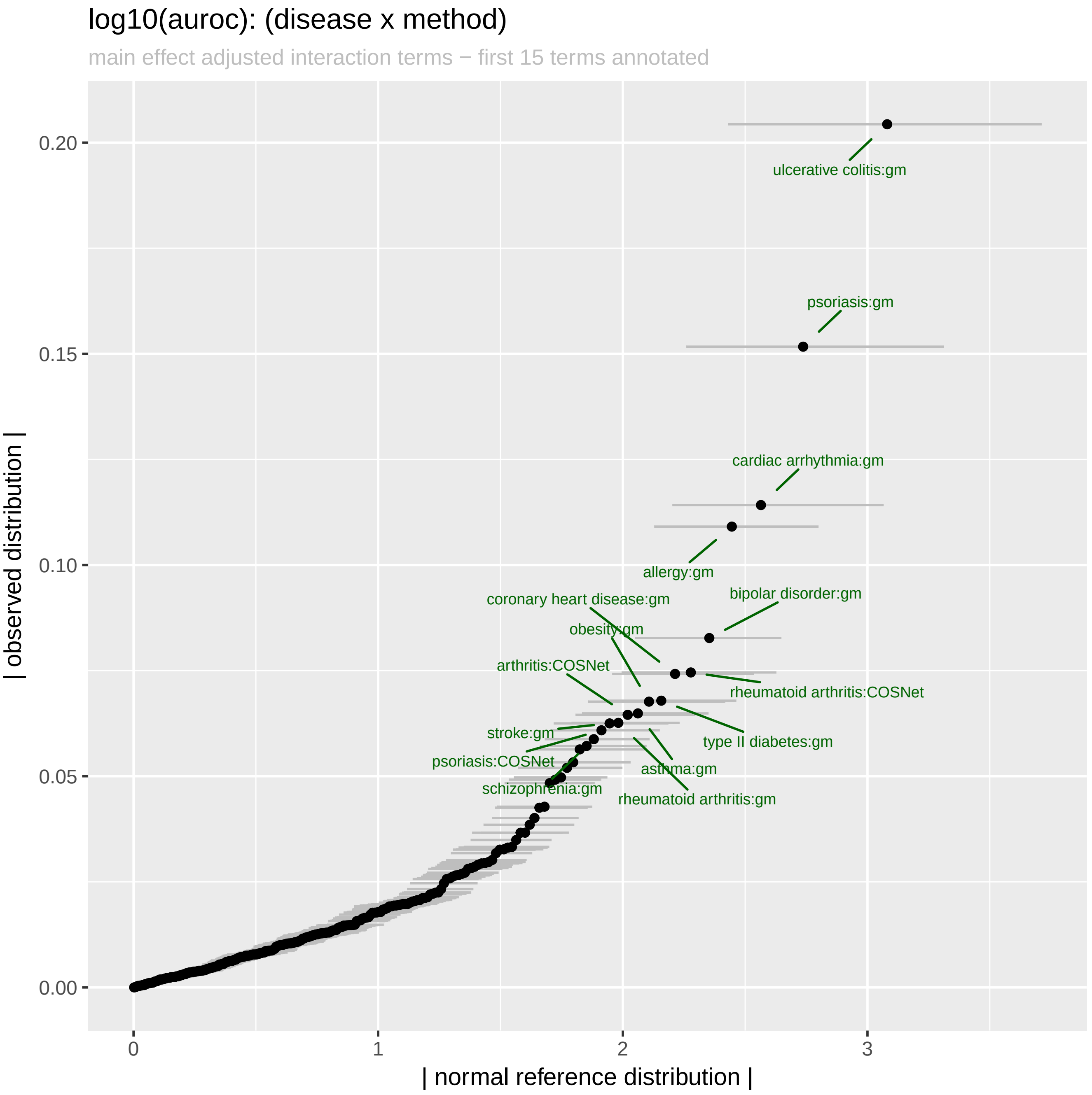

Figure S: **Graphical representation of the disease $\times$ method interaction.** To investigate the possible effects of disease on individual method performance under the log10(AUROC), the absolute values of the the observed deviations from those predicted by a simple main effects model combination are plotted against those values that would be expected by chance alone under an assumption on normally distributed random noise. Deviations upwards from a straight line trend suggest interactions that are larger than would be expected by chance. Due to the use of absolute values -signs of interactions are difficult to interpret and can confuse comparisons- to 'fold over' the two distributions this is typically referred to as a *half normal plot*. To maintain a one to one correspondence between observed deviations and the set of two-way combinations of disease and method which would otherwise be lost by accounting degrees of freedom, main effect contributions for disease and method were removed prior to this analysis. As a guide to the underlying variability, the plot also includes 95% confidence intervals for the distribution of each absolute value normal reference value under re-sampling.

Package versions

Table T: summary of the package versions used in this work and their source of download

Number	Package	Version	Source
1	acepack	1.4.1	CRAN
2	affy	1.54.0	Bioconductor
3	affyio	1.46.0	Bioconductor
4	affyPLM	1.52.1	Bioconductor
5	annotate	1.54.0	Bioconductor
6	AnnotationDbi	1.38.2	Bioconductor
7	arrayQualityMetrics	3.32.0	Bioconductor
8	assertthat	0.2.0	CRAN
9	backports	1.0.5	CRAN
10	base64	2.0	CRAN
11	base64enc	0.1-3	CRAN
12	BBmisc	1.11	CRAN
13	beadarray	2.26.1	Bioconductor
14	BeadDataPackR	1.28.0	Bioconductor
15	BH	1.65.0-1	CRAN
16	Biobase	2.36.2	Bioconductor
17	BiocGenerics	0.22.1	Bioconductor
18	BiocInstaller	1.26.1	Bioconductor
19	biomaRt	2.32.1	Bioconductor
20	Biostrings	2.44.2	Bioconductor
21	bitops	1.0-6	CRAN
22	broom	0.4.3	CRAN
23	Cairo	1.5-9	CRAN
24	caret	6.0-78	CRAN
25	caTools	1.17.1	CRAN
26	checkmate	1.8.2	CRAN
27	chron	2.3-50	CRAN
28	coda	0.19-1	CRAN
29	colorspace	1.3-2	CRAN
30	corrplot	0.84	CRAN
31	COSNet	1.10.0	Bioconductor
32	crayon	1.3.4	CRAN
33	curl	2.8.1	CRAN
34	CVST	0.2-1	CRAN
35	data.table	1.10.4	CRAN
36	DBI	0.6-1	CRAN
37	ddalpha	1.3.1	CRAN
38	DEoptimR	1.0-8	CRAN
39	devtools	1.13.4	CRAN
40	dichromat	2.0-0	CRAN
41	diffuStats	0.101.1	github
42	digest	0.6.12	CRAN
43	dimRed	0.1.0	CRAN
44	doMC	1.3.5	CRAN
45	dplyr	0.5.0	CRAN
46	DRR	0.0.2	CRAN
47	e1071	1.6-8	CRAN
48	EGAD	1.4.1	Bioconductor
49	emmeans	1.1.2	CRAN
50	estimability	1.3	CRAN

51	evaluate	0.10	CRAN
52	expm	0.999-2	CRAN
53	foreach	1.4.3	CRAN
54	formatR	1.5	CRAN
55	Formula	1.2-1	CRAN
56	gcrma	2.48.0	Bioconductor
57	gdata	2.17.0	CRAN
58	gdtools	0.1.7	CRAN
59	genefilter	1.58.1	Bioconductor
60	GenomeInfoDb	1.12.3	Bioconductor
61	GenomeInfoDbData	0.99.0	Bioconductor
62	GenomicRanges	1.28.6	Bioconductor
63	GEOquery	2.42.0	Bioconductor
64	GGally	1.4.0	CRAN
65	ggdendro	0.1-20	CRAN
66	ggplot2	2.2.1	CRAN
67	ggsci	2.8	CRAN
68	git2r	0.20.0	CRAN
69	glue	1.2.0	CRAN
70	gower	0.1.2	CRAN
71	gplots	3.0.1	CRAN
72	graph	1.54.0	Bioconductor
73	gridExtra	2.2.1	CRAN
74	gridSVG	1.6-0	CRAN
75	gsubfn	0.6-6	CRAN
76	gtable	0.2.0	CRAN
77	gtools	3.5.0	CRAN
78	hash	2.2.6	CRAN
79	hexbin	1.27.1	CRAN
80	highr	0.6	CRAN
81	Hmisc	4.0-3	CRAN
82	hms	0.4.1	CRAN
83	htmlTable	1.9	CRAN
84	htmltools	0.3.6	CRAN
85	htmlwidgets	0.9	CRAN
86	httr	1.3.1	CRAN
87	hwriter	1.3.2	CRAN
88	igraph	1.1.2	CRAN
89	illuminaio	0.18.0	Bioconductor
90	impute	1.50.1	Bioconductor
91	ipred	0.9-6	CRAN
92	IRanges	2.10.5	Bioconductor
93	irlba	2.2.1	CRAN
94	irr	0.84	CRAN
95	iterators	1.0.8	CRAN
96	jsonlite	1.5	CRAN
97	kableExtra	0.7.0	CRAN
98	kernlab	0.9-25	CRAN
99	knitr	1.16	CRAN
100	labeling	0.3	CRAN
101	latticeExtra	0.6-28	CRAN
102	lava	1.5.1	CRAN
103	lazyeval	0.2.0	CRAN
104	limma	3.32.10	Bioconductor
105	lpSolve	5.6.13	CRAN
106	lsmeans	2.27-61	CRAN

107	lubridate	1.7.1	CRAN
108	magrittr	1.5	CRAN
109	markdown	0.8	CRAN
110	memoise	1.1.0	CRAN
111	Metrics	0.1.3	CRAN
112	mime	0.5	CRAN
113	MLmetrics	1.1.1	CRAN
114	mlr	2.11	CRAN
115	mnormt	1.5-5	CRAN
116	ModelMetrics	1.1.0	CRAN
117	multcomp	1.4-8	CRAN
118	munsell	0.4.3	CRAN
119	mvtnorm	1.0-6	CRAN
120	NetPreProc	1.1	CRAN
121	numDeriv	2016.8-1	CRAN
122	openssl	0.9.7	CRAN
123	packrat	0.4.8-1	CRAN
124	parallelMap	1.3	CRAN
125	ParamHelpers	1.10	CRAN
126	PerfMeas	1.2.1	CRAN
127	pkgconfig	2.0.1	CRAN
128	plogr	0.1-1	CRAN
129	plotrix	3.7	CRAN
130	plyr	1.8.4	CRAN
131	png	0.1-7	CRAN
132	precrec	0.9.1	CRAN
133	preprocessCore	1.38.1	Bioconductor
134	prettyunits	1.0.2	CRAN
135	pROC	1.10.0	CRAN
136	prodlim	1.6.1	CRAN
137	progress	1.2.0	CRAN
138	proto	1.0.0	CRAN
139	PRROC	1.3	CRAN
140	psych	1.7.8	CRAN
141	purrr	0.2.4	CRAN
142	R6	2.2.2	CRAN
143	randomForest	4.6-12	CRAN
144	RANKS	1.0	CRAN
145	RBGL	1.52.0	Bioconductor
146	RColorBrewer	1.1-2	CRAN
147	Rcpp	0.12.14	CRAN
148	RcppArmadillo	0.8.300.1.0	CRAN
149	RcppParallel	4.3.20	CRAN
150	RcppRoll	0.2.2	CRAN
151	RCurl	1.95-4.8	CRAN
152	readr	1.1.1	CRAN
153	recipes	0.1.1	CRAN
154	reshape	0.8.7	CRAN
155	reshape2	1.4.2	CRAN
156	rlang	0.1.1	CRAN
157	rmarkdown	1.8	CRAN
158	robustbase	0.92-7	CRAN
159	ROCR	1.0-7	CRAN
160	rprojroot	1.3-1	CRAN
161	RSQLite	1.1-2	CRAN
162	rstudioapi	0.7	CRAN

163	rvest	0.3.2	CRAN
164	S4Vectors	0.14.7	Bioconductor
165	sandwich	2.3-4	CRAN
166	scales	0.4.1	CRAN
167	selectr	0.3-1	CRAN
168	setRNG	2013.9-1	CRAN
169	sfsmisc	1.1-1	CRAN
170	sqldf	0.4-11	CRAN
171	stargazer	5.2.1	CRAN
172	STRINGdb	1.16.0	Bioconductor
173	stringi	1.1.5	CRAN
174	stringr	1.2.0	CRAN
175	SVGAnnotation	0.93-2	github
176	svglite	1.2.1	CRAN
177	TH.data	1.0-8	CRAN
178	tibble	1.3.1	CRAN
179	tidyr	0.6.3	CRAN
180	tidyselect	0.2.3	CRAN
181	timeDate	3012.100	CRAN
182	TopKLists	1.0.6	CRAN
183	viridis	0.4.0	CRAN
184	viridisLite	0.2.0	CRAN
185	vsn	3.44.0	Bioconductor
186	whisker	0.3-2	CRAN
187	withr	2.1.1	CRAN
188	XML	3.98-1.12	CRAN
189	xml2	1.2.0	CRAN
190	xtable	1.8-2	CRAN
191	XVector	0.16.0	Bioconductor
192	yaml	2.1.14	CRAN
193	zlibbioc	1.22.0	Bioconductor
194	zoo	1.8-0	CRAN

References

[BMR+16] Matteo Bersanelli, Ettore Mosca, Daniel Remondini, Gastone Castellani, and Luciano Milanesi. Network diffusion-based analysis of high-throughput data for the detection of differentially enriched modules. *Sci. Rep.*, 6(34841):1–12, 2016.

[EBEK11] Sinan Erten, Gurkan Bebek, Rob M Ewing, and Mehmet Koyutürk. Dada: degree-aware algorithms for network-based disease gene prioritization. *BioData mining*, 4(1):19, 2011.

[GM07] Ilan Gronau and Shlomo Moran. Optimal implementations of upgma and other common clustering algorithms. *Information Processing Letters*, 104(6):205–210, 2007.

[Mon17] Douglas C Montgomery. *Design and analysis of experiments.* John wiley & sons, 2017.

[The17] TheUniProtConsortium. Uniprot: the universal protein knowledgebase. *Nucleic Acids Res.*, 45(D1):D158–D169, 2017.

[ZAA+18] Daniel R Zerbino, Premanand Achuthan, Wasiu Akanni, MRidwan Amode, Daniel Barrell, Jyothish Bhai, Konstantinos Billis, Carla Cummins, Astrid Gall, Carlos García Girón, Laurent Gil, Leo Gordon, Leanne Haggerty, Erin Haskell, Thibaut Hourlier, Osagie G Izuogu, Sophie H Janacek, Thomas Juettemann, Jimmy Kiang To, Matthew R Laird, Ilias Lavidas, Zhicheng Liu, Jane E Loveland, Thomas Maurel, William McLaren, Benjamin Moore, Jonathan Mudge, Daniel N Murphy, Victoria Newman, Michael Nuhn, Denye Ogeh, Chuang Kee Ong, Anne Parker, Mateus Patricio, Harpreet Singh Riat, Helen Schuilenburg, Dan Sheppard, Helen Sparrow, Kieron Taylor, Anja Thormann, Alessandro Vullo, Brandon Walts, Amonida Zadissa, Adam Frankish, Sarah E Hunt, Myrto Kostadima, Nicholas Langridge, Fergal J Martin, Matthieu Muffato, Emily Perry, Magali Ruffier, Dan M Staines, Stephen J Trevanion, Bronwen L Aken, Fiona Cunningham, Andrew Yates, and Paul Flicek. Ensembl 2018. *Nucleic Acids Res.*, 46(D1):D754–D761, 2018.

[Zuu09] Zuur, Alain F and Ieno, Elena N and Walker, Neil J and Saveliev, Anatoly A and Smith, Graham M. GLM and GAM for count data. In *Mixed effects models and extensions in ecology with R*, pages 209–243. Springer, 2009.

Note: the remaining numeric references, which were not included here, are coincident with those in the main body.
