# Supplementary material for: Benchmarking network propagation methods for disease gene identification
Source: PLoS Comput Biol. 2019 Sep 3;15(9):e1007276. doi: 10.1371/journal.pcbi.1007276 (PMC6743778; doi:10.1371/journal.pcbi.1007276)
Supplement: S1 File — Complementary single-disease MDS plots and distance matrices. (ZIP) [file pcbi.1007276.s002.zip › Single Disease x input type MDS plot Data.pdf]

Spearman's footrule distance matrices input for examples of individual multidimensional scaling (MDS) plots

drugs_OnlySeeds_SpearmanFootRule_vs_cardiac_arrhythmia_STRING

	raw	gm	mc	z	ppr	EGAD	wsld	knn	bagsvm	svm	rf
raw	0	0	10120	5358	5852	5284	6332	6520	28	6232	5818
gm	0	0	10120	5358	5852	5284	6332	6520	28	6232	5818
mc	10120	10120	0	8850	8646	9090	10488	10452	10122	9392	8872
z	5358	5358	8850	0	2054	3148	10466	10570	5344	6414	4704
ppr	5852	5852	8646	2054	0	3572	10782	10910	5844	6872	5216
EGAD	5284	5284	9090	3148	3572	0	10428	10530	5280	6432	4504
wsld	6332	6332	10488	10466	10782	10428	0	298	6344	7868	9000
knn	6520	6520	10452	10570	10910	10530	298	0	6532	7942	9108
bagsvm	28	28	10122	5344	5844	5280	6344	6532	0	6230	5818
svm	6232	6232	9392	6414	6872	6432	7868	7942	6230	0	4800
rf	5818	5818	8872	4704	5216	4504	9000	9108	5818	4800	0

drugs_OnlySeeds_SpearmanFootRule_vs_cardiac_arrhythmia_OMNIPATH

	raw	gm	mc	z	ppr	EGAD	wsld	knn	bagsvm	svm	rf
raw	0	20	6246	3524	4540	4358	1814	1668	32	4252	3382
gm	20	0	6246	3524	4540	4358	1814	1668	38	4252	3378
mc	6246	6246	0	4458	5188	5598	6426	6328	6258	4932	4762
z	3524	3524	4458	0	2850	3782	4930	4792	3522	2432	2900
ppr	4540	4540	5188	2850	0	3886	5992	5776	4544	2620	3362
EGAD	4358	4358	5598	3782	3886	0	5262	5132	4358	3846	3792
wsld	1814	1814	6426	4930	5992	5262	0	318	1812	5602	4666
knn	1668	1668	6328	4792	5776	5132	318	0	1666	5494	4548
bagsvm	32	38	6258	3522	4544	4358	1812	1666	0	4258	3386
svm	4252	4252	4932	2432	2620	3846	5602	5494	4258	0	2494
rf	3382	3378	4762	2900	3362	3792	4666	4548	3386	2494	0

genetic_OnlySeeds_SpearmanFootRule_vs_cardiac_arrhythmia_STRING

	raw	gm	mc	z	ppr	EGAD	wsld	knn	bagsvm	svm	rf
raw	0	0	1834	1116	2340	2060	88	36	8	2548	2338
gm	0	0	1834	1116	2340	2060	88	36	8	2548	2338
mc	1834	1834	0	1716	1510	1510	1858	1840	1832	1656	1604
z	1116	1116	1716	0	2246	1618	1158	1132	1120	2320	1820
ppr	2340	2340	1510	2246	0	1338	2376	2348	2336	1022	1176
EGAD	2060	2060	1510	1618	1338	0	2110	2074	2060	1250	1384
wsld	88	88	1858	1158	2376	2110	0	64	88	2570	2382
knn	36	36	1840	1132	2348	2074	64	0	34	2560	2352
bagsvm	8	8	1832	1120	2336	2060	88	34	0	2552	2344
svm	2548	2548	1656	2320	1022	1250	2570	2560	2552	0	894
rf	2338	2338	1604	1820	1176	1384	2382	2352	2344	894	0

genetic_OnlySeeds_SpearmanFootRule_vs_cardiac_arrhythmia_OMNIPATH

	raw	gm	mc	z	ppr	EGAD	wsld	knn	bagsvm	svm	rf
raw	0	0	1386	732	1390	1566	22	6	0	1682	1564
gm	0	0	1386	732	1390	1566	22	6	0	1682	1564
mc	1386	1386	0	1152	984	746	1390	1390	1386	1004	1006
z	732	732	1152	0	1550	1262	740	732	732	1450	1318
ppr	1390	1390	984	1550	0	862	1390	1390	1390	776	1022
EGAD	1566	1566	746	1262	862	0	1572	1566	1566	656	738
wsld	22	22	1390	740	1390	1572	0	16	22	1682	1568
knn	6	6	1390	732	1390	1566	16	0	6	1682	1568
bagsvm	0	0	1386	732	1390	1566	22	6	0	1682	1564
svm	1682	1682	1004	1450	776	656	1682	1682	1682	0	566
rf	1564	1564	1006	1318	1022	738	1568	1568	1564	566	0

drugs_WithoutSeeds_SpearmanFootRule_vs_cardiac_arrhythmia_STRING

	raw	gm	mc	z	ppr	EGAD	wsld	knn	bagsvm	svm	rf	COSNet
raw	0	0	6095	4016	5075	5626	6158	6593	387	7201	7843	9140
gm	0	0	6095	4016	5075	5626	6158	6593	387	7201	7843	9140
mc	6095	6095	0	4209	5867	8081	7895	8321	6103	9214	8754	9911
z	4016	4016	4209	0	4863	6937	7267	7810	4141	8452	8152	9673
ppr	5075	5075	5867	4863	0	7306	6952	7666	5108	8134	8135	9658
EGAD	5626	5626	8081	6937	7306	0	5896	5944	5471	7411	7094	8507
wsld	6158	6158	7895	7267	6952	5896	0	1562	6136	6446	7117	8973
knn	6593	6593	8321	7810	7666	5944	1562	0	6581	6325	6978	8990
bagsvm	387	387	6103	4141	5108	5471	6136	6581	0	7184	7821	9096
svm	7201	7201	9214	8452	8134	7411	6446	6325	7184	0	6098	9041
rf	7843	7843	8754	8152	8135	7094	7117	6978	7821	6098	0	9316
COSNet	9140	9140	9911	9673	9658	8507	8973	8990	9096	9041	9316	0

drugs_WithoutSeeds_SpearmanFootRule_vs_cardiac_arrhythmia_OMNIPATH

	raw	gm	mc	z	ppr	EGAD	wsld	knn	bagsvm	svm	rf	COSNet
raw	0	0	6390	5423	7766	5494	2496	2026	1030	5146	6517	7786
gm	0	0	6390	5419	7766	5492	2496	2026	1034	5142	6517	7786
mc	6390	6390	0	6267	7604	7154	6934	6657	6467	6811	6792	8776
z	5423	5419	6267	0	6358	7505	6104	6046	5630	6433	6972	9089
ppr	7766	7766	7604	6358	0	9033	8427	8130	7972	8496	8319	9718
EGAD	5494	5492	7154	7505	9033	0	5293	5285	5458	6922	7705	6027
wsld	2496	2496	6934	6104	8427	5293	0	1076	2657	6002	7160	7527
knn	2026	2026	6657	6046	8130	5285	1076	0	2212	5706	6738	7626
bagsvm	1030	1034	6467	5630	7972	5458	2657	2212	0	5269	6558	7845
svm	5146	5142	6811	6433	8496	6922	6002	5706	5269	0	6408	8608
rf	6517	6517	6792	6972	8319	7705	7160	6738	6558	6408	0	8966
COSNet	7786	7786	8776	9089	9718	6027	7527	7626	7845	8608	8966	0

genetic_WithoutSeeds_SpearmanFootRule_vs_cardiac_arrhythmia_STRING

	raw	gm	mc	z	ppr	EGAD	wsld	knn	bagsvm	svm	rf	COSNet
raw	0	0	6414	5718	7532	7292	3120	798	846	9709	9613	9601
gm	0	0	6414	5718	7532	7292	3120	798	846	9709	9613	9601
mc	6414	6414	0	5831	7956	8298	6964	6527	6457	9370	9568	9584
z	5718	5718	5831	0	6133	9223	7067	5989	5781	8963	9737	10000
ppr	7532	7532	7956	6133	0	9432	8073	7523	7651	7008	9703	10000
EGAD	7292	7292	8298	9223	9432	0	7026	7196	7391	9683	9629	8946
wsld	3120	3120	6964	7067	8073	7026	0	2643	3190	9809	9656	9478
knn	798	798	6527	5989	7523	7196	2643	0	1107	9742	9638	9533
bagsvm	846	846	6457	5781	7651	7391	3190	1107	0	9702	9607	9580
svm	9709	9709	9370	8963	7008	9683	9809	9742	9702	0	9856	10000
rf	9613	9613	9568	9737	9703	9629	9656	9638	9607	9856	0	10000
COSNet	9601	9601	9584	10000	10000	8946	9478	9533	9580	10000	10000	0

genetic_WithoutSeeds_SpearmanFootRule_vs_cardiac_arrhythmia_OMNIPATH

	raw	gm	mc	z	ppr	EGAD	wsld	knn	bagsvm	svm	rf	COSNet
raw	0	0	7609	6382	8711	6266	999	564	1212	10000	7925	9066
gm	0	0	7609	6382	8711	6266	999	566	1212	10000	7925	9066
mc	7609	7609	0	8354	9162	7400	7622	7736	7650	9716	9103	9001
z	6382	6382	8354	0	6790	8263	6486	6467	6384	8631	8006	9875
ppr	8711	8711	9162	6790	0	9471	8805	8803	8611	4723	8896	10000
EGAD	6266	6266	7400	8263	9471	0	6192	6245	6231	10000	8993	8089
wsld	999	999	7622	6486	8805	6192	0	674	1248	10000	7873	9057
knn	564	566	7736	6467	8803	6245	674	0	1225	10000	7890	9061
bagsvm	1212	1212	7650	6384	8611	6231	1248	1225	0	10000	7837	9120
svm	10000	10000	9716	8631	4723	10000	10000	10000	10000	0	9582	10000
rf	7925	7925	9103	8006	8896	8993	7873	7890	7837	9582	0	9769
COSNet	9066	9066	9001	9875	10000	8089	9057	9061	9120	10000	9769	0

drugs_OnlySeeds_SpearmanFootRule_vs_multiple_sclerosis_STRING

	raw	gm	mc	z	ppr	EGAD	wsld	knn	bagsvm	svm	rf
raw	0	0	9414	7232	7666	6274	3236	3290	50	7922	7156
gm	0	0	9414	7232	7666	6274	3236	3290	50	7922	7156
mc	9414	9414	0	8078	8216	8106	9722	9724	9410	8684	8054
z	7232	7232	8078	0	2544	2860	9642	9672	7218	4722	3298
ppr	7666	7666	8216	2544	0	3446	9852	9878	7664	3626	3980
EGAD	6274	6274	8106	2860	3446	0	8712	8748	6272	4762	3510
wsld	3236	3236	9722	9642	9852	8712	0	152	3272	9170	9154
knn	3290	3290	9724	9672	9878	8748	152	0	3326	9180	9194
bagsvm	50	50	9410	7218	7664	6272	3272	3326	0	7922	7152
svm	7922	7922	8684	4722	3626	4762	9170	9180	7922	0	4434
rf	7156	7156	8054	3298	3980	3510	9154	9194	7152	4434	0

drugs_OnlySeeds_SpearmanFootRule_vs_multiple_sclerosis_OMNIPATH

	raw	gm	mc	z	ppr	EGAD	wsld	knn	bagsvm	svm	rf
raw	0	4	7304	5990	7840	7576	3722	3668	58	5750	6742
gm	4	0	7304	5990	7840	7576	3722	3668	62	5750	6742
mc	7304	7304	0	4522	5566	5900	8858	8814	7306	6196	5460
z	5990	5990	4522	0	3860	4422	8306	8236	6010	4548	3680
ppr	7840	7840	5566	3860	0	4404	9882	9804	7854	4228	4102
EGAD	7576	7576	5900	4422	4404	0	9026	9000	7586	4710	4190
wsld	3722	3722	8858	8306	9882	9026	0	264	3698	7528	8226
knn	3668	3668	8814	8236	9804	9000	264	0	3654	7482	8164
bagsvm	58	62	7306	6010	7854	7586	3698	3654	0	5756	6752
svm	5750	5750	6196	4548	4228	4710	7528	7482	5756	0	3026
rf	6742	6742	5460	3680	4102	4190	8226	8164	6752	3026	0

genetic_OnlySeeds_SpearmanFootRule_vs_multiple_sclerosis_STRING

	raw	gm	mc	z	ppr	EGAD	wsld	knn	bagsvm	svm	rf
raw	0	0	1990	1112	2036	2158	54	28	8	1910	1834
gm	0	0	1990	1112	2036	2158	54	28	8	1910	1834
mc	1990	1990	0	1598	1242	1302	2018	2000	1990	1570	1484
z	1112	1112	1598	0	1846	1572	1150	1130	1114	1766	1342
ppr	2036	2036	1242	1846	0	918	2054	2040	2036	1490	1526
EGAD	2158	2158	1302	1572	918	0	2186	2164	2160	1444	1442
wsld	54	54	2018	1150	2054	2186	0	28	52	1922	1840
knn	28	28	2000	1130	2040	2164	28	0	30	1920	1836
bagsvm	8	8	1990	1114	2036	2160	52	30	0	1908	1836
svm	1910	1910	1570	1766	1490	1444	1922	1920	1908	0	1356
rf	1834	1834	1484	1342	1526	1442	1840	1836	1836	1356	0

genetic_OnlySeeds_SpearmanFootRule_vs_multiple_sclerosis_OMNIPATH

	raw	gm	mc	z	ppr	EGAD	wsld	knn	bagsvm	svm	rf
raw	0	0	1408	882	1450	1694	16	10	4	828	1330
gm	0	0	1408	882	1450	1694	16	10	4	828	1330
mc	1408	1408	0	1114	1066	900	1410	1412	1408	1350	1076
z	882	882	1114	0	1574	1200	886	884	882	1150	1004
ppr	1450	1450	1066	1574	0	960	1454	1450	1450	1446	1308
EGAD	1694	1694	900	1200	960	0	1696	1690	1694	1580	1016
wsld	16	16	1410	886	1454	1696	0	10	12	826	1336
knn	10	10	1412	884	1450	1690	10	0	6	826	1330
bagsvm	4	4	1408	882	1450	1694	12	6	0	828	1330
svm	828	828	1350	1150	1446	1580	826	826	828	0	1330
rf	1330	1330	1076	1004	1308	1016	1336	1330	1330	1330	0

drugs_WithoutSeeds_SpearmanFootRule_vs_multiple_sclerosis_STRING

	raw	gm	mc	z	ppr	EGAD	wsld	knn	bagsvm	svm	rf	COSNet
**raw**	0	0	9317	8912	8846	7830	6075	6329	933	9263	8626	9225
**gm**	0	0	9317	8912	8846	7830	6075	6329	933	9263	8626	9225
**mc**	9317	9317	0	5917	7559	9721	9752	9749	9390	8660	8718	10000
**z**	8912	8912	5917	0	5566	9401	9330	9354	8924	7934	8797	9945
**ppr**	8846	8846	7559	5566	0	9325	9008	9017	8847	6612	8724	9752
**EGAD**	7830	7830	9721	9401	9325	0	7853	8065	7788	9409	9007	7428
**wsld**	6075	6075	9752	9330	9008	7853	0	1433	6030	9305	9036	8819
**knn**	6329	6329	9749	9354	9017	8065	1433	0	6281	9300	9097	8921
**bagsvm**	933	933	9390	8924	8847	7788	6030	6281	0	9259	8626	9185
**svm**	9263	9263	8660	7934	6612	9409	9305	9300	9259	0	8500	10000
**rf**	8626	8626	8718	8797	8724	9007	9036	9097	8626	8500	0	9930
**COSNet**	9225	9225	10000	9945	9752	7428	8819	8921	9185	10000	9930	0

drugs_WithoutSeeds_SpearmanFootRule_vs_multiple_sclerosis_OMNIPATH

	raw	gm	mc	z	ppr	EGAD	wsld	knn	bagsvm	svm	rf	COSNet
**raw**	0	0	7312	6267	8059	7434	4752	4539	1608	6403	6798	8052
**gm**	0	0	7312	6267	8059	7434	4752	4539	1608	6403	6796	8052
**mc**	7312	7312	0	6475	7322	8310	8240	8228	7142	8251	7479	8721
**z**	6267	6267	6475	0	5661	8604	7932	7709	6399	8076	7353	9117
**ppr**	8059	8059	7322	5661	0	9236	9047	8783	8141	8843	8268	9558
**EGAD**	7434	7434	8310	8604	9236	0	6526	6775	7026	8169	9005	3129
**wsld**	4752	4752	8240	7932	9047	6526	0	1173	4307	6660	7552	7075
**knn**	4539	4539	8228	7709	8783	6775	1173	0	4078	6657	7493	7084
**bagsvm**	1608	1608	7142	6399	8141	7026	4307	4078	0	6453	6891	7623
**svm**	6403	6403	8251	8076	8843	8169	6660	6657	6453	0	6100	8456
**rf**	6798	6796	7479	7353	8268	9005	7552	7493	6891	6100	0	9286
**COSNet**	8052	8052	8721	9117	9558	3129	7075	7084	7623	8456	9286	0

genetic_WithoutSeeds_SpearmanFootRule_vs_multiple_sclerosis_STRING

	raw	gm	mc	z	ppr	EGAD	wsld	knn	bagsvm	svm	rf	COSNet
**raw**	0	0	8747	8679	8609	8241	2264	2047	1918	9128	9063	9669
**gm**	0	0	8747	8679	8609	8241	2264	2047	1918	9128	9063	9669
**mc**	8747	8747	0	7769	8884	8968	8760	8762	8708	9582	9370	9709
**z**	8679	8679	7769	0	6061	9773	9047	9025	8843	9668	9548	10000
**ppr**	8609	8609	8884	6061	0	9739	8979	8968	8584	9815	10000	9958
**EGAD**	8241	8241	8968	9773	9739	0	8193	8202	8152	9682	9487	8826
**wsld**	2264	2264	8760	9047	8979	8193	0	348	1685	8952	8920	9594
**knn**	2047	2047	8762	9025	8968	8202	348	0	1594	8932	8888	9598
**bagsvm**	1918	1918	8708	8843	8584	8152	1685	1594	0	9115	8701	9604
**svm**	9128	9128	9582	9668	9815	9682	8952	8932	9115	0	4500	10000
**rf**	9063	9063	9370	9548	10000	9487	8920	8888	8701	4500	0	10000
**COSNet**	9669	9669	9709	10000	9958	8826	9594	9598	9604	10000	10000	0

genetic_WithoutSeeds_SpearmanFootRule_vs_multiple_sclerosis_OMNIPATH

	raw	gm	mc	z	ppr	EGAD	wsld	knn	bagsvm	svm	rf	COSNet
**raw**	0	0	7188	6665	8304	8596	1197	1104	1266	10000	9475	9522
**gm**	0	0	7188	6665	8304	8596	1197	1104	1266	10000	9475	9522
**mc**	7188	7188	0	7385	8057	7537	7361	7356	7054	10000	9773	9325
**z**	6665	6665	7385	0	5560	9348	6966	6875	6684	9944	9514	9901
**ppr**	8304	8304	8057	5560	0	9753	8354	8356	8316	10000	9860	9923
**EGAD**	8596	8596	7537	9348	9753	0	8562	8578	8623	9927	9952	8235
**wsld**	1197	1197	7361	6966	8354	8562	0	352	1609	10000	9499	9511
**knn**	1104	1104	7356	6875	8356	8578	352	0	1566	10000	9481	9512
**bagsvm**	1266	1266	7054	6684	8316	8623	1609	1566	0	10000	9648	9496
**svm**	10000	10000	10000	9944	10000	9927	10000	10000	10000	0	10000	10000
**rf**	9475	9475	9773	9514	9860	9952	9499	9481	9648	10000	0	10000
**COSNet**	9522	9522	9325	9901	9923	8235	9511	9512	9496	10000	10000	0

drugs_OnlySeeds_SpearmanFootRule_vs_Parkinson's_disease_STRING

	raw	gm	mc	z	ppr	EGAD	wsld	knn	bagsvm	svm	rf
**raw**	0	0	6490	4686	4820	3950	3104	3172	24	4562	4166
**gm**	0	0	6490	4686	4820	3950	3104	3172	24	4562	4166
**mc**	6490	6490	0	5494	5710	5952	6606	6644	6490	6106	5646
**z**	4686	4686	5494	0	1930	3032	7132	7204	4690	4002	3014
**ppr**	4820	4820	5710	1930	0	2910	6854	6920	4824	3860	3324
**EGAD**	3950	3950	5952	3032	2910	0	6248	6282	3950	4130	2992
**wsld**	3104	3104	6606	7132	6854	6248	0	130	3092	5368	6060
**knn**	3172	3172	6644	7204	6920	6282	130	0	3160	5400	6094
**bagsvm**	24	24	6490	4690	4824	3950	3092	3160	0	4558	4160
**svm**	4562	4562	6106	4002	3860	4130	5368	5400	4558	0	2716
**rf**	4166	4166	5646	3014	3324	2992	6060	6094	4160	2716	0

drugs_OnlySeeds_SpearmanFootRule_vs_Parkinson's_disease_OMNIPATH

	raw	gm	mc	z	ppr	EGAD	wsld	knn	bagsvm	svm	rf
**raw**	0	8	5284	3742	4774	4778	506	390	36	5250	4450
**gm**	8	0	5284	3742	4774	4778	508	390	36	5252	4448
**mc**	5284	5284	0	3430	4308	4050	5284	5302	5290	4026	3384
**z**	3742	3742	3430	0	3136	3426	4134	4030	3742	3442	2842
**ppr**	4774	4774	4308	3136	0	3568	5164	5010	4774	2204	2878
**EGAD**	4778	4778	4050	3426	3568	0	4786	4714	4780	3574	3546
**wsld**	506	508	5284	4134	5164	4786	0	214	504	5594	4742
**knn**	390	390	5302	4030	5010	4714	214	0	382	5494	4654
**bagsvm**	36	36	5290	3742	4774	4780	504	382	0	5254	4444
**svm**	5250	5252	4026	3442	2204	3574	5594	5494	5254	0	2514
**rf**	4450	4448	3384	2842	2878	3546	4742	4654	4444	2514	0

genetic_OnlySeeds_SpearmanFootRule_vs_Parkinson's_disease_STRING

	raw	gm	mc	z	ppr	EGAD	wsld	knn	bagsvm	svm	rf
**raw**	0	0	872	510	1130	1010	22	10	6	690	1084
**gm**	0	0	872	510	1130	1010	22	10	6	690	1084
**mc**	872	872	0	760	868	666	882	878	870	1118	880
**z**	510	510	760	0	1224	820	524	516	512	838	954
**ppr**	1130	1130	868	1224	0	894	1128	1132	1130	1124	864
**EGAD**	1010	1010	666	820	894	0	1026	1014	1012	952	888
**wsld**	22	22	882	524	1128	1026	0	14	24	700	1086
**knn**	10	10	878	516	1132	1014	14	0	12	694	1084
**bagsvm**	6	6	870	512	1130	1012	24	12	0	692	1080
**svm**	690	690	1118	838	1124	952	700	694	692	0	1160
**rf**	1084	1084	880	954	864	888	1086	1084	1080	1160	0

genetic_OnlySeeds_SpearmanFootRule_vs_Parkinson's_disease_OMNIPATH

	raw	gm	mc	z	ppr	EGAD	wsld	knn	bagsvm	svm	rf
**raw**	0	0	666	344	682	662	6	4	2	670	680
**gm**	0	0	666	344	682	662	6	4	2	670	680
**mc**	666	666	0	522	436	328	666	666	666	478	422
**z**	344	344	522	0	706	528	344	344	344	666	626
**ppr**	682	682	436	706	0	414	682	682	682	288	370
**EGAD**	662	662	328	528	414	0	662	662	662	376	444
**wsld**	6	6	666	344	682	662	0	2	8	670	680
**knn**	4	4	666	344	682	662	2	0	6	670	680
**bagsvm**	2	2	666	344	682	662	8	6	0	670	680
**svm**	670	670	478	666	288	376	670	670	670	0	358
**rf**	680	680	422	626	370	444	680	680	680	358	0

drugs_WithoutSeeds_SpearmanFootRule_vs_Parkinson's_disease_STRING

	raw	gm	mc	z	ppr	EGAD	wsld	knn	bagsvm	svm	rf	COSNet
raw	0	0	9656	8740	8091	6717	5516	5685	710	7920	8278	9261
gm	0	0	9656	8740	8091	6717	5516	5685	710	7920	8278	9261
mc	9656	9656	0	7585	8381	9625	9700	9700	9654	9488	9350	10000
z	8740	8740	7585	0	5129	9097	9162	9182	8744	8781	8091	9906
ppr	8091	8091	8381	5129	0	8774	8287	8345	8099	9067	8512	9827
EGAD	6717	6717	9625	9097	8774	0	6312	6273	6700	8341	8516	8264
wsld	5516	5516	9700	9162	8287	6312	0	911	5440	7472	8382	9296
knn	5685	5685	9700	9182	8345	6273	911	0	5614	7535	8400	9305
bagsvm	710	710	9654	8744	8099	6700	5440	5614	0	7927	8278	9261
svm	7920	7920	9488	8781	9067	8341	7472	7535	7927	0	6552	9408
rf	8278	8278	9350	8091	8512	8516	8382	8400	8278	6552	0	9748
COSNet	9261	9261	10000	9906	9827	8264	9296	9305	9261	9408	9748	0

drugs_WithoutSeeds_SpearmanFootRule_vs_Parkinson's_disease_OMNIPATH

	raw	gm	mc	z	ppr	EGAD	wsld	knn	bagsvm	svm	rf	COSNet
raw	0	0	8029	6680	8086	6719	2448	2147	1078	6686	6451	7976
gm	0	0	8029	6678	8086	6719	2448	2147	1080	6686	6451	7974
mc	8029	8029	0	6615	7740	8410	8355	8272	8056	8060	7863	9313
z	6680	6678	6615	0	6006	8701	7382	7242	6694	6639	7297	9347
ppr	8086	8086	7740	6006	0	9487	8395	8331	8100	8107	8744	9727
EGAD	6719	6719	8410	8701	9487	0	6077	6032	6814	8739	8726	5174
wsld	2448	2448	8355	7382	8395	6077	0	836	2411	7666	7392	7737
knn	2147	2147	8272	7242	8331	6032	836	0	2214	7456	7217	7757
bagsvm	1078	1080	8056	6694	8100	6814	2411	2214	0	6971	6489	8047
svm	6686	6686	8060	6639	8107	8739	7666	7456	6971	0	6626	9441
rf	6451	6451	7863	7297	8744	8726	7392	7217	6489	6626	0	9365
COSNet	7976	7974	9313	9347	9727	5174	7737	7757	8047	9441	9365	0

genetic_WithoutSeeds_SpearmanFootRule_vs_Parkinson's_disease_STRING

	raw	gm	mc	z	ppr	EGAD	wsld	knn	bagsvm	svm	rf	COSNet
raw	0	0	6030	6682	8498	6181	1035	514	1112	9850	10000	9452
gm	0	0	6030	6682	8498	6181	1035	514	1112	9850	10000	9452
mc	6030	6030	0	7100	8424	6529	6200	6071	5943	10000	10000	9223
z	6682	6682	7100	0	6376	9077	7027	6865	6961	9905	10000	9937
ppr	8498	8498	8424	6376	0	9533	8744	8608	8481	9929	10000	10000
EGAD	6181	6181	6529	9077	9533	0	6152	6051	6165	10000	10000	8851
wsld	1035	1035	6200	7027	8744	6152	0	704	1332	9857	10000	9426
knn	514	514	6071	6865	8608	6051	704	0	950	9852	10000	9433
bagsvm	1112	1112	5943	6961	8481	6165	1332	950	0	9855	10000	9378
svm	9850	9850	10000	9905	9929	10000	9857	9852	9855	0	10000	10000
rf	10000	10000	10000	10000	10000	10000	10000	10000	10000	10000	0	10000
COSNet	9452	9452	9223	9937	10000	8851	9426	9433	9378	10000	10000	0

genetic_WithoutSeeds_SpearmanFootRule_vs_Parkinson's_disease_OMNIPATH

	raw	gm	mc	z	ppr	EGAD	wsld	knn	bagsvm	svm	rf	COSNet
raw	0	0	5818	5887	8801	5384	665	293	1733	9366	8670	8160
gm	0	0	5818	5887	8801	5384	665	293	1733	9366	8670	8160
mc	5818	5818	0	7397	9228	5274	5721	5810	5615	9297	9180	7759
z	5887	5887	7397	0	6863	7487	6079	5952	5878	9369	8809	9041
ppr	8801	8801	9228	6863	0	9305	8901	8819	8789	9478	9246	9745
EGAD	5384	5384	5274	7487	9305	0	5372	5352	5464	9393	9534	7166
wsld	665	665	5721	6079	8901	5372	0	439	1852	9376	8875	8120
knn	293	293	5810	5952	8819	5352	439	0	1740	9366	8661	8128
bagsvm	1733	1733	5615	5878	8789	5464	1852	1740	0	9305	8568	7931
svm	9366	9366	9297	9369	9478	9393	9376	9366	9305	0	5997	9227
rf	8670	8670	9180	8809	9246	9534	8875	8661	8568	5997	0	9724
COSNet	8160	8160	7759	9041	9745	7166	8120	8128	7931	9227	9724	0

drugs_OnlySeeds_SpearmanFootRule_vs_schizophrenia_STRING

	raw	gm	mc	z	ppr	EGAD	wsld	knn	bagsvm	svm	rf
**raw**	0	0	9044	6258	6346	4954	3778	3948	70	6858	6740
**gm**	0	0	9044	6258	6346	4954	3778	3948	70	6858	6740
**mc**	9044	9044	0	7022	7112	7382	9772	9750	9048	7172	6988
**z**	6258	6258	7022	0	2026	2928	9418	9550	6268	3616	3888
**ppr**	6346	6346	7112	2026	0	2980	9132	9260	6338	3690	4360
**EGAD**	4954	4954	7382	2928	2980	0	7850	7920	4944	3836	3944
**wsld**	3778	3778	9772	9418	9132	7850	0	252	3740	9088	8920
**knn**	3948	3948	9750	9550	9260	7920	252	0	3910	9132	8990
**bagsvm**	70	70	9048	6268	6338	4944	3740	3910	0	6854	6738
**svm**	6858	6858	7172	3616	3690	3836	9088	9132	6854	0	3982
**rf**	6740	6740	6988	3888	4360	3944	8920	8990	6738	3982	0

drugs_OnlySeeds_SpearmanFootRule_vs_schizophrenia_OMNIPATH

	raw	gm	mc	z	ppr	EGAD	wsld	knn	bagsvm	svm	rf
**raw**	0	14	7104	4202	5312	4952	1850	1698	20	5160	4508
**gm**	14	0	7104	4202	5312	4952	1850	1698	34	5160	4508
**mc**	7104	7104	0	4904	6170	6158	7274	7264	7108	5378	5850
**z**	4202	4202	4904	0	3110	3876	5850	5686	4206	2488	2950
**ppr**	5312	5312	6170	3110	0	3708	6864	6680	5310	3030	3698
**EGAD**	4952	4952	6158	3876	3708	0	5976	5854	4950	3892	3840
**wsld**	1850	1850	7274	5850	6864	5976	0	300	1850	6712	5912
**knn**	1698	1698	7264	5686	6680	5854	300	0	1692	6580	5804
**bagsvm**	20	34	7108	4206	5310	4950	1850	1692	0	5160	4504
**svm**	5160	5160	5378	2488	3030	3892	6712	6580	5160	0	2286
**rf**	4508	4508	5850	2950	3698	3840	5912	5804	4504	2286	0

genetic_OnlySeeds_SpearmanFootRule_vs_schizophrenia_STRING

	raw	gm	mc	z	ppr	EGAD	wsld	knn	bagsvm	svm	rf
**raw**	0	2	58892	43748	75470	75336	2388	1988	424	72054	54458
**gm**	2	0	58892	43748	75470	75336	2390	1988	426	72054	54458
**mc**	58892	58892	0	30594	44054	37994	60444	60028	58884	45512	46272
**z**	43748	43748	30594	0	63636	49372	45532	45230	43730	57196	48668
**ppr**	75470	75470	44054	63636	0	33990	75938	75612	75436	32498	49678
**EGAD**	75336	75336	37994	49372	33990	0	76276	75832	75306	39848	48334
**wsld**	2388	2390	60444	45532	75938	76276	0	922	2422	72614	55114
**knn**	1988	1988	60028	45230	75612	75832	922	0	2042	72422	54872
**bagsvm**	424	426	58884	43730	75436	75306	2422	2042	0	72072	54456
**svm**	72054	72054	45512	57196	32498	39848	72614	72422	72072	0	41984
**rf**	54458	54458	46272	48668	49678	48334	55114	54872	54456	41984	0

genetic_OnlySeeds_SpearmanFootRule_vs_schizophrenia_OMNIPATH

	raw	gm	mc	z	ppr	EGAD	wsld	knn	bagsvm	svm	rf
**raw**	0	2	35312	26416	44218	44698	1418	906	452	35500	39402
**gm**	2	0	35314	26416	44218	44698	1418	906	452	35500	39402
**mc**	35312	35314	0	21518	30132	23718	36296	35784	35298	33900	23952
**z**	26416	26416	21518	0	42298	29582	27254	26878	26406	35664	31432
**ppr**	44218	44218	30132	42298	0	25162	44614	44148	44172	32390	26146
**EGAD**	44698	44698	23718	29582	25162	0	45266	44842	44702	31994	26210
**wsld**	1418	1418	36296	27254	44614	45266	0	872	1452	35526	40042
**knn**	906	906	35784	26878	44148	44842	872	0	988	35390	39640
**bagsvm**	452	452	35298	26406	44172	44702	1452	988	0	35524	39336
**svm**	35500	35500	33900	35664	32390	31994	35526	35390	35524	0	31980
**rf**	39402	39402	23952	31432	26146	26210	40042	39640	39336	31980	0

drugs_WithoutSeeds_SpearmanFootRule_vs_schizophrenia_STRING

	raw	gm	mc	z	ppr	EGAD	wsld	knn	bagsvm	svm	rf	COSNet
**raw**	0	0	8725	6850	6829	7091	6833	7276	692	8732	8683	9910
**gm**	0	0	8725	6850	6829	7091	6833	7276	692	8732	8683	9910
**mc**	8725	8725	0	6981	8272	9351	9175	9310	8718	9039	8007	10000
**z**	6850	6850	6981	0	5017	8644	8492	8842	6850	8734	9348	10000
**ppr**	6829	6829	8272	5017	0	7745	8146	8294	6832	8397	8952	10000
**EGAD**	7091	7091	9351	8644	7745	0	7679	7525	7231	7018	8696	9116
**wsld**	6833	6833	9175	8492	8146	7679	0	1427	6809	9230	9383	9679
**knn**	7276	7276	9310	8842	8294	7525	1427	0	7242	8969	9192	9667
**bagsvm**	692	692	8718	6850	6832	7231	6809	7242	0	8710	8674	9875
**svm**	8732	8732	9039	8734	8397	7018	9230	8969	8710	0	7435	10000
**rf**	8683	8683	8007	9348	8952	8696	9383	9192	8674	7435	0	10000
**COSNet**	9910	9910	10000	10000	10000	9116	9679	9667	9875	10000	10000	0

drugs_WithoutSeeds_SpearmanFootRule_vs_schizophrenia_OMNIPATH

	raw	gm	mc	z	ppr	EGAD	wsld	knn	bagsvm	svm	rf	COSNet
**raw**	0	0	7059	6078	7969	5841	3490	2852	1564	6314	6610	8400
**gm**	0	0	7059	6074	7969	5841	3490	2852	1564	6314	6610	8400
**mc**	7059	7059	0	6706	7511	7556	7621	7320	6987	7076	8108	9014
**z**	6078	6074	6706	0	6074	7925	6903	6665	6137	7193	7873	9432
**ppr**	7969	7969	7511	6074	0	8990	8553	8426	8046	8615	8937	9721
**EGAD**	5841	5841	7556	7925	8990	0	5611	5449	6034	7062	7973	7048
**wsld**	3490	3490	7621	6903	8553	5611	0	1138	3394	7193	7793	8201
**knn**	2852	2852	7320	6665	8426	5449	1138	0	2868	6877	7411	8189
**bagsvm**	1564	1564	6987	6137	8046	6034	3394	2868	0	6320	6777	8308
**svm**	6314	6314	7076	7193	8615	7062	7193	6877	6320	0	6573	9066
**rf**	6610	6610	8108	7873	8937	7973	7793	7411	6777	6573	0	9573
**COSNet**	8400	8400	9014	9432	9721	7048	8201	8189	8308	9066	9573	0

genetic_WithoutSeeds_SpearmanFootRule_vs_schizophrenia_STRING

	raw	gm	mc	z	ppr	EGAD	wsld	knn	bagsvm	svm	rf	COSNet
**raw**	0	0	9106	8606	9704	8543	3727	2955	1824	9529	9542	9423
**gm**	0	0	9106	8606	9704	8543	3727	2955	1824	9529	9542	9423
**mc**	9106	9106	0	4577	8279	9482	9867	9895	9293	9173	9704	9758
**z**	8606	8606	4577	0	7553	9703	9324	9363	8739	8994	9562	10000
**ppr**	9704	9704	8279	7553	0	9658	9686	9698	9706	6416	9741	10000
**EGAD**	8543	8543	9482	9703	9658	0	8509	8520	8473	9924	9824	6388
**wsld**	3727	3727	9867	9324	9686	8509	0	1501	3519	9704	9726	9347
**knn**	2955	2955	9895	9363	9698	8520	1501	0	3013	9505	9638	9368
**bagsvm**	1824	1824	9293	8739	9706	8473	3519	3013	0	9642	9486	9379
**svm**	9529	9529	9173	8994	6416	9924	9704	9505	9642	0	10000	10000
**rf**	9542	9542	9704	9562	9741	9824	9726	9638	9486	10000	0	8968
**COSNet**	9423	9423	9758	10000	10000	6388	9347	9368	9379	10000	8968	0

genetic_WithoutSeeds_SpearmanFootRule_vs_schizophrenia_OMNIPATH

	raw	gm	mc	z	ppr	EGAD	wsld	knn	bagsvm	svm	rf	COSNet
**raw**	0	0	8788	7514	9942	8663	1777	1049	1960	9791	9652	8845
**gm**	0	0	8788	7516	9942	8663	1777	1049	1958	9791	9652	8845
**mc**	8788	8788	0	7790	8750	8914	8966	8927	8713	9502	9744	8932
**z**	7514	7516	7790	0	8048	9756	8161	7752	7775	10000	10000	9786
**ppr**	9942	9942	8750	8048	0	10000	9933	9941	9942	10000	10000	10000
**EGAD**	8663	8663	8914	9756	10000	0	8732	8700	8607	9134	9928	864
**wsld**	1777	1777	8966	8161	9933	8732	0	1102	2360	9846	9756	8857
**knn**	1049	1049	8927	7752	9941	8700	1102	0	2021	9793	9634	8897
**bagsvm**	1960	1958	8713	7775	9942	8607	2360	2021	0	9790	9664	8871
**svm**	9791	9791	9502	10000	10000	9134	9846	9793	9790	0	5633	9109
**rf**	9652	9652	9744	10000	10000	9928	9756	9634	9664	5633	0	9930
**COSNet**	8845	8845	8932	9786	10000	864	8857	8897	8871	9109	9930	0
