# Supplementary material for: Benchmarking network propagation methods for disease gene identification
Source: PLoS Comput Biol. 2019 Sep 3;15(9):e1007276. doi: 10.1371/journal.pcbi.1007276 (PMC6743778; doi:10.1371/journal.pcbi.1007276)
Supplement: S2 File — Stand-alone viewer to explore models with interaction terms. (ZIP) [file pcbi.1007276.s003.zip › S2/interaction_results/branch-6.html]

partial\_auroc\_0.95

ALL cv\_scheme : method  
ALL disease : method  
ALL disease : network  
ALL network : method  
ALL input\_type : method  
ALL input\_type : disease  
ALL disease : network : method  
ALL disease : input\_type : method  
STRING + block disease : method  
STRING + block input\_type : method  
STRING + block input\_type : disease
