# Supplementary material for: Benchmarking network propagation methods for disease gene identification
Source: PLoS Comput Biol. 2019 Sep 3;15(9):e1007276. doi: 10.1371/journal.pcbi.1007276 (PMC6743778; doi:10.1371/journal.pcbi.1007276)
Supplement: S2 File — Stand-alone viewer to explore models with interaction terms. (ZIP) [file pcbi.1007276.s003.zip › S2/interaction_results/top.html]

interaction analysis: info | sqrt(top\_20\_hits) | sqrt(top\_100\_hits) | auroc | partial\_auroc\_0.90 | partial\_auroc\_0.95 | auprc | log10(auroc) | log10(auprc)
